# Exome sequencing of individuals with Huntington’s disease implicates FAN1 nuclease activity in slowing CAG expansion and disease onset

**DOI:** 10.1038/s41593-022-01033-5

**Published:** 2022-04-04

**Authors:** Branduff McAllister, Jasmine Donaldson, Caroline S. Binda, Sophie Powell, Uroosa Chughtai, Gareth Edwards, Joseph Stone, Sergey Lobanov, Linda Elliston, Laura-Nadine Schuhmacher, Elliott Rees, Georgina Menzies, Marc Ciosi, Alastair Maxwell, Michael J. Chao, Eun Pyo Hong, Diane Lucente, Vanessa Wheeler, Jong-Min Lee, Marcy E. MacDonald, Jeffrey D. Long, Elizabeth H. Aylward, G. Bernhard Landwehrmeyer, Anne E. Rosser, REGISTRY Investigators of the European Huntington’s disease network, Jane S. Paulsen, PREDICT-HD Investigators of the Huntington Study Group, Nigel M. Williams, James F. Gusella, Darren G. Monckton, Nicholas D. Allen, Peter Holmans, Lesley Jones, Thomas H. Massey

**Affiliations:** 1 Division of Psychological Medicine and Clinical Neurosciences, Cardiff University, Cardiff, UK; 2 School of Biosciences, Cardiff University, Cardiff, UK; 3 Institute of Molecular, Cell and Systems Biology, University of Glasgow, Glasgow, UK; 4 Molecular Neurogenetics Unit, Center for Genomic Medicine, Massachusetts General Hospital and Department of Neurology, Blavatnik Institute, Harvard Medical School, Boston, MA USA; 5 Medical and Population Genetics Program, Broad Institute, Cambridge, MA USA; 6 Departments of Psychiatry and Biostatistics, University of Iowa, Iowa City, IA USA; 7 Seattle Children’s Research Institute, Seattle, WA USA; 8 Department of Neurology, University of Ulm, Ulm, Germany; 9 Brain Repair Group, Schools of Medicine and Biosciences, Cardiff University, Cardiff, UK; 10 Neuroscience and Mental Health Research Institute, Cardiff University, Cardiff, UK; 11 University of Wisconsin, Madison, WI USA; 12 UK Dementia Research Institute at Cardiff, Cardiff University, Cardiff, UK

**Keywords:** Huntington's disease, Next-generation sequencing, Molecular neuroscience, Experimental models of disease

## Abstract

The age at onset of motor symptoms in Huntington’s disease (HD) is driven by *HTT* CAG repeat length but modified by other genes. In this study, we used exome sequencing of 683 patients with HD with extremes of onset or phenotype relative to CAG length to identify rare variants associated with clinical effect. We discovered damaging coding variants in candidate modifier genes identified in previous genome-wide association studies associated with altered HD onset or severity. Variants in FAN1 clustered in its DNA-binding and nuclease domains and were associated predominantly with earlier-onset HD. Nuclease activities of purified variants in vitro correlated with residual age at motor onset of HD. Mutating endogenous FAN1 to a nuclease-inactive form in an induced pluripotent stem cell model of HD led to rates of CAG expansion similar to those observed with complete *FAN1* knockout. Together, these data implicate FAN1 nuclease activity in slowing somatic repeat expansion and hence onset of HD.

## Abstract (web summary)

By using exome sequencing and extremes of phenotype, McAllister et al. identify rare coding variants with clinical effect in Huntington’s disease. They show that FAN1 nuclease activity slows CAG expansion and is associated with later onset of HD.

## Main

HD is an autosomal dominant neurodegenerative disorder affecting approximately one in 8,000 individuals. Neuronal loss in the brain leads to a progressive movement disorder alongside functionally debilitating neuropsychiatric and cognitive decline^1^. There is no disease-modifying treatment, and premature death typically occurs 10–30 years after symptom onset^2^.

HD is caused by an expanded CAG repeat tract of at least 36 trinucleotides in the *HTT* gene^3^. Longer inherited CAG repeat tracts are associated with earlier onset of motor symptoms but account for only ~50% of the observed variation^4–6^. Approximately 40% of the residual age at motor onset is heritable^7^, powering genome-wide association studies (GWASs) that have identified modifiers of disease course^8–10^. The most recent Genetic Modifiers of HD (GeM-HD) GWAS identified 21 independent signals at 14 genomic loci after accounting for CAG repeat length^8^. The most significant locus, on chromosome 15, includes *FAN1*, which encodes a structure-specific 5′ exo/endo-nuclease involved in interstrand crosslink (ICL) repair^11^. Candidate genes at other loci include *MSH3*, *MLH1*, *PMS1* and *LIG1*, all of which function in DNA repair. In addition, the pure CAG length of the pathogenic *HTT* repeat, rather than the length of encoded polyglutamine, is most strongly associated with motor onset^8,12,13^. A leading hypothesis is that somatic (non-germline) expansion of the pathogenic CAG repeat in susceptible brain neurons drives symptom onset. In support, the largest somatic expansions are observed in the striatal neurons that degenerate earliest in HD in both human brain^14,15^ and mouse HD models^16^, and the size of expansions in postmortem human HD cortex correlates with age at motor onset^17^. Furthermore, in mouse HD models, knockout of *Msh3*, *Mlh1* or *Mlh3* ablates somatic expansions of the CAG repeat^18,19^, whereas knockout of *Fan1* increases expansions^20^, in agreement with directions of effect predicted by human genetics.

GWASs can identify common genetic variation associated with a disease or trait. However, understanding pathogenic mechanisms through common variants is difficult: over 90% of GWAS signals are non-coding and mostly index gene expression changes^21^. The combined effect of all GWAS single-nucleotide polymorphisms (SNPs) usually accounts for a maximum of two-thirds of trait heritability, with genome-wide-significant SNPs contributing a small percentage^22^. Some of the missing heritability is due to rare, damaging, non-synonymous coding variants of larger effect size that are not well captured by GWAS. Identifying such variants has given direct insight into molecular pathogenesis in diseases such as schizophrenia^23^, Alzheimer’s disease^24^ and amyotrophic lateral sclerosis^25^. In this study, we sequenced the exomes of 683 patients with HD with extremes of phenotype relative to CAG repeat length and identified rare coding variant modifiers of disease.

## Results

### An HD study population with extremes of clinical phenotype

**Fig. 1:**
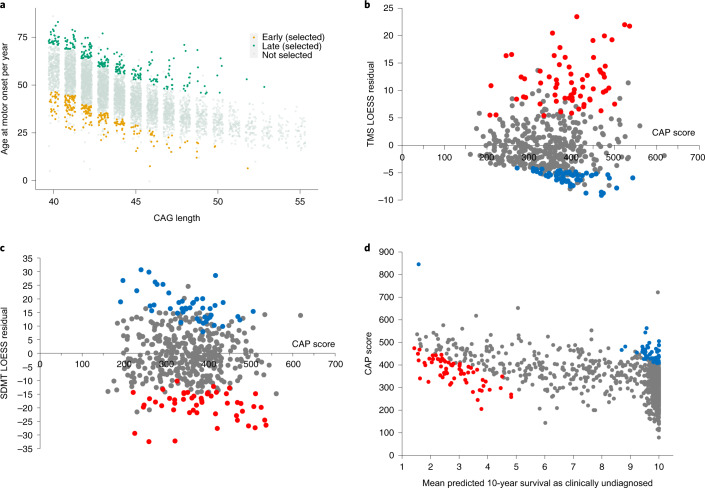
Selection of HD study population with extremes of onset or phenotype. **a**, REGISTRY-HD group. Age at motor onset against inherited pure CAG length for 6,086 patients with HD with 40–55 CAGs in the REGISTRY-HD study, using repeat lengths previously determined by PCR fragment length analysis. Individuals with very early (orange, *n* = 250) or very late (green, *n* = 250) motor onset given their inherited CAG length were selected for analysis. **b**, **c**, PREDICT-HD group, extremes of phenotype. Individuals with more severe (red dots) or less severe (blue dots) clinical phenotypes in the PREDICT-HD cohort were selected for analysis. Residuals from LOESS were used to identify individuals using TMS (*n* = 117) (**b**) or SDMT (*n* = 85) (**c**) and are plotted against CAP score to visualize age and CAG effects. Higher CAP scores represent greater disease burden. **d**, PREDICT-HD group, extremely early or late onset (predicted). A time-to-onset model was used to stratify the PREDICT-HD population and select a further cohort of predicted extreme early (red dots) or late (blue dots) onset individuals (*n* = 119 selected).

To maximize power to detect rare modifier variants in our cohort, we included individuals of European origin with extreme HD phenotypes from two independent longitudinal studies. First, we stratified 6,086 participants from the retrospective REGISTRY-HD study by residual age at motor onset, the difference between actual age at motor onset and that predicted by pure CAG length alone^26^, and selected ~4% at each extreme for investigation (Fig. 1a; 250 early onset, 250 late onset; ‘REGISTRY-HD group’). Second, we selected participants from the prospective PREDICT-HD study^27^ for investigation based on extreme cognitive or motor phenotypes, extreme predicted early or late onset or both (Fig. 1b–d; ~11% at each extreme; total *n* = 238/1,069; ‘PREDICT-HD group’). CAG lengths used in initial selections came from standard polymerase chain reaction (PCR) fragment length assays that assumed a canonical CAG repeat sequence.

### *HTT* repeat sequences are associated with altered HD onset

**Fig. 2:**
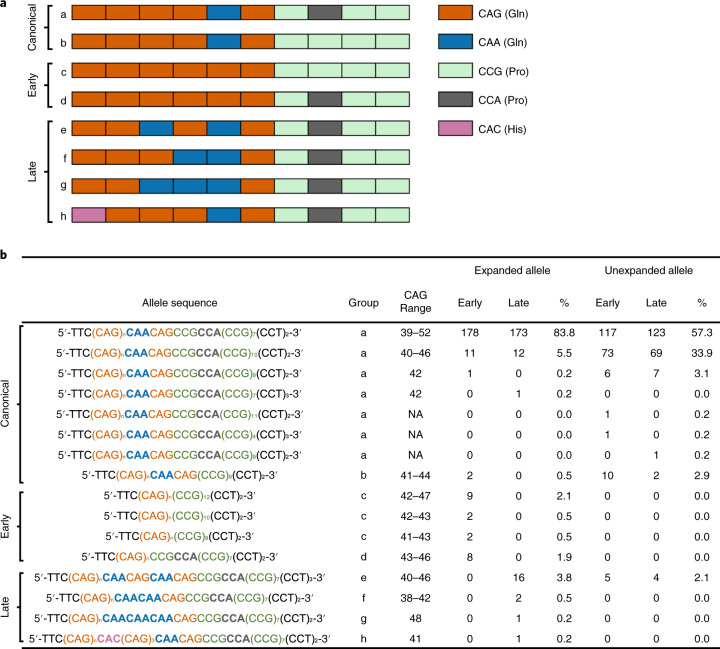
Non-canonical *HTT* CAG repeat sequences in expanded alleles are associated with altered onset of HD. **a**, Graphical overview of the 3′ end of the *HTT* exon 1 CAG repeat showing canonical (a–b) and non-canonical (c–h) trinucleotide arrangements identified by ultra-high-depth MiSeq sequencing in the REGISTRY-HD cohort. The clinical onset groups in which the non-canonical alleles were found are indicated on the left. Amino acids encoded by trinucleotides are shown on the right. Gln, glutamine; Pro, proline; His, histidine. **b**, *HTT* CAG repeat allele sequences and counts from ultra-high-depth MiSeq sequencing of the REGISTRY-HD cohort (*n* = 419 passing quality control; *n* = 213 with early onset relative to inherited pure CAG length; *n* = 206 with late onset relative to inherited pure CAG length. Note that CAG lengths are derived from MiSeq data). Allele counts for expanded (pathogenic) and unexpanded (wild-type) *HTT* alleles are shown, for both early-onset and late-onset groups. Allele groups refer to those illustrated in **a**. Interrupting trinucleotides within the CAG and CCG tracts are highlighted in bold. Range refers to pure CAG lengths in expanded alleles, where applicable. NA, not applicable.

The canonical polyglutamine-encoding CAG repeat in *HTT* exon 1 is followed by a glutamine-encoding CAACAG and then a polyproline-encoding CCG/CCA/CCT repeat (Fig. 2, allele groups a and b). This region of *HTT* 3′ of the CAG tract is highly polymorphic^8,12,13^. To investigate associations of this polymorphic sequence with motor onset, we sequenced the *HTT* CAG repeat locus in the REGISTRY-HD group using ultra-high-depth MiSeq sequencing. Samples from 419 individuals passed quality control and were associated with motor onsets at least 5 years earlier or later than expected after correcting for accurate CAG lengths from MiSeq^12^. We identified 16 independent *HTT* repeat structures downstream of the pure CAG repeat, eight of which occurred exclusively on pathogenic, expanded *HTT* alleles (Fig. 2b). A canonical CAACAG followed the pure CAG tract in 94% of all alleles (Fig. 2, allele groups a and b). The proportion of non-canonical glutamine-encoding repeats was enriched in alleles containing an expanded CAG repeat (41/419 (9.8%)) relative to those with an unexpanded repeat (9/419 (2.1%); χ^2^ = 21.8, *P* = 3.1 × 10^−6^; Fig. 2, allele groups c–h). Within the expanded alleles, the distributions of individual non-canonical sequences were highly skewed. Alleles lacking CAACAG were observed only in 21 individuals with early HD onset relative to their CAG tract lengths (Fig. 2, allele groups c and d; 21/213 early (9.9%) and 0/206 late, χ^2^ = 19.4, *P* = 1.0 × 10^−5^; mean earlier onset = 10.2 years). In contrast, alleles containing an extra CAACAG, CAA or CAC were observed only in 20 individuals with late HD onset relative to their CAG tract lengths (Fig. 2, allele groups e–h; 0/213 early and 20/206 late (9.7%), χ^2^ = 19.6, *P* = 9.0 × 10^−6^; mean later onset = 10.4 years).

GeM-HD GWAS reported SNPs tagging two non-canonical *HTT* repeat sequences: rs764154313 associated with (CAG)_n_(CCG)_12_(CCT)_2_ (Fig. 2b, group c, top) and rs183415333 associated with (CAG)_n_(CAACAG)_2_CCGCCA(CCG)_7_(CCT)_3_ (Fig. 2b, group e)^8^. Comparing GWAS SNP data to our *HTT* sequencing results in those individuals with both measures (*n* = 398), rs764154313 tagged six of the nine (CAG)_n_(CCG)_12_(CCT)_2_ alleles but no other allele lacking CAACAG. rs183415333 tagged (CAG)_n_(CAACAG)_2_CCGCCA(CCG)_7_(CCT)_3_ in 15/16 individuals carrying this sequence in an expanded allele. Overall, SNP data identified 28.6% (6/21) of alleles lacking CAACAG and 75.0% (15/20) of expanded alleles with an extra CAACAG, CAA or CAC, suggesting that non-canonical repeat alleles in HD are significantly underestimated by GWAS.

Much of the apparent effect of non-canonical glutamine-encoding *HTT* repeat sequences on HD onset has been considered spurious, attributable to inaccurate residual age-at-onset calculations based on assumed canonical allele sequences^8^. In our cohort, we found that polyglutamine length accounted for 31.0% (*P* = 7.2 × 10^−47^) of the variance in motor onset, rising to 37.2% (*P* = 3.1 × 10^−58^) when regressing on pure CAG length (Table 1; *n* = 558, 463 REGISTRY-HD and 95 PREDICT-HD). However, non-canonical *HTT* repeat sequences in expanded alleles remained significantly associated with age at onset even after accounting for pure CAG tract lengths. Expanded alleles lacking the CAACAG sequence were associated with ~16% decrease in age at motor onset, increasing *R*^2^ by 1.0% (*P* = 4.0 × 10^−3^). In contrast, expanded alleles with an extra CAACAG, CAA or CAC were associated with a ~26% increase in age at motor onset, increasing *R*^2^ by 1.4% (*P* = 2.0 × 10^−4^). Together, these effects increased *R*^2^ by 2.1% (Table 1). The effects of non-canonical repeat sequences on phenotype were also observed in a larger, dichotomous sample (logistic regression with Firth’s correction: *n* = 637; 421 REGISTRY-HD and 216 PREDICT-HD; cohort details below). Lack of the CAACAG sequence was significantly associated with early/more severe phenotype (*P* = 1.2 × 10^−7^). Additional CAACAG, CAA or CAC were significantly associated with late/less severe phenotype (*P* = 1.5 × 10^−6^). Therefore, changes in the canonical polyglutamine-encoding *HTT* sequences explain a small, but significant, proportion of the variance in HD onset not explained by CAG repeat length.

**Table 1:** The polyglutamine-encoding *HTT* repeat sequence is significantly associated with age at HD onset after accounting for pure CAG length

Model	Covariate	*B*	*P*	*R*^2^
Ln(AMO) ~ PolyQ length	PolyQ	−0.083	**7.15** **E-47**	0.310
Ln(AMO) ~ PolyCAG length	CAG	−0.092	**3.09 E-58**	0.372
Ln(AMO) ~ PolyCAG length + I_0	CAG	−0.091	**1.90 E-57**	0.382
	I_0	−0.177	**4.04 E-03**	
Ln(AMO) ~ PolyCAG length + I_2	CAG	−0.092	**2.67 E-59**	0.386
	I_2	0.233	**2.04 E-04**	
Ln(AMO) ~ PolyCAG length + I_0 + I_2	CAG	−0.091	**1.68 E-58**	0.393
	I_0	−0.168	**5.93 E-03**	
	I_2	0.225	**2.97 E-04**	

Linear regression of the natural logarithm of age at motor onset (AMO) on polyglutamine (PolyQ) or pure CAG (PolyCAG) length with or without covariates to represent non-canonical allele sequences. Covariate I_0 indicates the presence (1) or absence (0) of an expanded *HTT* allele lacking a CAACAG after the pure CAG tract (Fig. 2, allele groups c and d). Covariate I_2 indicates the presence (1) or absence (0) of an expanded *HTT* allele with an additional, non-canonical CAACAG or extra CAA or CAC trinucleotides (Fig. 2, allele groups e–h). All samples passing quality control that had been through ultra-high-depth MiSeq sequencing or had capillary electrophoresis (GeneScan) data and allele sequences confidently called from exome sequencing data were included (total *n* = 558; REGISTRY-HD *n* = 463 and PREDICT-HD *n* = 95 where motor onset has occurred). Significant *P* values after multiple testing correction for five models (*P* < 0.01) are in bold.

### *FAN1* coding variation is associated with altered onset of HD

**Extended Data Fig. 1:**
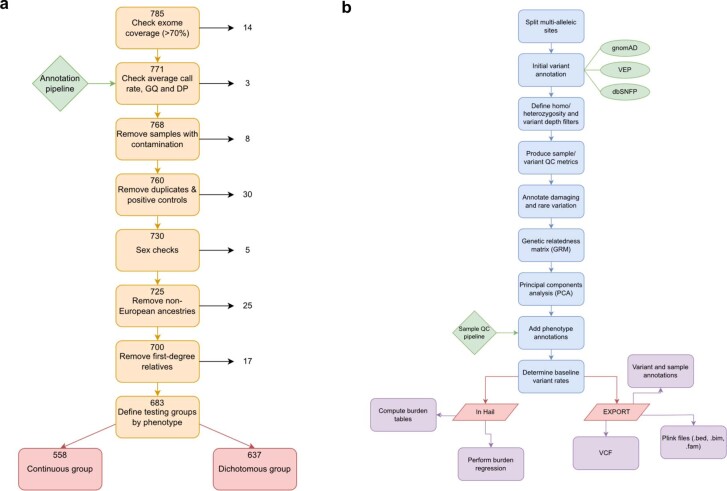
Quality control and annotation pipelines for HD exome sequencing data. **a**, Quality control pipeline showing where and why sequencing samples were removed from the dataset. From an initial 785 sequenced exomes, including some samples re-sequenced due to initial low quality, 683 passed all quality control steps (465 from REGISTRY-HD, 218 from PREDICT-HD). Subgroups of this population were used in downstream analyses: a continuous group (N = 558) containing all individuals with a known age at motor onset and a dichotomous group (N = 637) containing all individuals with an extreme phenotype, either early or late actual or predicted onset of symptoms, or more or less severe motor or cognitive symptom scores. See also Fig. 1 and Supplementary Fig. 1. **b**, Annotation pipeline indicating the pathway, databases (gnomAD & dbSNFP) and tools used to annotate individual variants across exomes. Key: VEP, variant effect predictor tool. See also Supplementary Fig. 2.

Using data from GeM-HD GWAS^8^, we calculated that all SNPs accounted for 25.3% (±4.5%) of the residual age at motor onset in HD compared to a reported heritability of 40%^7^. To investigate whether rare protein coding variants not captured by GWAS could account for some of this missing heritability, we used exome sequencing and called sequence variants (*n* = 683 after quality control, 465 REGISTRY-HD and 218 PREDICT-HD; Supplementary Figs. 1 and 2). Two groups of exomes were used for downstream association analyses, adjusted for accurate CAG repeat lengths from sequencing (Extended Data Fig. 1a). A dichotomous group (*n* = 637) was divided into early onset/more severe phenotype (*n* = 315) and late onset/less severe phenotype (*n* = 322), whereas a continuous phenotype group included only those with a calculable age at motor onset residual (*n* = 558). We assessed the association between rare non-synonymous coding variants (minor allele frequency (MAF) < 1%) and clinical phenotype in the 13 candidate modifier genes (other than *HTT)* identified in GeM-HD GWAS using logistic regression in the dichotomous group and linear regression in the continuous group. Independent analyses were performed with three groups of variants: all non-synonymous variants; non-synonymous, damaging variants (combined annotation-dependent depletion (CADD) score ≥ 20, indicating in top 1% of predicted damaging variants in the genome; NSD20 group); and loss-of-function variants such as nonsense, frameshift and splice donor/acceptor variants. Covariates corrected for population stratification, sequencing depth, baseline variant rate and study group (REGISTRY-HD or PREDICT-HD). The presence or absence of non-canonical glutamine-encoding *HTT* repeat sequences (Fig. 2 and Supplementary Table 1) was an additional covariate in logistic analyses. *FAN1* showed a significant signal in the dichotomous non-synonymous analysis after multiple testing correction (*P* = 2.3 × 10^−4^) and approached significance for NSD20 variants in both analysis groups (Table 2). The associations with non-synonymous and the NSD20 variants in the dichotomous group remained nominally significant (*P* = 3.8 × 10^−2^ and *P* = 4.4 × 10^−2^, respectively) even after the removal of the R377W and R507H variants previously identified in GeM-HD GWAS (Supplementary Table 3a). Furthermore, the burden of rare, damaging variants was not significantly associated with any of the lead *FAN1* variants from GeM-HD GWAS after removal of R377W and R507H (Supplementary Table 3b). Taken together, these results indicate that rare, damaging variation in FAN1 influences HD onset independently of modifiers previously identified by GWAS.

**Table 2:** Candidate gene analysis shows that rare coding variation in *FAN1* is associated with modified HD phenotype

		Dichotomous group (*n* = 637)			Continuous group (*n* = 558)		
Chr	Gene	NS	NSD20	LoF	NS	NSD20	LoF
2A	*PMS1*	*2.72E-02*	*2.65E-03*	3.13E-01	2.62E-01	1.08E-01	NA
3A	*MLH1*	1.72E-01	5.58E-02	NA	4.99E-01	7.45E-01	NA
5A	*DHFR*	1.05E-01	9.83E-02	NA	1.54E-01	1.42E-01	NA
5A	*MSH3*	5.96E-02	2.78E-01	*9.51E-03*	3.00E-01	6.35E-01	1.09E-01
5B	*TCERG1*	*1.25E-02*	4.54E-01	NA	*2.74E-03*	2.76E-01	NA
7A	*PMS2*	1.00E+00	9.00E-01	5.55E-01	5.59E-01	3.51E-01	1.33E-01
8A	*RRM2B*	1.69E-01	NA	NA	*2.26E-02*	NA	NA
8A	*UBR5*	7.47E-01	7.23E-01	NA	4.11E-01	3.88E-01	NA
11A	*CCDC82*	5.27E-01	NA	NA	6.09E-01	9.01E-01	NA
11B	*SYT9*	1.05E-01	1.13E-01	NA	4.20E-01	4.12E-01	NA
15A	*FAN1*	**2.32E-04**	*6.61E-04*	2.17E-01	*1.09E-03*	*9.06E-04*	3.40E-01
16A	*GSG1L*	9.05E-01	9.13E-01	NA	4.09E-01	4.35E-01	NA
19A	*LIG1*	9.47E-02	7.07E-02	5.09E-01	*3.63E-02*	*2.30E-02*	5.16E-01

SKAT-O of rare coding variants (MAF < 1%) and HD phenotypes for 13 candidate modifier genes from GeM-HD GWAS^8^. Gene-wide variant numbers were regressed on either dichotomous early/more severe or late/less severe phenotypes (*n* = 637; logistic regression) or a continuous phenotype of residual age at motor onset (*n* = 558; linear regression). Phenotypes were corrected for non-canonical *HTT* CAG repeats in expanded alleles by using either a covariate in logistic analyses or pure CAG lengths from sequencing in continuous analyses. Three different variant groups were tested: all non-synonymous (NS), non-synonymous and predicted damaging to protein function (NSD20; CADD PHRED score ≥ 20, indicating in the 1% predicted most damaging variants in the genome) and loss of function (LoF). Chromosomal loci from GeM-HD GWAS are indicated. Significant associations are in bold (*P* < 6.4 × 10^−4^, Bonferroni correction for 13 genes and six tests); nominally significant associations are in italics (*P* < 0.05). See also Supplementary Table 4. NA, not applicable due to insufficient variation in the study population.

Five other candidate modifier genes had nominally significant associations in at least one analysis: *PMS1*, *MSH3*, *TCERG1*, *RRM2B* and *LIG1* (Table 2) and were the most significant at their respective genomic loci when assessing all genes (Supplementary Table 4). There were marked skews in the distribution of predicted damaging coding variants in *FAN1*, *PMS1* and *MSH3*: those in *FAN1* were often associated with early/more severe phenotype, whereas those in *PMS1* and *MSH3* were often associated with late/less severe phenotype (Supplementary Table 5). Rare, damaging coding variants in *PMS1* occurred in 14 individuals with late/less severe phenotype, including one loss-of-function frameshift variant, but in just two individuals with early/more severe disease (Supplementary Table 6). Although the distribution of predicted deleterious *MSH3* variants was less skewed (eight in the early/more severe group and 14 in the late/less severe group; Supplementary Table 5), loss-of-function variants were found exclusively in seven individuals with late/less severe phenotype. These *MSH3* variants were all extremely rare and included four splice acceptor variants and three truncations.

### Exome-wide association analysis highlights *FAN1*

We explored whether an exome-wide analysis of all genes with sufficient rare, damaging variation (NSD20) would highlight new HD modifiers. Analysis of the dichotomous and continuous phenotype groups did not show any significant exome-wide associations (Supplementary Table 7). *FAN1* was the only gene in the top ten of both dichotomous and continuous analyses (*P* < 1.0 × 10^−3^ in each case). A candidate pathway analysis, using significant pathways (*q* < 1.0 × 10^−3^) from GeM-HD GWAS, identified one significant pathway (GO:0042578; phosphoric ester hydrolase activity, containing *FAN1*; *P* = 9.7 × 10^−5^) and several nominally significant pathways, suggesting that damaging, rare variation may be important in pathways previously associated with common variation (Supplementary Table 8). For example, GO:0006281 (DNA repair) is significantly enriched for damaging, rare variation in the dichotomous analysis (*P* = 1.2 × 10^−3^) and also for common variant association in GeM-HD GWAS (*P* = 5.4 × 10^−11^).

### FAN1 variants that modify HD cluster in functional domains

**Fig. 3:**
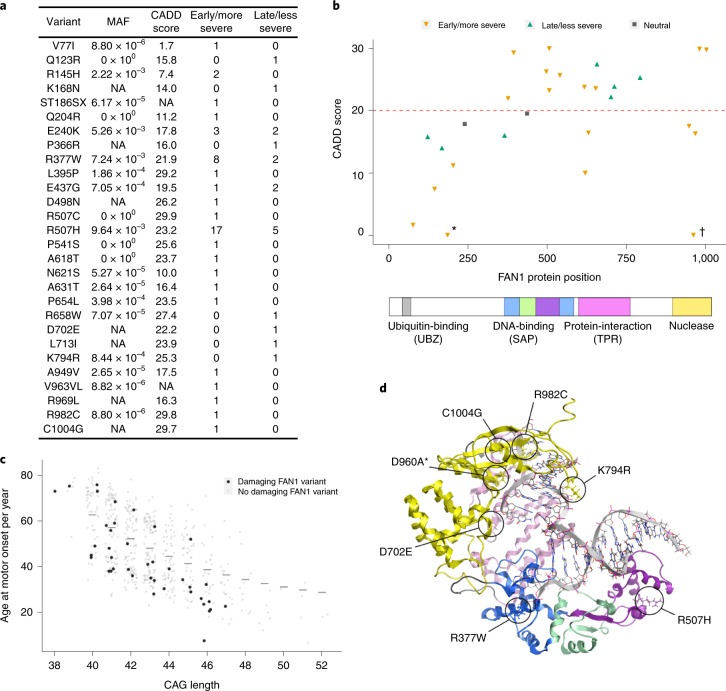
Rare deleterious FAN1 variants are associated with altered HD onset and cluster in functional protein domains. **a**, Rare, non-synonymous *FAN1* variants identified through exome sequencing in the dichotomous HD cohort (*n* = 637), divided between early/more severe and late/less severe phenotype groups. MAF < 1%. A total of 65 such variants (28 different) were identified across 62 individuals. Three people carried two variants. CADD score is a measure of predicted deleteriousness of a coding variant. CADD ≥ 20 implies that a variant is in the 1% predicted most damaging substitutions in the human genome. A total of 43 individuals carried at least one such predicted damaging variant, with two people carrying two (although these could not be phased). **b**, FAN1 variants identified in individuals with HD, plotted by CADD score over a cartoon of FAN1 protein. Variants associated mostly with early/more severe phenotype (orange triangles), late/less severe phenotype (green triangles) or neither phenotype group (gray squares, ‘neutral’) are shown. Variants above the CADD = 20 line are predicted to be in the top 1% most damaging variants in the human genome; those with CADD > 10 are predicted to be in the top 10%. Two likely damaging singleton variants lack CADD scores and so are plotted as CADD = 0. They are highlighted: loss-of-function (frameshift) variant ST186SX (*) and in-frame insertion variant V963W964insL (†). FAN1 domain coordinates as published^51,52^. **c**, Damaging FAN1 variants are enriched in individuals with earlier-onset HD after accounting for CAG length. Age at motor onset against CAG length is plotted for the continuous phenotype group (*n* = 558), with population predicted age at onset for each repeat length shown with horizontal lines^26^. No median onset is shown for CAG lengths of 38 and 39 as they are incompletely penetrant. Individuals with a damaging FAN1 variant (CADD ≥ 20 or loss of function) are shown as black dots; those without one are shown as open circles. **d**, Three-dimensional model highlighting FAN1 variants selected for downstream study. Note that D960A (*) is a synthetic variant lacking nuclease activity not found in our patient population. NA, not applicable.

*FAN1* is the most significant gene in both common variant modifier GWAS and our candidate exome analysis. In our cohort, we identified 65 rare non-synonymous FAN1 variants across 62 individuals, including 28 different variants (Fig. 3a) and six previously unreported mutations (K168N, P366R, D498N, D702E, L713I and R969L). Focusing on those variants predicted to be most damaging to protein function (CADD score ≥ 20 or loss of function), we found that 6.8% of our cohort (43/637) were heterozygous for at least one such variant in *FAN1* (Fig. 3a,b). Two individuals carried two such variants, although these could not be phased. One individual carried a loss-of-function frameshift variant, ST186SX (15:31197095:G:A). Of those carrying damaging *FAN1* variants, significantly more had an early/more severe phenotype than a late/less severe phenotype (odds ratio = 3.43, 95% confidence interval, 1.66–7.09, *P* = 8.9 × 10^−4^; Fig. 3c). Those variants associated mostly with early/more severe HD clustered in the DNA-binding and nuclease domains of FAN1, whereas a small cluster of variants associated with late/less severe HD mapped to the protein–protein interaction domain (Fig. 3b,d). The R377W and R507H variants detected in GeM-HD GWAS, and also found here, map on the DNA-binding domain of FAN1 and are found mostly in individuals with early/more severe disease. Of the seven individuals with late/less severe HD but carrying FAN1 R377W or R507H, one had an extra CAACAG in the expanded *HTT* repeat tract. Five were also genotyped in GeM-HD GWAS^8^: one was homozygous and two were heterozygous for the common I219V variant in MLH1 (rs1799977) associated with later-onset HD; one had high predicted FAN1 expression and one had low predicted *MSH3* expression, consistent with later-onset HD. Finally, five extremely rare (MAF < 1.0 × 10^−6^) damaging variants in the C-terminal nuclease domain were exclusively associated with early/more severe disease. Overall, these data implicate FAN1 DNA-binding and nuclease activities in delaying HD onset.

### FAN1 variant nuclease activity correlates with HD onset

**Fig. 4:**
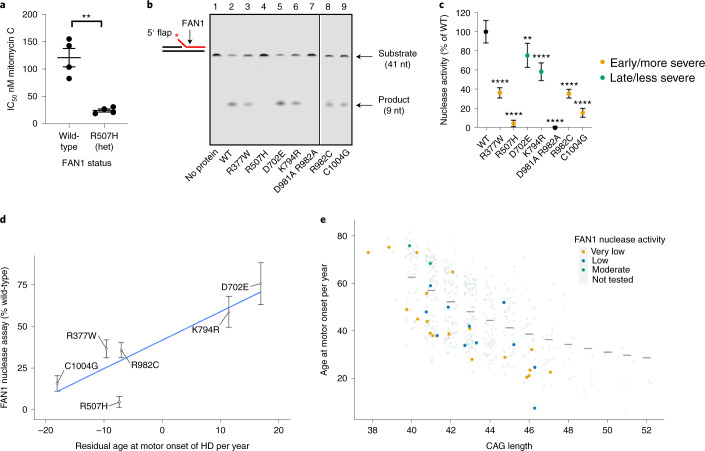
Nuclease activity of FAN1 variants identified in individuals with HD correlates with residual age at onset of motor symptoms. **a**, Lymphoblastoid cell lines derived from patients carrying a heterozygous R507H FAN1 variant are significantly more sensitive to mitomycin C than age-matched and pure CAG-length-matched control HD lines with homozygous wild-type FAN1 (**P = 1.3 × 10^−2^, two-tailed *t*-test). Four independent lines for each genotype, mean of three independent experiments shown for each line (dots), as well as mean ± s.e.m. for each genotype (horizontal lines). **b**, Representative gel showing nuclease activity of FAN1 variants on 5′ flap DNA. FAN1 protein (10 nM) was incubated with fluorescently labeled 5′ flap DNA substrate (5 nM) for 10 minutes at 37 °C in the presence of MnCl_2_. Reactions were denatured and analyzed using 15% TBE-urea gel electrophoresis. All experiments were repeated at least three times. **c**, FAN1 variants identified in individuals with HD have significantly reduced nuclease activity compared to wild-type FAN1. Variants associated with early/more severe phenotypes (orange) had less nuclease activity than variants associated with late/less severe phenotypes (green). The nuclease-inactive D981A R982A FAN1 variant was used as a negative control. Activities of variants are normalized to wild-type FAN1 nuclease activity. (*n* = 3 independent experiments, **P < 1 × 10^−2^ and ****P < 1 × 10^−4^; one-way ANOVA; mean ± s.d. shown). **d**, Graph of mean age at motor onset residual (using pure CAG length from sequencing) against FAN1 nuclease activity for six variants, normalized to wild-type FAN1 activity: R377W *n* = 10; R507H *n* = 18; D702E *n* = 1; K794R *n* = 1; R982C *n* = 1; C1004G *n* = 1. There was a significant correlation between average motor onset residual and in vitro nuclease activity (*P* = 2.7 × 10^−2^). Mean ± s.d. nuclease activity is shown for each variant (*n* = 3 independent experiments). Three individuals had two FAN1 variants: C1004G and R507H; R982C and N621S; and R377W and R507H. For analyses, these individuals were included in the groups of the most damaging of the two variants they carried. See also Supplementary Fig. 3. **e**, Graph of age at motor onset against CAG length for the continuous phenotype group (*n* = 558), highlighting those individuals carrying damaging FAN1 variants assayed for nuclease activity. FAN1 nuclease activity: very low (<20% of wild-type), low (20–50% of wild-type) and moderate (50–80% of wild-type). nt, nucleotide; WT, wild-type. Source data

The nuclease activity of FAN1 is required for its functions in DNA repair but had no significant effect on CAG repeat expansion in a U2OS cell model system^28,29^. We first assessed whether four lymphoblastoid cell lines derived from individuals with HD heterozygous for the R507H FAN1 variant were as efficient in ICL repair as four age-matched and CAG-length-matched controls with wild-type *FAN1* alleles. Each R507H line was more sensitive to mitomycin C than its matched counterpart (Supplementary Fig. 3a), and, overall, the mean IC_50_ for the R507H lines was significantly lower (Fig. 4a; *P* = 1.3 × 10^−3^), implying that R507H is deleterious to FAN1 function, as previously suggested^30,31^. Next, we selected six predicted deleterious FAN1 variants identified by exome sequencing for in vitro biochemical analysis of nuclease activity. These variants (R377W, R507H, D702E, K794R, R982C and C1004G), as well as wild-type FAN1 and a known nuclease-inactive variant (D981A R982A^11^), were expressed and partially purified from *Escherichia coli* as NusA-His-tagged full-length proteins (Supplementary Fig. 3b,c). The flap endonuclease activities of wild-type and variant FAN1 proteins were assayed on canonical FAN1 substrates with short 5′ flaps. Wild-type FAN1 converted 66% of substrate to product in a 10-minute reaction (Fig. 4b, lane 2). The nuclease-inactive double mutant lacked all nuclease activity, as expected (Fig. 4b, lane 7). The predicted damaging FAN1 variants all had reduced nuclease activity compared to wild-type (Fig. 4c), but variants associated mostly with early/more severe phenotype (R377W, R507H, R982C and C1004G) had much reduced activity compared to the two variants found in individuals with late/less severe phenotype (D702E and K794R). There was a significant correlation between mean residual age at onset for individuals harboring each variant and nuclease activity of that variant (*P* = 2.7 × 10^−2^; Fig. 4d), suggesting that FAN1 nuclease activity mediates its modifier role in HD. Individuals carrying a FAN1 variant with low nuclease activity (<50% of wild-type) are more likely to have early-onset HD relative to that predicted by CAG length alone (Fig. 4e). The low FAN1 nuclease activity in the two individuals with 38 and 39 CAGs, respectively, might be contributing to the full penetrance of these *HTT* alleles.

### FAN1 requires nuclease activity to slow CAG repeat expansion

**Fig. 5:**
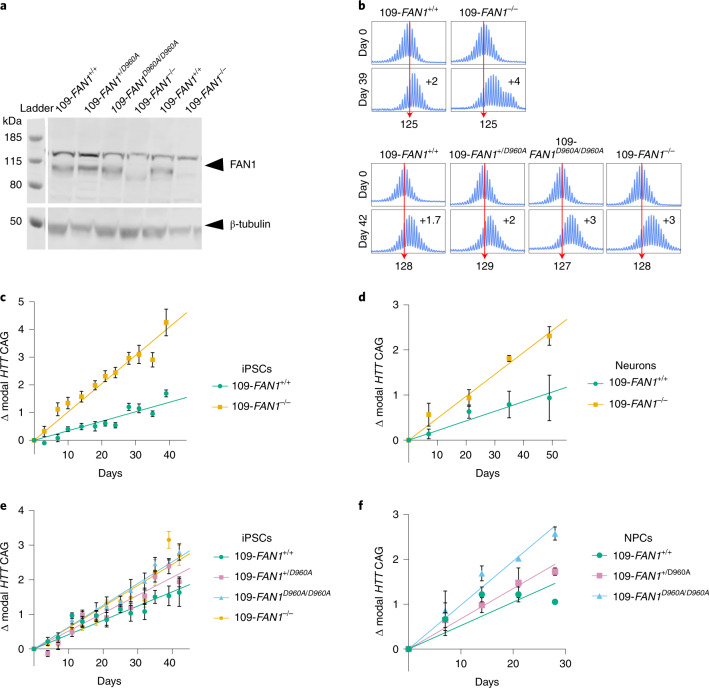
FAN1 slows the rate of *HTT* CAG repeat expansions in a nuclease-dependent manner in an iPSC model of HD. **a**, Immunoblot of FAN1 in Q109 HD iPSC lines. Parent Q109 lines with wild-type FAN1 (Q109-n5 (lane 1) and Q109-n1 (lane 5)); Q109 lines with D960A variant (heterozygous (lane 2) and homozygous (lane 3)); and Q109 *FAN1* knockout lines (Q109-n5 (lane 4) and Q109-n1 (lane 6)). Blots were repeated twice to confirm results. **b**, Representative electropherograms of fluorescent PCR and capillary electrophoresis of the *HTT* CAG repeat in 109-*FAN1*^*+/+*^, 109-*FAN1*^−*/*−^, 109-*FAN1*^*+/D960A*^ and 109-*FAN1*^*D960A/D960A*^ iPSCs at baseline and after time in culture. The red dotted line indicates baseline *HTT* CAG repeat length. **c**, 109-*FAN1*^−*/*−^ iPSCs (*n* = 6 clones) exhibit significantly faster *HTT* CAG repeat expansion rates than 109-*FAN1*^*+/+*^ iPSCs (*n* = 7 clones) (0.0661 CAG per day) (*P* = 1.5 × 10^−5^). Genome editing performed in Q109-n1 iPSCs. **d**, Change in modal *HTT* CAG repeat in post-mitotic neurons generated from 109-*FAN1*^*+/+*^ iPSCs (*n* = 5) and 109-*FAN1*^−*/*−^ iPSCs (*n* = 4 clones). 109-*FAN1*^−*/*−^ neurons demonstrate significantly faster rates of *HTT* CAG repeat expansion than 109-*FAN1*^*+/+*^ neurons (*P* = 1.1 × 10^−2^). **e**, *FAN1*^*D960A*^ HD-iPSCs show dose-dependent increase in *HTT* CAG repeat expansion over time in culture (*n* = 3 clones per genotype, each cultured in triplicate wells). Genome editing performed in Q109-n5 iPSCs. **f**, D960A mutations enhance *HTT* CAG repeat expansions in a dose-dependent manner in NPCs derived from 109-*FAN1*^*+/+*^, 109-*FAN1*^*+/D960A*^ and 109-*FAN1*^*D960A/D960A*^ iPSCs (*n* = 1 clone per genotype, cultured in triplicate wells). Values are expressed as mean ± s.e.m. Source data

**Extended Data Fig. 2:**
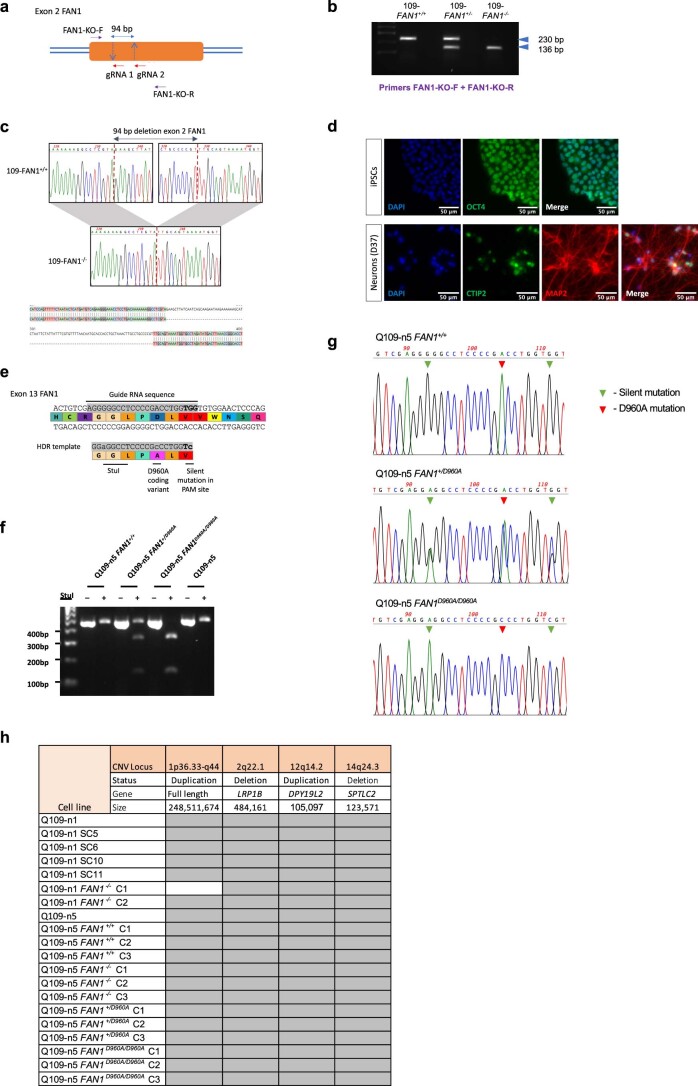
*FAN1* knockout and D960A editing using CRISPR-Cas9. **a**, Schematic depicting CRISPR-Cas9 targeting of exon 2 of *FAN1* in Q109-n1 and Q109-n5 using two guide RNAs (gRNAs) to induce a 94 bp deletion leading to a premature stop codon and *FAN1* knockout. The primer pair used for PCR screening of exon 2 after CRISPR is also shown. **b**, Diagnostic PCR screen using primers FAN1-KO-F and FAN1-KO-R showing representative banding patterns for iPSC lines with the three possible *FAN1* genotypes after CRISPR: *FAN1*^+/+^ (230/230 bp; wild-type), *FAN1*^+/−^ (230/135 bp) and *FAN1*^−/−^ (135/135 bp). **c**, Sanger sequencing of PCR products demonstrates the targeted 94 bp deletion in exon 2 of FAN1. **d**, Undifferentiated iPSCs stained for the pluripotency marker OCT4. iPSC-derived neurons stained positive for the neuronal marker MAP2 (red) and CTIP2 (green). All nuclei are counterstained with DAPI (blue). **e**, Schematic depicting CRISPR-Cas9 targeting of exon 13 of *FAN1* in Q109-n5 using a homology directed repair (HDR) template. A single guide RNA sequence (grey) and a 122 bp HDR template containing the desired gene edit coding for an amino acid change (D960A) were utilised to generate FAN1-nuclease dead clones. The HDR template contained two silent mutations (lowercase) to prevent Cas9 re-cutting of the edited region and to introduce a StuI restriction site for diagnostic screening. **f**, Restriction digest with StuI confirms Q109-n5 *FAN1*^+/+^, Q109-n5 *FAN1*^+*/D960A*^ and Q109-n5 *FAN1*^*D960A/D960A*^ genotypes. StuI cleaves the 442 bp PCR product into 124 and 318 bp products only in the presence of the silent 2868 G > A mutation. The parental Q109-n5 line is also shown as a negative control (right). **g**, Sanger sequencing of PCR products confirms successful introduction of D960A variant. **h**, Virtual karyotyping of iPSC lines using copy number variant (CNV) analysis at the time of repeat expansion experiments. CNV analysis reveals small deletions at 2q22.1 and 14q24.3 and duplications at 12q14.2 in all samples. Duplication of chromosome 1 shown in all but one sample. Source data

Only one HD-induced pluripotent stem cell (iPSC) line, reprogrammed using lentivirus from a patient with 109 CAGs, has previously demonstrated inherent and reproducible *HTT* CAG expansion in culture^32^. To identify isogenic lines for assaying repeat expansion, we first confirmed CAG repeat expansion in three further independent iPSC lines derived from the same patient (but reprogrammed using non-integrating vectors): CS09iHD109-n1, CS09iHD109-n4 and CS09iHD109-n5 (ref. ^33^). CAG repeat lengths ranged from 92 to 122, indicative of a mosaic population and CAG repeat instability, and expanded over time in culture (Supplementary Fig. 4). To establish isogenic iPSC models of *HTT* CAG repeat expansion, the parent CS09iHD109-n1 and CS09iHD109-n5 (Q109) lines were used for CRISPR genome editing. We investigated the effect of FAN1 by measuring CAG expansion rates in multiple independent isogenic Q109 sub-clones with and without *FAN1* knockout (Fig. 5 and Extended Data Fig. 2a–c). The *HTT* CAG repeat expanded significantly faster in Q109-n1 iPSCs lacking *FAN1*: each modal CAG unit increase occurred in 8.9 days in *FAN1*^−/−^ cells compared to 33.1 days in *FAN1*^+/+^ cells (*P* = 1.5 × 10^−5^; Fig. 5b,c). Expansion in differentiated neurons (Supplementary Fig. 4d) showed a similar effect, although expansion rates were slower (Fig. 5d). Each CAG unit increase occurred in 20.6 days in *FAN1*^−/−^ neurons compared to 73.0 days in *FAN1*^+/+^ cells (*P* = 2.7 × 10^−5^). Despite the slower rate of CAG expansion seen in neurons compared to iPSCs, the ratio of expansion rates *FAN1*^+/+^:*FAN1*^−/−^ was remarkably similar at 3.7× in iPSCs and 3.5× in neurons.

To investigate the importance of FAN1 nuclease activity in slowing *HTT* CAG repeat expansion, we used CRISPR to introduce a D960A FAN1 point mutation into Q109-n5 iPSCs (Extended Data Fig. 2e–g). D960 coordinates an essential divalent cation, usually Mg^2+^, in the nuclease active site of FAN1. Mutation to D960A is well established to abolish nuclease activity but retain wild-type-DNA-binding capacity^34,35^, allowing the role of FAN1 nuclease activity to be specifically assayed. Somatic instability of the *HTT* CAG tract was then assessed in *FAN1*^+/+^, *FAN1*^+^^*/D960A*^, *FAN1*^*D960A/D960A*^ and *FAN1*^−/−^ Q109-n5 iPSCs (Fig. 5e) and showed a nominally significant difference between genotypes (*P* = 4.5 × 10^−2^). Cells carrying homozygous or heterozygous *FAN1*^*D960A*^ alleles had significantly greater rates of change in modal CAG length compared to wild-type cells (*P* = 2.3 × 10^−2^), but there was no detectable difference among Q109 FAN1^+*/D960A*^, *FAN1*^*D960A/D960A*^ and *FAN1*^*−/−*^ cells (*P* = 2.3 × 10^1^). Adding one CAG took 23.9 days in *FAN1*^+/+^ cells but 19.3 days in *FAN1*^+*/D960A*^ cells and 15.6 and 16.3 days in *FAN1*^*D960A/D960A*^ and *FAN1*^*−/−*^ cells, respectively. These data suggest an additive effect of FAN1 nuclease activity on expansion.

We also found a significant difference between genotypes in rates of expansion in a single clone of each *FAN1*^*D960A*^ genotype in neural precursor cells (NPCs) grown for 42 days (Fig. 5f). A significant effect of genotype on rate of change in modal CAG was observed (*P* = 6.0 × 10^−3^). Adding one CAG took 19.1 days in *FAN1*^+/+^ cells, 14.8 days in *FAN1*^+*/D960A*^ cells and 10.8 days in *FAN1*^*D960A/D960A*^ cells. The rate of CAG expansion in *FAN1*^+*/D960A*^ cells was significantly lower than that in *FAN1*^*D960A/D960A*^ cells (*P* = 6.0 × 10^−3^), whereas the rate of CAG expansion in *FAN1*^+/+^ cells trended toward being lower than that in *FAN1*^+*/D960A*^ cells (*P* = 6.6 × 10^−2^). Fitting an additive model of D960A to CAG expansion rates increases significance over the general model (*P* = 3.0 × 10^−3^), suggesting that there is a dose-dependent effect of the D960A mutation on CAG expansion rate, consistent with the effect seen in iPSCs.

## Discussion

Inherited pathogenic *HTT* CAG repeat length is a stronger determinant of age at onset of HD than polyglutamine length^8,12,13^. Here we have additionally shown that non-canonical glutamine-encoding *HTT* repeat sequences are significantly associated with HD onset, even after correcting for accurate CAG repeat lengths (Table 1). Expanded repeat tracts lacking the canonical 3′ CAACAG were exclusively associated with earlier-onset disease; tracts with extra CAACAG, CAA or CAC were exclusively associated with later-onset disease. GeM-HD GWAS suggested that much of this association is spurious, attributable to mis-sizing of CAG tracts when a canonical repeat sequence is assumed^8^. The difference in results probably arises from both our extreme phenotype study design, enriching for rare, non-canonical sequences of large effect size, and the underestimation of non-canonical *HTT* repeat sequences by the SNP genotyping used in GWAS. Non-canonical repeats likely mediate their effects through altered somatic expansion of the genomic pathogenic CAG repeat, with tracts lacking CAACAG expanding more easily and those with extra CAACAG, CAA or CAC expanding less easily. However, alternative mechanisms, including effects on splicing or translation of RNA, and the influence of flanking repeats, are possible.

DNA repair genes that modify HD onset are also likely to function through modulation of somatic expansion of the *HTT* CAG repeat in the brain^36^, although an effect on global genomic stability cannot be discounted. Assessing candidate modifier genes from GeM-HD GWAS^8^ through exome sequencing, we identified significant numbers of rare, predicted deleterious coding variants in *FAN1* and nominally significant numbers in *PMS1*, *MSH3*, *TCERG1*, *RRM2B* and *LIG1* (Table 2). Directions of effect were consistent with GeM-HD GWAS, further implicating these genes as the disease modifiers at their respective loci. Although variant effect sizes in the wider HD population cannot be determined from our extreme phenotype cohort, they are likely to be large. For example, GeM-HD GWAS showed that each *FAN1*^*R507H*^ or *FAN1*^*R377W*^ allele is associated with 5.2 years or 3.8 years earlier onset of HD, respectively^8^.

The coding variants we identify are heterozygous in affected individuals, suggesting that either wild-type protein levels are rate limiting for somatic expansion and the variants are hypomorphic or that the variants have dominant-negative or gain-of-function effects. Transcriptome-wide association studies in HD have implicated expression levels of *FAN1* and *MSH3* in modification of motor onset and progression, respectively, probably through effects on somatic expansion^8,28,37^. Notably, the seven individuals carrying a heterozygous loss-of-function variant in *MSH3* here all had a late/less severe phenotype. These variants might limit or disrupt the formation of the MSH2/MSH3 (MutSβ) heterodimers that are required to bind and stabilize slipped DNA structures formed by trinucleotide repeats before repeat expansion^38,39^. Rare variants in *PMS1* were also mostly found in patients with late/less severe phenotype (Supplementary Table 5), implicating PMS1 in repeat expansion, as previously suggested in a murine cell model of Fragile X disorders^40^. PMS1 forms a heterodimer with MLH1 (MutLβ), but, unlike PMS2 and MLH3, the other two binding partners of MLH1, PMS1 lacks a known catalytic activity and does not participate directly in mismatch repair^41^. It remains unclear how PMS1 might facilitate repeat expansions.

Previous genetic studies have strongly implicated *FAN1* as the HD modifier gene at the chromosome 15 locus, with at least four independent GWAS signals, including the variants encoding R377W and R507H^8,30^. Our exome-wide association study highlighted *FAN1* as the only gene with *P* < 1.0 × 10^−3^ in both dichotomous and continuous analyses (Supplementary Table 7), showing that its association is robust to which HD phenotypes are used. Removing the variants encoding R377W and R507H reduced, but did not ablate, the significance of these associations. Furthermore, the remaining burden of rare, damaging variation in *FAN1* in our cohort was not associated with known GWAS signals (Supplementary Table 3b), providing more evidence that rare, damaging variation in *FAN1* modifies HD onset in addition to genetic effects captured by GWAS. The rare, predicted damaging variant burden in *FAN1* was three-fold higher than expected from population frequencies (gnomAD^42^), further suggestive of a modifier effect. Notably, although *FAN1*^*R377W*^ and *FAN1*^*R507H*^ are mostly found in those with early/more severe phenotype, they are also seen in seven individuals with late/less severe phenotype (Fig. 3), suggesting that the effects of these variants are themselves modifiable. Indeed, six of these individuals carried an additional modifier associated with later HD onset.

FAN1 has cellular functions in ICL repair, replication fork restart and maintenance of genomic stability^11,43,44^, each of which requires its structure-specific nuclease activity. The clustering of rare modifier variants in FAN1 domains provides new insight into how FAN1 might modify HD onset. One cluster between residues 377 and 654 contains 11 different variants that are found in 34 individuals with early/more severe phenotype and nine individuals with late/less severe phenotype (Fig. 3). Variants in this cluster might affect DNA binding or FAN1 dimerization, mediated by the SAP domain (residues 462–538) and a dimerization loop (residues 510–518), respectively. The most common variant in this cluster, R507H, reduces FAN1 DNA binding in vitro^30^. We show that R507H sensitizes dividing cells to mitomycin C (which induces ICLs) and also reduces nuclease activity, fitting human phenotypic data, suggesting that R507H FAN1 has impaired function and clinical effect^31^.

A second cluster contains four different variants in the protein–protein interaction domain (residues 658–794) associated with late/less severe phenotype (Fig. 3). Surprisingly, these variants seem to act in the opposite direction to most damaging FAN1 variants. Potential mechanisms could include enhancement of DNA binding or nuclease activities of FAN1 or modulation of protein–protein interactions at a CAG repeat. Given that FAN1 interacts with MLH1, MLH3 and PMS1 (ref. ^45^), all of which promote repeat expansions, variants in the FAN1 protein–protein interaction domain could favor sequestration of these proteins and indirectly inhibit somatic expansion^29,46^.

A final cluster contains five different deleterious variants in the C-terminal nuclease domain (residues 895–1,017), associated exclusively with early/more severe phenotype and implicating FAN1 nuclease activity in modification of somatic expansion and HD onset. In agreement, inactivation of FAN1 nuclease (D960A) increased the rate of CAG repeat expansion in Q109 HD iPSCs (Fig. 5e). Notably, homozygous D960A FAN1 stimulated repeat expansion to the same extent as homozygous *FAN1* knockout, strongly suggesting that its nuclease activity is required for FAN1 to slow repeat expansion. This notion is strengthened by the significant correlation between the nuclease activity of purified FAN1 variants and the residual age at motor onset of HD in individuals carrying those variants (Fig. 4). In contrast, published data from a *FAN1*^−*/*−^ osteosarcoma cell line transduced with a 118 CAG repeat showed that overexpression of wild-type, D960A or R507H FAN1 was equally effective at slowing repeat expansion^28^. These apparently contradictory findings can be reconciled if non-physiological overexpression of FAN1, or its variants, leads to binding, sequestration and effective inactivation of expansion-promoting proteins, such as PMS1, MLH1 and MLH3 (refs. ^19,40^). Conversely, when FAN1 is expressed from its endogenous promoter, it could be rate limiting for repeat stability and require its nuclease activity to prevent expansions. Recent evidence highlighting how FAN1–MLH1 interaction might promote accurate repair of DNA loop-outs and slow repeat expansion fits such a model^29,46^.

**Fig. 6:**
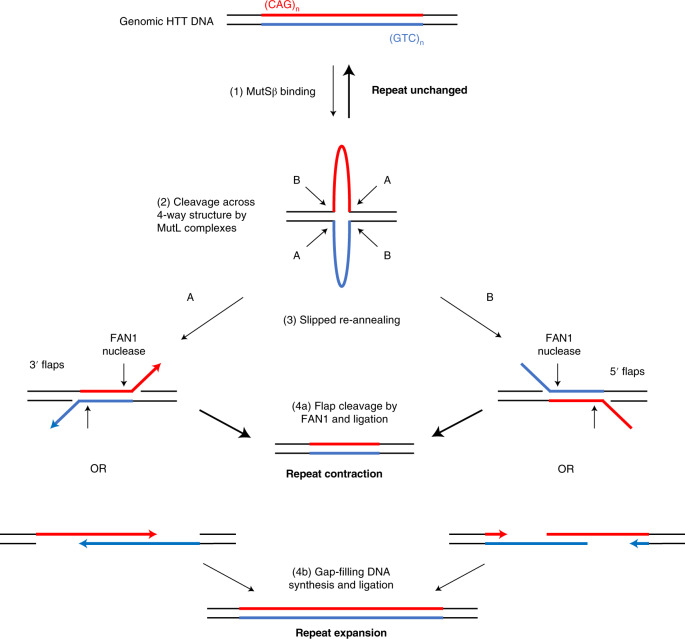
Model of how FAN1 nuclease activity might prevent repeat expansions. The fully base-paired (CAG)•(CTG) tract is in dynamic equilibrium with a four-way junction that includes loop-outs of (CAG)_n_ and (CTG)_n_ on their respective strands. Under normal cellular conditions, most repeats are in their native double-stranded conformation. However, when a longer repeat tract (>35 CAG) is present, it can adopt a more stable four-way structure that can be further bound and stabilized by MSH2/MSH3 (MutSβ) (1). This four-way junction can be cleaved on both strands in either of two orientations (A or B) by MutL complexes (2). The resulting DNA products have long overhangs, either 3′ (A) or 5′ (B), and they can either anneal fully to re-form the starting genomic DNA with no change in repeat tract length (top) or they can slip before partial reannealing (3). Slipped products can have 5′ or 3′ flaps, and these are a substrate for FAN1 nuclease cleavage (bold arrows) and subsequent ligation, yielding repeat contractions (4a). Alternatively, the slipped products can have gaps, and these are substrates for gap-filling DNA polymerases, with subsequent ligation yielding repeat expansions (4b).

The substrate preference of FAN1 nuclease is short DNA flaps^11,34,43^. We propose that FAN1 mediates its anti-expansion effects through cleavage of such flaps in expansion intermediates (Fig. 6). Overall, this would shift the equilibrium between repeat expansion and contraction at an expanded CAG repeat toward the latter, helping to maintain or reduce repeat lengths. This model predicts that factors favoring repeat expansions, such as MSH3, MLH1 and an expansion-prone *HTT* CAG repeat, would be epistatic to FAN1 in determining repeat stability, consistent with our non-canonical repeat observations (Fig. 2) and recent data from HD mouse models^20^. FAN1 nuclease likely has a similar anti-expansion, disease-modifying function in other repeat expansion disorders. For example, *FAN1* variants are associated with altered age at onset of CAG expansion-related spinocerebellar ataxias^47^, and Fan1 inhibits somatic expansion in mouse models of Fragile X disorders^48^.

In conclusion, the genetic architecture of the onset modifier trait in HD is similar to that of other oligo-genic or poly-genic diseases or traits, consisting of both common variants of small effect and rarer variants of larger effect^49^. Our study population was solely of European origin. Rare variant studies in other populations could prove valuable and reveal new genetic modifier signals, as demonstrated for common variants in a Venezuelan HD cohort^50^. Our finding that single, heterozygous coding variants in modifier genes can be associated with clinically relevant changes in HD onset or severity suggests that drugs targeting individual modifiers, or their regulators, could be effective. Such therapeutics are already in development.

## Methods

### Clinical sample selection and CAG length determination

Human subjects were selected from two HD patient cohorts of European origin. The first cohort was from the European REGISTRY-HD study^53^, which primarily enrolled individuals with HD and clinical onset. In total, 6,086 individuals had both a known inherited pure CAG length (40–55 CAGs; after sequencing in this project, the range was 38–55 CAGs) and an age at onset of motor symptoms. Of these, 3,046 had CAG lengths determined in line with REGISTRY protocols (https://www.enroll-hd.org/enrollhd_documents/2016-10-R1/REGISTRY-protocol-3.0.pdf), and 3,040 had CAG lengths determined by local laboratories. CAG lengths on which samples were selected were determined by a standard PCR fragment length assay and assumed a canonical glutamine-encoding repeat sequence in *HTT*. The residual age at motor onset was calculated for each participant by subtracting the expected age at onset given pure CAG length from the observed age at motor onset^8,10^. Expected age at onset was estimated using pure CAG length based on the Langbehn equation that was derived for 41–55 CAGs^26^. For 38, 39 and 40 CAGs, we used 65, 62 and 59 years, respectively, extrapolating from the Langbehn model. Observed age at motor onset was determined as follows: where onset was classified as motor, oculomotor or mixed, the rating clinician’s best estimate for motor onset was used (sxrater). For non-motor onsets, or where the clinician’s onset estimate was missing, the motor component of the clinical characteristics questionnaire was used instead (ccmtrage)^1^. The REGISTRY-HD population was stratified by residual age at onset and 250 (~4%) at each extreme of the distribution selected for analysis (exact samples depending on DNA availability), along with seven technical exome-sequencing controls of different CAG repeat lengths and residuals of less than 1 year. After targeted *HTT* CAG repeat sequencing using MiSeq (Fig. 2), we determined accurate residual ages at motor onset for the REGISTRY-HD population using sequenced pure CAG lengths. These corrected residuals were used in all subsequent analyses.

The second cohort (*n* = 238) was from the global (mainly US) PREDICT-HD study (total *n* = 1,069) that enrolled healthy at-risk *HTT* mutation carriers and prospectively followed them to clinical onset over 10 years^27^. Phenotypic extremes were selected in two ways. First, extremes of motor and cognitive phenotype were chosen, using Total Motor Score (TMS) and Symbol Digit Modalities Test (SDMT) score, respectively. Extremes were required to persist across the first and third visits for the participants to help ensure consistency. Locally weighted scatterplot smoothing (LOESS)^54^ was used to predict each variable using CAG, age at study entry and their interaction, with the predictors as a group being an index of HD progression^55^. LOESS residuals were computed, which reflected an individual’s deviation from the value that was expected based on their CAG/age combination. For each visit, individuals were ranked by the residuals, and those who were consistently in the top or bottom 20% were selected as ‘consistent phenotypic extremes’ for the exome analysis (*n* = 117, TMS group; *n* = 85, SDMT group). Second, we selected a cohort with predicted early-onset or predicted late-onset HD. For those individuals who phenoconverted to Diagnostic Confidence Level 4 (DCL4) during the study, the age at DCL4 was available as a proxy for age at onset. Using the PREDICT-HD database, extremes of expected onset were selected based on the relationship between the CAG Age Product (CAP)^55^ and 10-year survival from motor diagnosis (DCL = 4). CAP was computed as CAP = age at study entry × (CAG − 33.66), and the predicted 10-year survival was the restricted mean survival time (RMST) computed for each participant. RMST was based on the random survival forest (RSF)^56^ using progression in clinical variables to predict time to motor diagnosis. For each participant with two or more time points, the intercept and slope over time of TMS, SDMT, Stroop interference and total functional capacity were estimated using ordinary least squares. The intercepts and slopes were then used as predictors of time to DCL = 4 in the RSF. The RMST was computed by estimating the survival curve for each individual based on their predictor profile and then taking the area under the curve up to 10 years. RMST is the ‘survival expectancy’ (non-diagnosis expectancy) when we follow-up a cohort from study entry to 10 years^57^. The participants were then subdivided into 20 groups of 53–54 by CAP score and the mean RMST determined for each group. For each participant, the absolute difference between the individual RMST and the RMST of their group was calculated. Among the ~6% extremes, 63 relatively earlier RMST and 56 relatively later RMST had DNA available and were included in whole-exome sequencing (*n* = 119 total).

Pure CAG repeat lengths in expanded *HTT* alleles were initially determined by standard PCR fragment length analysis in PREDICT-HD. To determine glutamine-encoding *HTT* repeat tract sequences, whole-exome sequencing reads were extracted and manually assessed. Although it was not possible to determine full allelic structures from read lengths of 75 base pairs (bp), phase could be determined for non-canonical alleles, as unexpanded CAG lengths were short enough to be effectively captured (Supplementary Table 2). Using this information, pure CAG repeat lengths were adjusted and used in all analyses.

### Ethical approvals

Ethical approval for REGISTRY-HD was obtained in each participating country. Investigation of de-identified PREDICT-HD participants was approved by the institutional review board of Partners HealthCare (now Mass General Brigham). Participants from both studies gave written informed consent. All experiments described here were conducted in accordance with the Declaration of Helsinki. Local ethical approval was through Cardiff University School of Medicine (SMREC 19/55).

### Sequencing of *HTT* exon 1 repeat locus using MiSeq

We used a MiSeq-based *HTT* sizing protocol as described elsewhere^8,12,58^. In brief, DNA from low-passage lymphoblastoid cell lines was diluted to 4 ng µl^−1^ using PicoGreen (Thermo Fisher Scientific). Libraries were prepared in 384-plate format using MiSeq-compatible primers as described^58^. PCR reactions used LA Taq polymerase in GC Buffer II (Takara). Library clean-up used two AMPure XP SPRI bead (Beckman Coulter, A63881) steps, the first at 0.6× and the second at 1.4× bead concentrations. Libraries were checked using a Bioanalyzer (Agilent). Libraries were sequenced using a 600-cycle MiSeq version 3 reagent kit (Illumina) running with 400-bp forward and 200-bp reverse sequencing.

### *HTT* allele structure determination

MiSeq data were analyzed using the Scale-HD pipeline (version 0.322; https://github.com/helloabunai/ScaleHD)^12^. Aligned sequences were also manually checked using Tablet^59^ in case rare alleles without known RefSeq were identified, which occurred in four instances on three alleles: (CAG)_n_**CAC**(CAG)_3_**CAA**CAG, (CAG)_n_(**CAA**)_2_CAG and (CAG)_n_(**CAA**)_3_CAG. Two REGISTRY-HD samples failed MiSeq sequencing; for these samples, and for all PREDICT-HD samples, reads were extracted from whole-exome data using SAMtools^60^ using region 4:3073000–308000. Reads were manually assessed for presence of atypically interrupted *HTT* alleles and phased by comparison with non-expanded allele sequences. A summary of *HTT* repeat sequences for the PREDICT-HD cohort is in Supplementary Table 2.

### Whole-exome sequencing

A formal power calculation for exome sequencing of 80 samples taken from the extremes of the REGISTRY-HD population, based on residual age at onset, gave 88% power to detect individual variants of similar effect size to those reported in GeM-HD GWAS at a significance level of 5%. Ultimately, we used a much larger sample size as more resources became available. For the REGISTRY-HD cohort (*n* = 507), sequencing was performed at Cardiff University. Patient DNA derived from low-passage lymphoblastoid cells was obtained from the European HD Network (EHDN, projects 0791 and 0803; DNA prepared by BioRep). Whole-exome libraries were generated using TruSeq rapid exome library kits (Illumina). Libraries were pooled in equimolar amounts in groups of 96 and run over eight lanes on a HiSeq 4000 patterned flow cell. Clustering used Illumina ExAmp reagents from a HiSeq 3000/4000 PE cluster kit (Illumina) on a cBot system. Sequencing used a 2 × 75 bp end run with a HiSeq 3000/4000 SBS kit for 150 cycles (Illumina). Note that the exome sequencing data include significant coverage of promoter and untranslated regions due to the use of the TruSeq rapid exome library kit (Illumina).

For the PREDICT-HD cohort, an in-solution DNA probe based hybrid selection method was used to generate Illumina exome sequencing libraries from blood DNA^61,62^. The exome library specifically targets approximately 37.7 Mb of mainly exonic territory made up of all targets from Agilent SureSelect All Exon V2, all coding regions of GenCode V11 genes and all coding regions of RefSeq gene and KnownGene tracks from the UCSC Genome Browser (http://genome.ucsc.edu). Pooled libraries were normalized to 2 nM, and flow cell cluster amplification and sequencing were performed according to the manufacturer’s protocols using the HiSeq 2500. Each run was a 75-bp paired end with a dual eight-base index barcode read. Data were analyzed using the Broad Picard Pipeline, which includes de-multiplexing and data aggregation.

### Sanger sequencing

All FAN1 variants identified by exome sequencing were confirmed by Sanger sequencing in the REGISTRY-HD sample. Amplifications for Sanger sequencing were performed using MyTaq (Bioline) using ~20–60 ng of template DNA, and reactions were cleaned up with QIAquick PCR purification columns (Qiagen). Then, 1.5 μl of the relevant sequencing primer (25 μM) was added to each purified sample (Supplementary Table 9), and sequencing was performed using the Eurofins Genomics LIGHTRUN service.

### FAN1 structural modeling

FAN1 structure annotation (Fig. 3d) used FAN1 in complex with DNA^63^. This structure (Protein Data Bank ID: 4RI8) contained residues 370–1,017. Two small loop sections (510–518 and 799–810) were missing and added to the model using the homology model tools within the Molecular Operating Environment.

### Lymphoblastoid cell line culture and mitomycin C survival assay

Lymphoblastoid cell lines from individuals with HD were obtained from EHDN (projects 0791 and 0803). Lines were cultured in RPMI-1640 GlutaMAX (Thermo Fisher Scientific) supplemented with 15% FBS and 1% penicillin–streptomycin and passaged three times per week. For the mitomycin C survival assays, cell lines (viability > 80%) were seeded in triplicate at 20,000 cells per well in 12-well plates, treated once with mitomycin C (0–150 nM, SelleckChem) and cultured for a further 7 days before viable cell counts were determined by trypan blue staining (Countess II, Thermo Fisher Scientific). Each experiment was independently repeated three times. Difference in means was tested using Student’s *t*-test (two-tailed). Data distribution was assumed to be normal, but this was not formally tested.

### Protein purification and nuclease assays

Full-length Nus-His-FAN1 proteins were expressed in *E. coli* and partially purified in one step using cobalt agarose^11^. Further purification of FAN1 proteins was attempted, but active protein yields were low. Proteins were stored in 50 mM Tris-Cl pH 7.5, 270 mM sucrose,150 mM NaCl, 0.1 mM EGTA, 0.03% Brij-35 and 0.1% β-mercaptoethanol.

Double-stranded DNA substrate containing a 5′ single-strand overhang with an IR-700 fluorescent label (5′ flap DNA) was generated by annealing three oligonucleotides (TOM112, TOM117 and TOM122, based on published sequences^64^) at 95 °C for 10 minutes and cooling slowly to room temperature overnight. Nuclease assays were carried out by pre-incubating 10 nM Nus-His-FAN1 protein, normalized using FAN1 band intensities from SDS-PAGE (Supplementary Fig. 3b), with 5 nM 5′ flap DNA in 9 μl of reaction buffer (25 mM Tris-Cl pH 7.4, 25 mM NaCl, 0.1 mg ml^−1^ of BSA and 0.1 mM DTT) for 5 minutes on ice and then adding 1 μl of MnCl_2_ (10 mM) and incubating at 37 °C to start reactions. Reactions were continued for 4 minutes before quenching with 10 μl of stop buffer (1× TBE containing 12% Ficoll Type 400, 7 M urea, orange loading dye (LI-COR Biosciences) and 4 mM EDTA). Samples were denatured at 95 °C for 5 minutes and run on a 15% TBE urea gel (200 V, 1 hour) before imaging and quantitation on an Odyssey CLx (LI-COR Biosciences).

### HD iPSC model

The human iPSC lines CS09iHD109-n1, CS09iHD109-n4 and CS09iHD109-n5 (herein referred to as Q109-n1, Q109-n4 and Q109-n5) are independent lines generated previously from a human HD fibroblast line, ND39258 (RRID:CVCL_ZC78), with an expanded *HTT* allele initially containing 109 pure CAG trinucleotides^33^. Lines were the HD iPSC consortium that originally derived the lines (CHDI). *HTT* genotypes were confirmed by sequencing, and lines were checked by exome sequencing and copy number variation (CNV) analysis. The expanded and wild-type *HTT* CAG repeats of the Q109 cells both have a canonical single CAACAG after the pure CAG tract. SNP array genotyping (virtual karyotyping) of iPSC lines was carried out in-house at Cardiff University. Genomic DNA was extracted using QIAamp DNA Mini Kit (Qiagen), and 200 ng (50 ng µl^−1^) was used for genotyping. Samples were genotyped on the Infinium PsychArray-24 Kit (Illumina) or the Infinium Global Screening Array-24 (Illumina) and scanned using the iScan System (Illumina). Data were exported from Genome Studio and analyzed using PennCNV^65^. Sample-level quality control was applied based on the standard deviation of log R ratio set at 0.3, minimum SNP number of 10 and minimum region size of 100,000 bp.

### HD iPSC culture and differentiation

iPSCs were cultured on Geltrex-coated plates (37 °C, 1 hour) (Life Technologies) in Essential 8 Flex medium (Life Technologies) under standard culturing conditions (37 °C, 5% CO_2_). iPSCs were passaged every 3–4 days using ReLeSR (STEMCELL Technologies) and seeded at a split ratio of 1:12. Full media changes were performed every 1–2 days. For differentiations, iPSC colonies were dissociated into a single-cell suspension using Accutase (Life Technologies), seeded into 12-well plates coated with Growth Factor Reduced Matrigel (0.5 ng ml^−1^, BD Biosciences) and cultured in iPSC medium until the cells reached ~80% confluency. iPSCs were differentiated to forebrain neurons using adaptations of published protocols^66,67^, as follows. iPSCs were induced into NPCs using Advanced DMEM/F-12 (ADF, Life Technologies) supplemented with 1% GlutaMAX (Thermo Fisher Scientific), 1% penicillin–streptomycin (5,000 U/5,000 μg) (Gibco), 2% MACS neurobrew without retinoic acid (Miltenyi), 10 µM SB431542 (Miltenyi), 1 µM LDN-193189 (Stemgent) and 1.5 µM IWR-1-endo (Miltenyi) up until day 8, upon which SB was omitted from the medium and 25 ng ml^−1^ of Activin A (PeproTech) was added. Full media changes were performed daily up until day 16. Day 16 NPCs were passaged into plates coated with poly-d-lysine (Thermo Fisher Scientific) and Growth Factor Reduced Matrigel. Cells were fed with SJA medium consisting of ADF with 1% GlutaMAX, 1% penicillin–streptomycin, 2% MACS neurobrew with retinoic acid, 2 μM PDO332991 (Bio-Techne), 10 μM DAPT (Bio-Techne), 10 ng ml^−1^ of BDNF (Miltenyi), 0.5 μM LM22A4 (Bio-Techne) and 10 μM Forskolin (Bio-Techne), 3 μM CHIR 99021 (Bio-Techne), 0.3 mM GABA, 1.8 mM CaCl_2_ (Sigma-Aldrich) and 0.2 mM ascorbic acid. After 7 days in SJA medium, cells were fed with SJB medium consisting of equal amounts of ADF and Neurobasal-A (Life Technologies) with 1% GlutaMAX, 1% penicillin–streptomycin, 2% MACS neurobrew with retinoic acid, 2 μM PDO332991, 10 ng ml^−1^ of BDNF, 3 μM CHIR 99021, 1.8 mM CaCl_2_ and 0.2 mM ascorbic acid. After 14 days in SJB medium, cells received half media changes every 3–4 days with BrainPhys Neuronal Medium (STEMCELL Technologies) supplemented with 1% penicillin–streptomycin, 2% MACS neurobrew with Vitamin A and 10 ng ml^−1^ of BDNF.

### Generation of edited iPSC lines using CRISPR–Cas9 (see also Extended Data Fig. 2)

*FAN1* knockout: Two guide RNAs (gRNAs) targeting exon 2 of FAN1; 5′-CTGATTGATAAGCTTCTACG**AGG**-3′ and 5′-GCACCATTTTACTGCAAACG**GGG**-3′ were designed on DESKGEN cloud (www.deskgen.com) to produce a 94-bp deletion. crRNA and tracrRNA-ATTO-550 (IDT) were combined in nuclease-free duplex buffer (IDT), annealed (95 °C, 2 minutes), combined with Cas9 (IDT) and incubated (room temperature, 20 minutes) to form a ribonucleoprotein (RNP). iPSCs were nucleofected with both RNPs using the 4D-Nucleofector and P3 Primary Cell 4D-Nucleofector X Kit and program CA137 (Lonza). After 24 hours, iPSCs were sorted on the FACSAria Fusion to obtain the top 10% of cells, which were plated as single cells. After 7 days, individual colonies were manually dislodged and plated into single wells of a 96-well plate, which, after 7 days, were passaged into replicate plates using Gentle Cell Dissociation Reagent (STEMCELL Technologies). For screening, DNA was extracted using QuickExtract (Cambio) (room temperature, 10 minutes; 65 °C, 6 minutes; 95 °C, 2 minutes) and PCR amplified using two primer pairs amplifying exon 2 of FAN1; FAN-KO, 5′-CCTGTGTTTTATTGCTCAGAACA-3′ and 5′-CATTTCATCAAGGTGCCGGT-3′ and FAN1-T7, 5′-TCAGAGTTCGCTTTTCCCCT-3′ and 5′-GATGCTAGGCTTCCCAAACA-3′. Amplicons were visualized on a 1.5% agarose gel on the Gel Doc XR system (Bio-Rad). Sanger sequencing was used to confirm successful editing.

FAN1 D960A variant. D960A was introduced by using cellular homology-directed repair (HDR). A single gRNA sequence (5′-AGGGGGCCTCCCCGACCTGG**TGG**-3′) and a 122-bp repair template containing the desired edit (5′- AGGaGGCCTCCCCG**c**CCTGGTcG-3′) provided a template for HDR. The HDR template contained two silent mutations (5′- AGG**a**GGCCTCCCCGcCCTGGT**c**G-3′), one of which was in the PAM sequence to prevent further excision, and introduced restriction sites enabling efficient screening. Nucleofection was carried out as above. For screening, DNA was extracted using QuickExtract and PCR amplified using two primer pairs: FauI-D960A, 5′-TCACGAGGGAAGTGGCTAAC-3′ and 5′-GCCAACAGCCACTCAAGAAATG-3′ and StuI-D960A, 5′-TCACGAGGGAAGTGGCTAAC-3′ and 5′- CACAGAATACAGCAGGAGTGATG-3′. Restriction digest was performed with FauI or StuI in CutSmart Buffer (NEB). Amplicons were separated on a 1.5% agarose gel and visualized on the Gel Doc XR system (Bio-Rad). Sanger sequencing was used to confirm successful editing.

### PCR-based fragment size analysis of *HTT* exon 1 repeat locus in Q109 iPSCs

DNA was extracted using the QIAamp DNA Mini Kit (Qiagen). A fluorescently labeled forward primer FAM huHTT 1F: 5′-6-FAM-ATGAAGGCCTTCGAGTCCCTCAAGTCCTTC-3′ and a reverse primer: 5′-GGCGGCTGAGGAAGCTGAGGA-3′ were used to PCR amplify the CAG repeat region of *HTT*. Cycling conditions were as follows: initial denaturation at 94 °C for 90 seconds, followed by 35 cycles of 94 °C for 30 seconds, 65 °C for 30 seconds and 72 °C for 90 seconds and a final elongation at 72 °C for 10 minutes. Resulting PCR products were sent for sizing with the GeneScan LIZ 600 dye Size Standard (Applied Biosystems) and run on the G3130xL Genetic Analyzer (Applied Biosystems). Files were analyzed using GeneMapper (version 4.1, Applied Biosystems), Fragman (version 1.0.9)^68^ and AutoGenescan (https://github.com/BranduffMcli/AutoGenescan), and quantification was performed with a 10% peak height threshold applied^69^.

### Cell immunocytochemistry

iPSCs and neurons were fixed in 4% paraformaldehyde for 15 minutes at room temperature. Cells were permeabilized in 0.1% Triton-X in PBS for 20 minutes at room temperature and blocked in 3% BSA with 3% goat serum and 0.1% Triton-X. Primary antibodies used were OCT4 (Abcam, ab19857, 1:100), CTIP2 (Abcam, ab18465, 1:200) and MAP2 (Abcam, ab32454, 1:500). Alexa Fluor goat anti-mouse 488 (Invitrogen, A11001, 1:400) and Alexa Fluor goat-anti rabbit 568 (Invitrogen, A11011, 1:800) were used as secondary antibodies.

### Immunoblotting

Cells were washed once in DPBS and lysed with RIPA buffer (Sigma-Aldrich) containing cOmplete, EDTA-free Protease Inhibitor Cocktail Tablets (Merck). Protein samples were denatured at 70 °C for 10 minutes in 4× NuPAGE LDS Sample Buffer (Life Technologies). Next, 40 µg of cell protein extract per sample was separated on NuPAGE 4–12% Bis-Tris gradient gels (Life Technologies) alongside PageRuler Plus Prestained Protein Ladder (Thermo Fisher Scientific) using MOPS SDS NuPAGE Running Buffer (Invitrogen) at 200 V for 50 minutes. Where purified proteins were run, 30 ng of protein per lane was loaded. Gels were then transferred to methanol-activated Immobilon-P PVDF membranes (Sigma-Aldrich) using NuPAGE Transfer Buffer (Invitrogen) at 120 V for 45 minutes. The membrane was blocked in 5% milk in PBS-T and incubated overnight at 4 °C with anti-FAN1 (CHDI, sheep polyclonal, 1:1,000) and anti-β-tubulin (UpState, 05-661, mouse monoclonal, 1:10,000). Donkey anti-mouse IgG Alexa Fluor 680 (Invitrogen, A32788, 1:10,000) and IRDye 800CW donkey anti-goat IgG secondary antibody (LI-COR Biosciences, 926-32214, 1:15,000) were used as secondary antibodies. Immunoblots were visualized with the Odyssey CLx Imaging System using β-tubulin as a loading control.

### Reagents

The sequences of all oligonucleotides used in this project are provided in Supplementary Table 9. The names and catalog numbers (where appropriate) of all reagents used in this project are provided in Supplementary Table 10.

### Quantification and statistical analyses

#### Exome sequencing: alignment and variant calling

We used a standard Genome Analysis Toolkit (GATK, version 3) best practices workflow for the alignment and variant calling of both sets of exomes^70–72^. Reads were de-multiplexed, and adapters were soft clipped using Picard (https://github.com/broadinstitute/picard). Alignment used BWA-MEM^73^ to the hg19/GRCh37 genome assembly. Local insertion/deletion realignment used GATK, and duplicate reads were marked and reads aggregated across lanes with Picard. Base quality scores were recalibrated using GATK’s base quality score recalibration (BQSR). Germline SNPs were called with GATK’s haplotype caller^74^. Variant quality score recalibration (VQSR) was performed on both SNPs and insertion/deletion variants using GATK’s recommended parameters for exome sequencing.

#### Exome sequencing: quality control and annotation

Whole-exome data were subject to a multi-step quality control pipeline (Extended Data Fig. 1a). Picard’s CollectHsMetrics function was used to assess target exome coverage; to be included in the study, exomes required ≥70% of the exome covered at 10× or greater. Per-sample mean genotype quality, mean depth and call rate were then determined using Hail (https://github.com/hail-is/hail). Exomes more than 3 standard deviations smaller than the mean of any of the three metrics were excluded for REGISTRY-HD exomes. VerifyBamID^75^ was used to detect contamination, and six samples with a Freemix >0.075 were excluded, as per the ExAC study^76^. Where there were duplicate samples, the exome with the highest coverage was retained. Sex imputation used Peddy^77^; samples with conflicting imputed and recorded sex were excluded. One individual with originally unknown sex was kept. Ancestry was estimated using Peddy by principal component analysis (PCA) against genomes from the 1000 Genomes Project phase 3 (ref. ^78^). Samples were excluded if they were either (1) predicted to have non-European ancestries by Peddy or (2) outside the primary cluster of European samples in Supplementary Fig. 1. We focused on cohorts of European origin because HD is more common in these populations, and most of the large longitudinal studies, such as REGISTRY-HD and PREDICT-HD, have insufficient numbers of individuals of non-European ancestries to power rare variant association analyses. First-degree relatives were identified using Hail’s genetic relatedness matrix function, with a cutoff of 0.125. For each pair of related individuals, the individual with the most extreme uncorrected residual age at motor onset was retained.

Exomes underwent a multi-step annotation pipeline (Extended Data Fig. 1b). Exomes were annotated by gnomAD version 2.1.1 (ref. ^42^), dbNSFP version 4.0b2 (refs. ^79,80^) and Variant Effect Predictor (VEP) version 95 (ref. ^81^). Homozygotes were defined as having ≥90% of reads as either the reference or the alternative allele, whereas heterozygotes were defined as having between 25% and 75% of the reference or alternative allele. Loss-of-function calls were defined as ‘HIGH’-impact calls by VEP, and non-synonymous, damaging calls were defined as either ‘HIGH’ or ‘MODERATE’ calls. Non-synonymous, damaging calls included loss-of-function calls or non-synonymous calls with ≥20 CADD score. Hail was used for PCA. Baseline variant rates were determined for each exome as the total number of variant classes at various MAFs.

In total, 683 exomes passed quality control. After annotation, we identified 311,960 high-quality (≥75% call rate and ≥98.50 VQSR) non-reference variants (Supplementary Fig. 2), with a mean of 35,808 variants per individual. There were 150,139 different non-synonymous variants (moderate- and high-impact variants) in our cohort with a mean of 12,700 such variants per individual, similar to previously reported population frequencies (Supplementary Fig. 2)^76^. Two groups of exomes were created for downstream analyses (Extended Data Fig. 1). First, a dichotomous group divided into extreme phenotypes (*n* = 637; 421 REGISTRY-HD and 216 PREDICT-HD). Early/more severe phenotype (*n* = 315): individuals with early onset relative to pure CAG length (age at motor onset > 5 years earlier than expected, based on sequenced CAG lengths in REGISTRY-HD) or predicted early onset and/or worst 5% in TMS/SDMT (PREDICT-HD). Later/less severe phenotype (*n* = 322): individuals with late onset relative to pure CAG length (age at motor onset > 5 years later than expected, based on sequenced CAG lengths in REGISTRY-HD) or predicted late onset and/or best 5% in TMS/SDMT (PREDICT-HD). Second, a smaller continuous phenotype group containing all those with known age at motor onset and *HTT* repeat sequence and, hence, a calculable and accurate age at motor onset residual that could be used as a quantitative trait (*n* = 558; 463 REGISTRY-HD and 95 PREDICT-HD).

#### Exome sequencing: association analyses of rare variation

Coding variants that modify phenotype are likely to be deleterious to protein function. Therefore, our primary association analyses used variants meeting either of the following two criteria: (1) loss-of-function variants (frameshifts, start/stop lost, premature stop codons and splice donor/acceptor variants) or (2) non-synonymous variants with a CADD-PHRED score ≥ 20 (that is, the 1% predicted most damaging in the human genome)^82^. Additionally, variants were required to have MAF < 1%, as defined by the European cohort of gnomAD (version 2.1.1)^42^. To be included, variants required a call rate ≥ 75% and VQSR ≥ 98.50. Secondary analyses were performed on loss-of-function and all non-synonymous variants, separately.

Given that coding variants of interest are individually rare, we collapsed qualifying coding variants on genes in the exome and tested each gene with at least ten variants (*n* = 3,912 genes (dichotomous group); *n* = 3,198 genes (continuous group)) for association with residual HD onset using the Optimal Sequence Kernel Association Test (SKAT-O)^83,84^. SKAT-O combines elements of both a burden test and a Sequence Kernel Association Test (SKAT) and, therefore, does not assume that all variants in a gene have the same direction of effect. The dichotomous group (*n* = 637) was analyzed using logistic regression, with late/less severe phenotype coded as 0 and early/more severe phenotype coded as 1. The continuous group (*n* = 558) was analyzed using linear regression. We included as covariates population principal components 1–5 (to correct for population stratification), the baseline variant rate (number of variants per variant class examined), mean sample depth and study group (REGISTRY-HD or PREDICT-HD). Additionally, the presence or absence of non-canonical *HTT* repeats in the expanded allele was a covariate in logistic analyses. Baseline variant rate was calculated for each individual and represented the total number of variants observed in the exome that passed quality control at the particular MAF/damaging filter being used. Multiple testing correction was performed using Bonferroni correction for the number of genes tested in each analysis. A burden test was additionally run using the same cutoffs and covariates as SKAT-O on the logistic patient group (*n* = 637) using a Wald logistic burden regression test, implemented in Hail.

Note that SKAT-O analysis without correcting for non-canonical *HTT* repeat sequences and accurate CAG repeat lengths from sequencing found one exome-wide significant signal in *NOP14* on chromosome 4, 130 kbp upstream of *HTT* (*P* = 8.3 × 10^−6^, continuous analysis). We found that NOP14 R697C (4:2943419:G:A) is in strong linkage disequilibrium with the pathogenic (CAG)_n_(CAACAG)_2_ *HTT* allele (Fig. 2, allele group e, *R*^2^ = 0.902), explaining the association of *NOP14* variation with HD phenotype. Correction for *HTT* repeat sequence and accurate CAG length ablated this signal.

We also tested whether the association of rare variation with onset observed in FAN1 was independent of the previous GWAS results in two ways (Supplementary Table 3). First, we ran the SKAT-O analysis on the logistic patient group (*n* = 637) after removing the R377W and R507H variants that were reported as being associated with earlier onset in the GWAS. Second, we tested association of the burden of rare, damaging variation on each of the four lead variants identified by GWAS^30^ by logistic regression. This analysis was performed in the 441 individuals with both exome sequencing and GWAS data. The burden of rare, damaging variation was defined both including and excluding R377W and R507H.

#### Statistical modeling and analysis of iPSC data

Data collection and analysis were not performed blinded to the conditions of the experiments. No data points were excluded from analyses. The primary outcome measure was change in modal CAG from its initial value. Secondary analyses looked at changes in expansion and instability index. All outcome measures are zero when time is zero, requiring regression models without intercepts to be fitted. These are detailed below.

D960-D42 iPSC data: data consisted of three wild-type, three FAN1^WT/D960A^ heterozygous, three FAN1^D960A/D960A^ clonal lines and two FAN1^−/−^ knockout lines. Each clonal line was cultured in triplicate wells that remained independent from one another for the duration of the experiment and were repeatedly measured at different time points. Observations are, therefore, correlated if they are taken from the same line and/or well, and it is important that statistical analyses take these correlations into account. This was done by performing mixed effects linear regression using the lmer() function in R, fitting random effects for the variation in rate of change of outcome between lines and wells. The models fitted were:

$${{{\mathrm{m}}}}0 <\!\! -\; {{{\mathrm{lmer}}}}\!\left( {{{{\mathrm{change}}}}\sim\!\! 0 + {{{\mathrm{time}}}} + \left( {0\!\! +\!\! {{{\mathrm{time}}}}|{{{\mathrm{line}}}}} \right) + (0\!\! +\!\! {{{\mathrm{time}}}}|{{{\mathrm{well}}}})} \right)$$

$${{{\mathrm{m}}}}1 <\!\! - {{{\mathrm{lmer}}}}({{{\mathrm{change}}}}\sim\!\! 0 + {{{\mathrm{time}}}} + {{{\mathrm{time}}}}\!\!:\!\!{{{\mathrm{geno}}}} + \left( {0 \!\!+\!\! {{{\mathrm{time}}}}|{{{\mathrm{line}}}}} \right) + (0\!\! +\!\! {{{\mathrm{time}}}}|{{{\mathrm{well}}}}))$$

The significance of different genotypes on rate of change of outcome is calculated by anova(m1,m0).

Genotype was initially coded as a four-level factor: 1 = WT/WT, 2 = 960A/WT, 3 = 960A/960A and 4 = −/−, giving a 3-df test. Post hoc analyses on the pattern of genotype differences were performed by fitting models with restrictions on the genotype effects and comparing (via ANOVA) to the general 3-df model. Estimates of expansion rates for each genotype were produced by the R command m2 < - lstrends(m1, ~geno, var = ‘time’), and post hoc pairwise comparisons of genotype effects using the Tukey method were obtained by the command pairs(m2).

FAN1 knockout (iPSC data): Data consisted of seven wild-type (*FAN1*^+/+^) lines and six *FAN1* knockout (*FAN1*^−*/*−^) lines in this experiment. Three of the *FAN1*^*+/+*^ lines and two of the *FAN1*^−*/*−^ lines were cultured in triplicate wells, the remainder in single wells. Wells remained independent from one another for the duration of the experiment and were repeatedly measured at different time points. Effect of genotype on the rate of change of the outcome phenotype was analyzed using the same regression models that were used for the D960A-D42 iPSC experiment.

*FAN1* knockout (neuronal data): Data consisted of five lines of N1-FAN1^+/+^ and four lines of N1-*FAN1*^−/−^. A separate, independent well was taken from each line at each time point. So, observations are correlated only through shared line, not through shared well.

Effect of genotype (+/+ versus −/−) on outcome was tested by fitting the following two zero intercept mixed effect linear models:

$${{{\mathrm{m}}}}0 < \!\!-\; {{{\mathrm{lmer}}}}({{{\mathrm{change}}}}\sim 0 + {{{\mathrm{time}}}} + (0 + {{{\mathrm{time}}}}|{{{\mathrm{line}}}}))$$

$${{{\mathrm{m}}}}1 <\!\! -\; {{{\mathrm{lmer}}}}({{{\mathrm{change}}}}\sim 0 + {{{\mathrm{time}}}} + {{{\mathrm{time}}}}:{{{\mathrm{geno}}}} + (0 + {{{\mathrm{time}}}}|{{{\mathrm{line}}}}))$$

Again, the effect of genotype on the rate of change of the measures is tested by anova(m0,m1).

D960A neural precursor data: Data consist of one line each of WT/WT, D960A/WT and D960A/D960A. A separate, independent well was taken from each line at each time point. So, observations are correlated only through shared line, not through shared well. Effect of genotype on rate of change of outcome was tested by fitting the same zero intercept mixed effect linear models as were used to analyze the *FAN1* knockout neuronal data.

### Reporting Summary

Further information on research design is available in the Nature Research Reporting Summary linked to this article.

## Online content

Any methods, additional references, Nature Research reporting summaries, source data, extended data, supplementary information, acknowledgements, peer review information; details of author contributions and competing interests; and statements of data and code availability are available at 10.1038/s41593-022-01033-5.

## Supplementary information

Supplementary Information Supplementary Figs. 1–4, Supplementary Tables 1–10, Supplementary References, Source Data for Supplementary Figures and Members of Consortia

Reporting Summary

## Data Availability

Phenotypic data, variant call files and MiSeq data are available from the European Genome-phenome Archive (EGA). BAM files of exome sequencing data are available from EGA (REGISTRY-HD) or dbGaP (PREDICT-HD; https://www.ncbi.nlm.nih.gov/gap/, accession number phs000371.v2.p1). Access to the EGA datasets will be provided on reasonable request through a data access committee coordinated by the corresponding authors. Biological materials derived in this work (edited iPSC lines and FAN1 expression plasmids) are available upon reasonable request from the corresponding authors. Source data are provided with this paper.

## Source data

Source Data Fig. 4 Unprocessed gel
Source Data Fig. 5 Unprocessed immunoblot
Source Data Extended Data Fig. 2 Unprocessed gels

## Code availability

Whole-exome sequences were aligned and variants were called using a GATK-based pipeline (https://gatk.broadinstitute.org/hc/en-us), and variant annotation and quality control used Hail version 0.1 (https://hail.is/). SKAT-O analyses were performed in R using the SKAT package (https://github.com/leeshawn/SKAT/), and MiSeq data used ScaleHD (https://scalehd.readthedocs.io/en/latest/). Finally, analyses of GeneScan data used Autogenescan (https://github.com/BranduffMcli/AutoGenescan).
