# Supplementary material for: Exome sequencing of individuals with Huntington’s disease implicates FAN1 nuclease activity in slowing CAG expansion and disease onset
Source: Nat Neurosci. 2022 Apr 4;25(4):446–57. doi: 10.1038/s41593-022-01033-5 (PMC8986535; doi:10.1038/s41593-022-01033-5)
Supplement: Supplementary file 1 — Supplementary Figs. 1–4, Supplementary Tables 1–10, Supplementary References, Source Data for Supplementary Figures and Members of Consortia [file 41593_2022_1033_MOESM1_ESM.pdf]

nature portfolio

https://doi.org/10.1038/s41593-022-01033-5

Supplementary information

Exome sequencing of individuals with Huntington's disease implicates FAN1 nuclease activity in slowing CAG expansion and disease onset

In the format provided by the authors and unedited

Supplementary information

McAllister et al.

Supplementary Figures 1-4

Supplementary Tables 1-10

Supplementary References

Supplementary source data

Members of Consortia

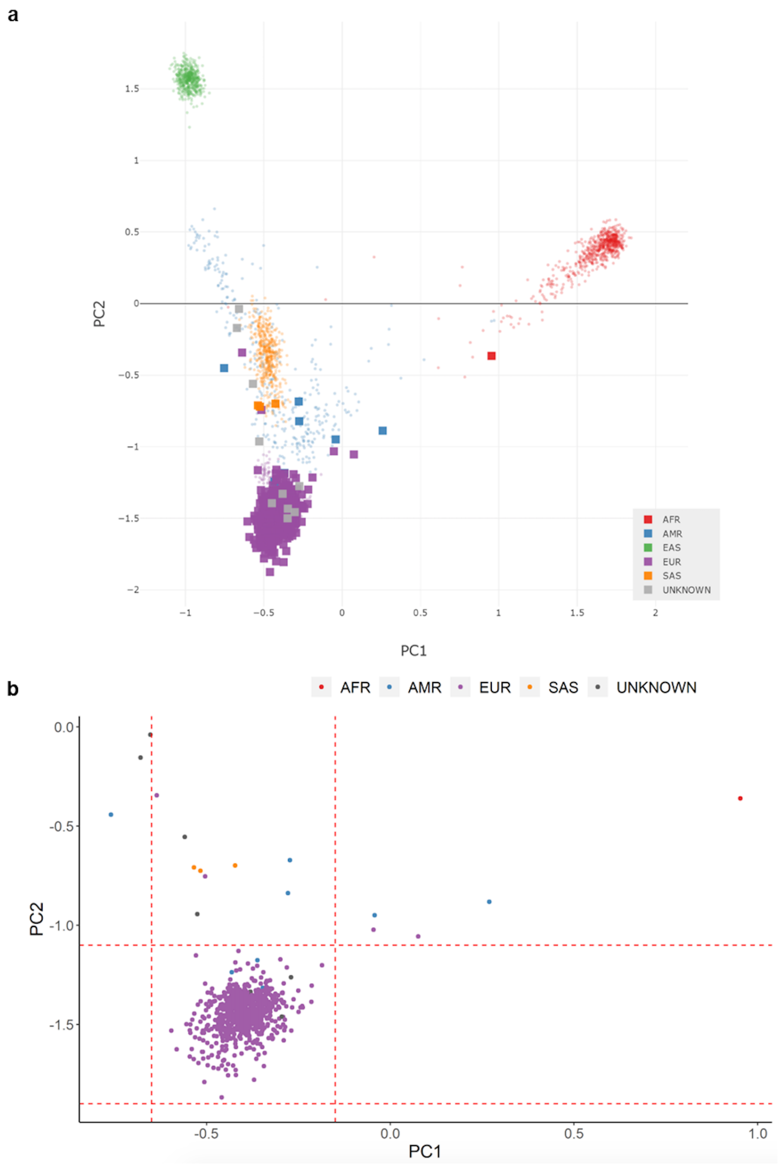

Supplementary Fig. 1: Individuals of European Ancestry were selected for downstream analysis

a, Principal component analysis and estimated ancestries based on the 1,000 genome project as calculated by Peddy[1] for N=725 individuals passing initial quality control (See Fig. S1a). Individuals of European ancestry in purple.

b, Magnified area of the principal component analysis (PC1 and PC2) from (a). Individuals with predicted European ancestry enclosed within the rectangle bordered by dotted lines were retained in analyses (N=700).

Key: AFR, African; AMR, Ad Mixed American; EAS, East Asian; EUR, European; SAS, South Asian

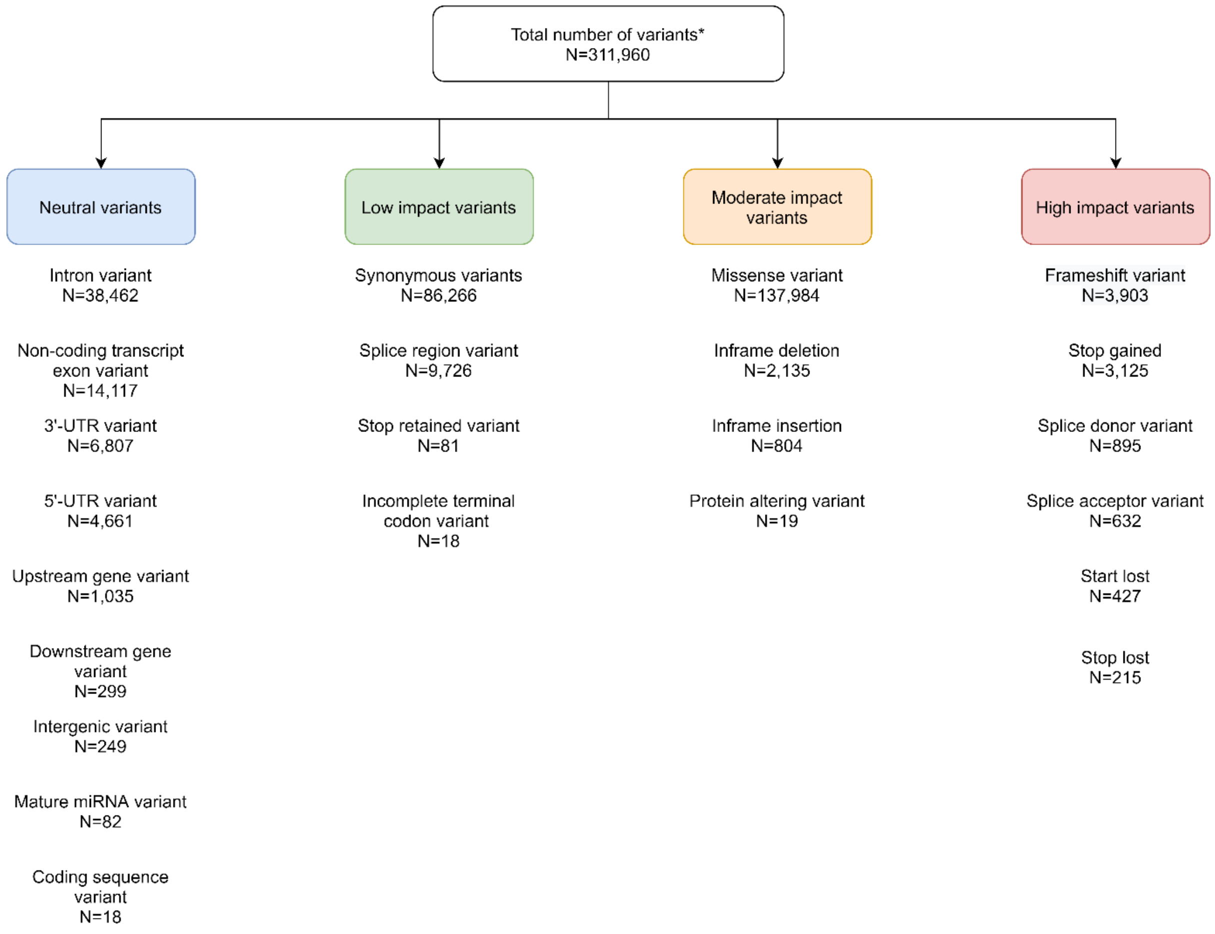

Supplementary Fig. 2: The types and predicted functional impacts of all variants identified through exome sequencing

All variants compared with the hg19 human genome were annotated using the pipeline in Fig. S1b and divided into four classes based on predicted functional impact. For variants with multiple annotations, the most damaging annotation (listed first in variant effect predictor (VEP)) was used. Total variant numbers are shown for the 683 exomes which passed quality control.

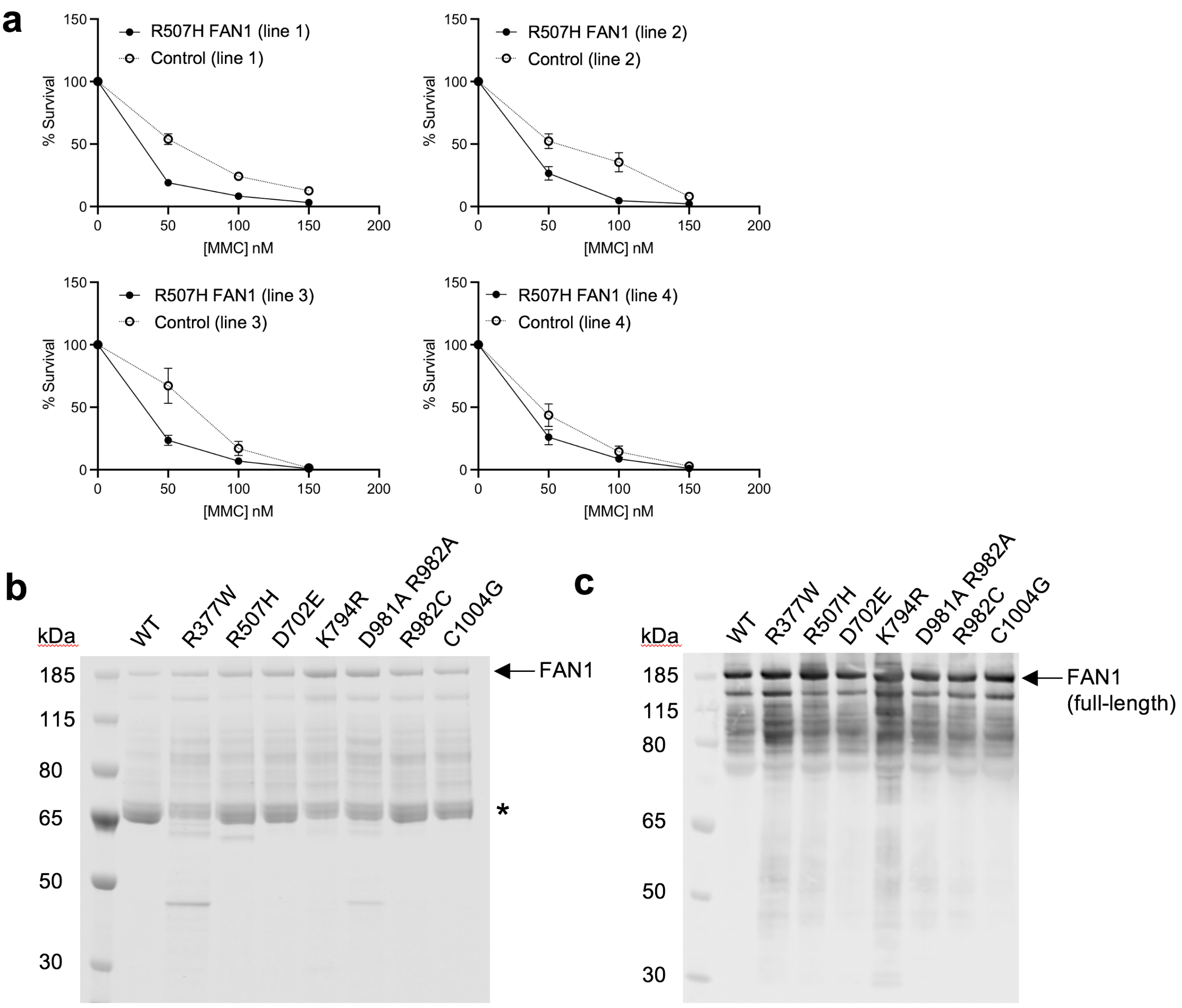

Supplementary Fig. 3: HD lymphoblastoid cells carrying R507H are sensitive to mitomycin C. Partial purification of wild-type and FAN1 variants.

a, 4 lymphoblastoid cell lines derived from individuals with HD carrying a heterozygous R507H FAN1 variant were grown alongside control HD lines with homozygous wild-type FAN1 matched for age and pure CAG length and treated with a single dose of 0-150 nM mitomycin C. Survival (% of untreated live cell count) at 7 days shown for each line (mean, s.e.m., N=3 independent experiments).

b, NusA-His-tagged full-length FAN1 proteins (wild-type (WT) and variants as shown) were expressed in *E. coli* and partially purified using cobalt-agarose. Samples were run on 4-12% Bis-Tris SDS-PAGE and stained with Coomassie blue. Expected size of NusA-His-FAN1 is 175.9 kDa as indicated. FAN1 band intensities were used to normalise the amounts of FAN1 used in nuclease assays. Main contaminants (*) identified by mass spectrometry as *E. coli* chaperones ArnA and DnaK. Further purification of FAN1 proteins was attempted but active protein yields were low.

c, Immunoblot of WT and FAN1 variants run on 4-12% Bis-Tris SDS-PAGE as in **(b)**. Primary antibody was sheep polyclonal to FAN1 (CHDI). Full-length FAN1 is seen along with multiple degradation products.

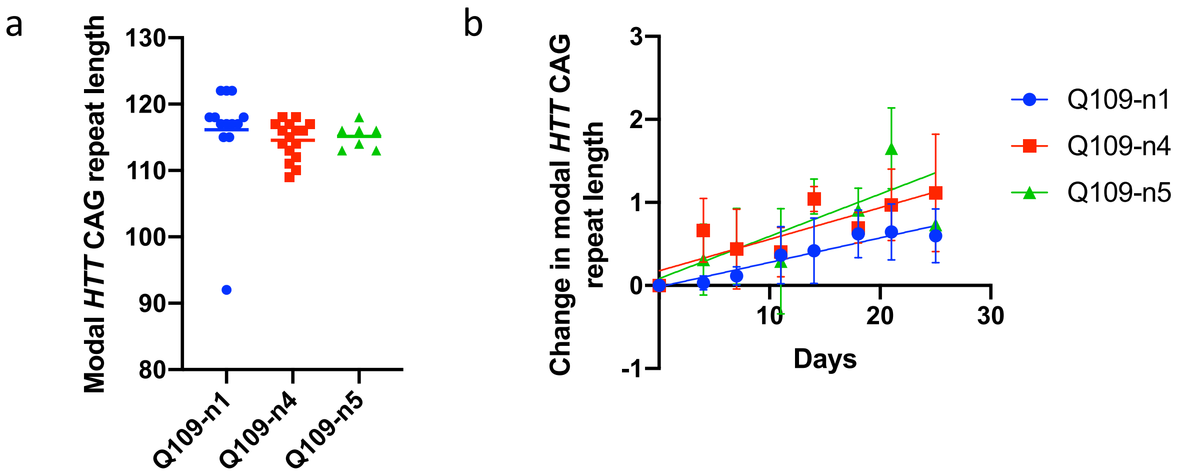

Supplementary Fig. 4: Q109 HD iPSC lines have unstable *HTT* CAG repeats that expand over time in culture

a, Modal CAG repeat lengths for the expanded *HTT* allele of multiple independent, low passage, sub-clones of three independent clonal lines of CS09iHD109 (Q109) HD iPSCs (Q109-n1, n=13; Q109-n4, n=16; Q109-n5, n=7). CAG repeat length mosaicism is demonstrated. Horizontal lines represent means.

b, Modal *HTT* CAG repeat lengths against time in culture as iPSCs for three independent clonal lines of CS09iHD109 (Q109) cells (n1, n4 and n5). Combined data are shown for multiple independent sub-clones of each clonal line (Q109-n1, N=4; Q109-n4, N=3; Q109-n5, N=3). Mean ± s.e.m. shown.

Supplementary Tables

Study Group	N	Sex (% male)[a]	CAG length	Age at Motor Onset (AMO)[b]	Residual AMO[b]
REGISTRY-HD	465	46.6%	42.9 (2.2)	48.4 (16.4)	-0.8 (13.4)
Early (REGISTRY-HD)	214	45.3%	43.4 (2.3)	33.7 (7.8)	-13.9 (4.9)
Late (REGISTRY-HD)	207	48.3%	42.3 (1.8)	63.9 (7.7)	+12.7 (4.2)
PREDICT-HD	218	38.1%	43.3 (2.8)	47.1 (9.8)	-1.0 (5.9)
More severe (PREDICT-HD)	101	31.7%	43.2 (2.7)	46.1 (9.5)	-1.7 (5.5)
Less severe (PREDICT-HD)	115	42.6%	43.4 (3.0)	53.6 (10.1)	+5.0 (5.9)

(a) One individual in the late REGISTRY-HD group was of unknown sex and not included in the sex column
(b) AMO and residual AMO were calculable for all in the REGISTRY-HD group. For the PREDICT-HD group, AMO and AMO residual were calculable for 93 individuals passing initial QC for dichotomous analyses, before phenotype selection (80 were in the more severe group; 11 in the less severe group; 2 in neither extreme group)

Supplementary Table 1: Demographic details for individuals passing quality control and included in the exome sequencing analyses.

Total N=683 (465 REGISTRY-HD group; 218 PREDICT-HD group). Not all individuals were part of the extreme phenotype groups (early/late in REGISTRY-HD, more severe/less severe in PREDICT-HD) after correction for pure CAG repeat lengths following sequencing. Mean (standard deviation) shown for pure CAG length after sequencing, age at motor onset (AMO) and residual AMO.

CAG tract sequence (expanded *HTT*)	Predicted early or worse TMS/SDMT (more severe)	Predicted late or better TMS/SDMT (less severe)
Canonical (CAACAG)	98	111
$(CAACAG)_2$	1	2
$CAC(CAG)_3CAACAG$	0	2
No interruption (pure CAG)	2	0

Supplementary Table 2: CAG repeat tract sequences for PREDICT-HD group, derived from exome sequencing data

Whole-exome sequencing data were extracted and manually assessed to determine the 3' sequence of the CAG repeat tract in pathogenic *HTT* alleles. It was not possible to determine full allelic structures as read lengths were 75 bp. Phase could be determined for atypical alleles as wild-type CAG length was short enough to be effectively captured. The presence or absence of interrupting CAA triplets was used as a covariate in exome analyses. N=216 (those from the dichotomous group).

a

Gene	Dichotomous group (N=637)			Continuous group (N=558)		
	NS	NSD20	LoF	NS	NSD20	LoF
FAN1	**2.32E-04**	*6.61E-04*	2.17E-01	*1.09E-03*	*9.06E-04*	3.40E-01
FAN1 (no R377W/R507H)	*3.76E-02*	*4.37E-02*	2.17E-01	2.85E-01	3.02E-01	3.40E-01

b

Modifier	Top SNP	Location (hg19)	FAN1 protein	p value, all NSD20 variants	p value, all NSD20 variants excluding R377W & R507H
AM1	rs150393409	15:31202961:G:A	R507H	**7.74E-19**	3.89E-01
AM2	rs8034856	15:31240957:T:C	-	7.04E-01	5.67E-01
AM3	rs151322829	15:31197995:C:T	R377W	**2.31E-06**	6.68E-01
AM5	rs118089305	15:31204637:C:T	-	7.96E-02	3.09E-01

Supplementary Table 3: Exome sequencing detects rare variation in *FAN1* associated with altered HD onset that was previously uncaptured by GWAS.

a, *FAN1* retains nominal significance in Optimal Sequence Kernel Association Tests (SKAT-O) of rare coding variants (minor allele frequency < 1%) and HD phenotypes after the exclusion of the R377W and R507H variants previously identified by GeM-HD GWAS (row 2). Variant numbers were regressed on either dichotomous early/more severe or late/less severe phenotypes (N=637; logistic regression) or a continuous phenotype of residual age at motor onset (N=558; linear regression). Phenotypes were corrected for non-canonical *HTT* CAG repeats in expanded alleles by using either a covariate in logistic analyses, or pure CAG lengths from sequencing in continuous analyses. Three different variant groups were tested: all non-synonymous (NS), non-synonymous and predicted damaging to protein function (NSD20; CADD PHRED score ≥ 20), and loss-of-function (LoF). Significant associations in bold ($p < 6.4E-4$, Bonferroni correction for 13 genes and 6 tests; see also Table 1); nominally significant associations in italics ($p < 0.05$)
b, The burden of rare damaging variation in FAN1 is not significantly associated with any of the lead variants from GeM-HD GWAS, after removal of R377W and R507H. Binomial generalised linear models were constructed running the presence of a non-synonymous damaging (NSD20) variant (0 or 1) detected through exome sequencing on the number of copies of each *FAN1* modifier SNP detected through GWAS (0, 1 or 2). These were compared to models running the intercept only, and an ANOVA test was used to compare the two models, for which the *p* values are shown above. AM1, AM2, AM3 and AM5 refer to the independent *FAN1* modifiers previously reported. Note for AM5, the second tagging SNP was used as opposed to rs79213781 as imputation for this SNP was not available in our GWA data. N=441: all those in our dataset with exome sequencing and GWAS data.

Chr	Gene	Dichotomous group (N=637)			Continuous group (N=558)		
		NS	NSD20	LoF	NS	NSD20	LoF
2A	*ANKAR*	8.47E-01	8.28E-01	NA	8.44E-01	6.91E-01	7.86E-01
2A	*ASNSD1*	9.01E-01	8.20E-01	NA	5.61E-01	6.97E-01	NA
2A	*ORMDL1*	3.77E-01	4.34E-01	NA	4.81E-01	5.29E-01	NA
2A	*OSGEPL1*	8.16E-01	8.28E-01	7.80E-01	7.79E-01	7.81E-01	8.47E-01
2A	*PMS1*	**2.72E-02**	**2.65E-03**	3.13E-01	2.62E-01	1.08E-01	NA
3A	*EPM2AIP1*	4.87E-01	4.51E-01	NA	1.00E+00	1.00E+00	NA
3A	*GOLGA4*	8.36E-01	6.72E-01	**3.40E-03**	6.59E-01	2.18E-01	6.30E-02
3A	*LRRFIP2*	3.57E-01	4.91E-01	NA	3.62E-01	8.85E-01	NA
3A	*MLH1*	1.72E-01	5.58E-02	NA	4.99E-01	7.45E-01	NA
3A	*TRANK1*	4.10E-01	5.42E-01	8.98E-01	1.97E-01	3.63E-01	6.07E-01
3A	*C3orf35*	3.21E-01	2.17E-01	2.16E-01	2.01E-01	1.63E-01	1.35E-01
3A	*ITGA9*	1.00E+00	8.51E-01	NA	8.76E-01	8.99E-01	NA
5A	*ANKRD34B*	1.80E-01	8.61E-01	NA	1.64E-01	6.76E-01	NA
5A	*DHFR*	1.05E-01	9.83E-02	NA	1.54E-01	1.42E-01	NA
5A	*FAM151B*	1.51E-01	1.45E-01	NA	5.79E-01	5.77E-01	NA
5A	*MSH3*	5.96E-02	2.78E-01	**9.51E-03**	3.00E-01	6.35E-01	1.09E-01
5A	*MTRNR2L2*	2.22E-01	NA	NA	8.12E-01	NA	NA
5B	*GPR151*	5.48E-01	4.20E-01	5.12E-01	2.65E-01	2.54E-01	2.12E-01
5B	*PPP2R2B*	1.61E-01	2.52E-01	NA	4.82E-01	7.36E-01	8.41E-01
5B	*TCERG1*	**1.25E-02**	4.54E-01	NA	**2.74E-03**	2.76E-01	NA
7A	*AIMP2*	5.16E-02	1.75E-01	7.17E-01	1.66E-01	2.08E-01	1.58E-01
7A	*ANKRD61*	5.25E-01	1.00E+00	2.47E-01	5.05E-01	7.08E-01	6.44E-01
7A	*CCZ1*	1.55E-01	1.84E-01	NA	2.80E-01	2.88E-01	NA
7A	*CYTH3*	8.43E-02	7.99E-02	NA	1.01E-01	1.19E-01	NA
7A	*EIF2AK1*	6.63E-01	7.28E-01	NA	7.05E-01	7.31E-01	NA
7A	*OCM*	8.61E-01	8.65E-01	1.00E+00	1.00E+00	1.00E+00	8.54E-01
7A	*PMS2*	1.00E+00	9.00E-01	5.55E-01	5.59E-01	3.51E-01	1.33E-01
7A	*RSPH10B*	NA	NA	NA	NA	NA	NA
7A	*RSPH10B2*	NA	NA	NA	8.92E-01	NA	NA
7A	*USP42*	7.18E-01	2.49E-01	NA	1.00E+00	3.89E-01	NA
8A	*RRM2B*	1.69E-01	NA	NA	**2.26E-02**	NA	NA
8A	*UBR5*	7.47E-01	7.23E-01	NA	4.11E-01	3.88E-01	NA
11A	*CCDC82*	5.27E-01	NA	NA	6.09E-01	9.01E-01	NA
11A	*JRKL*	2.48E-01	2.42E-01	NA	7.74E-02	7.07E-02	NA
11A	*MAML2*	1.00E+00	8.94E-01	2.08E-01	5.16E-01	4.62E-01	1.31E-01
11B	*SYT9*	1.05E-01	1.13E-01	NA	4.20E-01	4.12E-01	NA
12A	*CORO1C*	8.75E-01	8.69E-01	8.33E-01	6.78E-01	6.66E-01	8.44E-01
12A	*FICD*	3.41E-01	3.42E-01	NA	5.86E-01	7.55E-01	NA
12A	*ISCU*	NA	NA	NA	NA	NA	NA
12A	*SART3*	9.02E-01	7.23E-01	NA	8.15E-01	4.32E-01	NA
12A	*SELPLG*	5.31E-01	8.55E-01	NA	7.91E-01	6.61E-01	NA
12A	*SSH1*	6.77E-02	1.45E-01	5.09E-01	2.77E-01	4.13E-01	9.81E-01
12A	*TMEM119*	5.56E-01	NA	NA	5.28E-01	NA	NA
15A	*FAN1*	**2.32E-04**	**6.61E-04**	2.17E-01	**1.09E-03**	**9.06E-04**	3.40E-01
15A	*MTMR10*	8.11E-01	7.95E-01	NA	3.01E-01	2.77E-01	NA
15A	*TRPM1*	**9.11E-03**	**3.24E-03**	2.28E-01	**1.41E-02**	**6.90E-03**	3.09E-01
16A	*GSG1L*	9.05E-01	9.13E-01	NA	4.09E-01	4.35E-01	NA
18A	*ALPK2*	6.47E-01	1.86E-01	4.06E-01	4.74E-01	8.83E-01	8.50E-01
18A	*MIR122*	NA	NA	NA	NA	NA	NA
19A	*C19orf68*	5.15E-02	5.41E-02	NA	1.80E-01	1.66E-01	NA
19A	*LIG1*	9.47E-02	7.07E-02	5.09E-01	**3.63E-02**	**2.30E-02**	5.16E-01
19A	*PLA2G4C*	8.02E-01	8.68E-01	NA	3.99E-01	7.39E-01	NA

Supplementary Table 4: Candidate gene analysis of rare coding variant associations with HD phenotype for all genes found at candidate modifier loci in GeM-HD GWAS.

Optimal Sequence Kernel Association Tests (SKAT-O) of rare coding variants (minor allele frequency < 1%) and HD phenotypes. Gene-wide variant numbers were regressed on either dichotomous early/more severe or late/less severe phenotypes (N=637; logistic regression) or a continuous phenotype of residual age at motor onset (N=558; linear regression). Phenotypes were corrected for non-canonical *HTT* CAG repeats in expanded alleles by using either a covariate in logistic analyses, or pure CAG lengths from sequencing in continuous analyses. Three different variant groups were tested: all non-synonymous (NS), non-synonymous and predicted damaging to protein function (NSD; CADD PHRED score ≥ 20), and loss-of-function (LoF). Chromosomal loci from GeM-HD GWAS are indicated. Nominally significant associations in bold (p < 0.05). Note that several genes from GeM-HD GWAS loci were not annotated in exome sequencing data and hence not included: *OSGEPL1-AS1* (2A); *JRKL-AS1* + *MIR1260B* (11A); *LOC102723562* (12A); *HERC2P10* + *LOC100288637* + *MIR211* (15A) + *MIR3591* (18A). See also Table 2.

	Early/more severe			Late/less severe		
Gene	NS	NSD20	LoF	NS	NSD20	LoF
PMS1	4	2	0	15	14	1
MLH1	11	10	0	11	5	0
MSH3	8	8	0	19	14	7
DHFR	0	0	0	3	3	0
TCERG1	45	6	0	68	3	0
PMS2	14	2	1	14	1	0
RRM2B	1	0	0	5	0	0
UBR5	7	7	0	5	5	0
CCDC82	4	0	0	4	0	0
SYT9	8	8	0	3	3	0
FAN1	47	34	1	18	11	0
GSG1L	2	2	0	2	2	0
LIG1	11	10	1	16	13	0

Supplementary Table 5: Burden of rare non-synonymous variants of different types in candidate HD modifier genes in the early/more severe and late/less severe phenotype groups

The gene-wide total counts of rare (minor allele frequency <1%) non-synonymous (NS), non-synonymous and predicted damaging to protein function (NSD20; CADD PHRED score ≥ 20) or predicted loss-of-function (LoF) variants in candidate modifier genes from GeM-GWAS are shown for the early/more severe (N=315) and late/less severe (N=322) phenotype groups of the dichotomous association analysis (total N=637). Early/more severe corresponds to early or predicted early onset or high TMS or SDMT. Late/less severe corresponds to late or predicted late onset or low TMS or SDMT. CADD score predicts how damaging individual variants are to protein function. CADD score ≥ 20 implies a variant is in the top 1% predicted most damaging substitutions in the human genome (dbSNFP v4.0[2,3]). See also Table 1.

a

Coordinates	Variant	MAF	CADD score	Early/more severe	Late/less severe
2:190660537:G:A	E59K	4.67E-03	20.4	2	3
2:190660586:C:T	T75I	1.05E-03	24.0	0	4
2:190670391:C:G	T110R	6.17E-05	24.0	0	1
2:190670396:A:G	T112A	8.81E-06	25.6	0	1
2:190717470:CA:C	S264*	NA	NA	0	1
2:190719296:A:G	K433R	3.54E-05	7.5	1	0
2:190719499:G:A	G501R	5.84E-04	25.8	0	1
2:190719569:T:C	L524S	0.00E+00	13.1	1	0
2:190719607:G:A	E537K	2.80E-03	22.8	0	2
2:190719704:G:A	R569Q	1.32E-04	22.1	0	1
2:190732559:T:C	Y793H	5.64E-04	14.5	0	1

b

Coordinates	Variant	MAF	CADD score	Early/more severe	Late/less severe
5:79950562:C:T	P6S	2.65E-04	18.5	0	1
5:79950677:C:G	A44G	1.96E-05	13.2	0	1
5:79952237:A:T	E82V	NA	18.0	0	1
5:79952345:A:T	N118I	4.40E-05	16.0	0	1
5:79966033:G:T	E233*	0.00E+00	48.0	0	1
5:79974830:A:G	S420G	6.16E-05	9.6	0	1
5:80021292:G:A	R454Q	5.29E-05	26.7	1	0
5:80021311:CATTT:C	IY461-462*	NA	NA	0	1
5:80040326:C:T	T552I	6.21E-05	25.4	0	1
5:80057364:G:A	NA (†)	2.64E-05	24.5	0	1
5:80063896:C:T	P681S	1.59E-03	23.6	0	1
5:80063899:G:C	V682L	3.70E-04	24.6	1	0
5:80071512:G:C	NA (†)	NA	28.3	0	1
5:80074538:G:A	NA (†)	NA	34.0	0	1
5:80074556:G:A	R779H	2.29E-04	28.7	0	1
5:80083383:G:A	NA (†)	3.52E-05	24.2	0	1
5:80088565:G:C	E853Q	6.18E-05	26.9	1	1
5:80088589:A:C	N861H	8.81E-06	24.4	0	1
5:80109433:G:T	G896*	4.40E-05	45.0	0	1
5:80109479:T:G	L911W	3.42E-03	26.4	4	2
5:80150135:T:G	D1000E	0.00E+00	22.7	1	0

(†) Predicted loss-of-function splice acceptor variants

(*) Premature stop codon loss-of-function variants

Supplementary Table 6: Rare non-synonymous coding variants in PMS1 and MSH3 identified in extreme phenotype groups of individuals with HD

Allele counts for all rare (MAF < 1%) non-synonymous coding variants in (a) PMS1 and (b) MSH3 identified through exome sequencing in the dichotomous extreme phenotype cohort (N=637). Genomic coordinates are given for hg19. Early/more severe corresponds to early or predicted early onset or high TMS or SDMT. Late/less severe corresponds to late or predicted late onset or low TMS or SDMT. CADD score predicts how damaging individual variants are to protein function. CADD score > 20 implies a variant is in the top 1% predicted most damaging substitutions in the human genome (dbSNFP v4.0[2,3]). Minor allele frequencies (MAF) taken from the European arm of gnomAD[4] v2.1.1.

Dichotomous (N=637)		Continuous (N=558)	
Gene	p	Gene	p
DMGDH	2.92E-04	*CUBN*	1.44E-04
OR4C15	4.20E-04	*ERAP2*	3.96E-04
FAN1	6.61E-04	*ZNF462*	6.18E-04
IGF1R	1.07E-03	*DENND4B*	6.60E-04
MUC6	1.55E-03	*KIAA0319*	8.79E-04
ELP2	1.87E-03	*FAN1*	9.06E-04
LAD1	2.24E-03	*FBP2*	1.06E-03
PEG3	2.34E-03	*SIPA1L2*	1.15E-03
PMS1	2.65E-03	*C9*	1.17E-03
NDOR1	2.67E-03	*NAALAD2*	1.39E-03
C2CD3	3.10E-03	*SAMD3*	1.83E-03
FBP2	3.24E-03	*MUT*	2.10E-03
TRPM1	3.24E-03	*P2RY13*	2.32E-03
RAD54L	3.35E-03	*ANXA11*	2.78E-03
ST7L	3.65E-03	*PPP2R1B*	3.22E-03

Supplementary Table 7: Exome-wide association analysis of the burden of rare, predicted damaging coding variation in genes and HD clinical phenotypes highlights *FAN1*

Exome-wide Optimal Sequence Kernel Association Tests (SKAT-O) were performed using rare (minor allele frequency < 1%), predicted damaging, non-synonymous variants (loss-of-function and/or CADD PHRED score ≥20; NSD20) collapsed on genes, and testing for association with clinical phenotypes. In the dichotomous analysis, association of phenotype (early/more severe or late/less severe) was tested using logistic regression with a covariate for *HTT* CAG repeat structure to account for non-canonical CAG repeats. In the continuous analysis, linear regression was used to test association of variants with residual age at motor onset, corrected for pure CAG lengths. Covariates used: principal components (PCA) 1-5; baseline variant rate (BVR); mean variant depth; study ID (Registry or Predict). Significance threshold = 1.3E-5 (Bonferroni correction for 3912 genes with at least 10 variants). No single gene reached this threshold.

Dichotomous (N=637)			Continuous (N=558)		
GO Term	Description	p	GO Term	Description	p
GO:0042578	phosphoric ester hydrolase activity	**9.66E-05**	GO:0006605	protein targeting	5.96E-03
GO:0006605	protein targeting	2.31E-04	GO:0001654	eye development	6.60E-03
GO:0051090	regulation of DNA binding transcription factor activity	3.16E-04	GO:0046982	protein heterodimerization activity	1.02E-02
GO:0009101	glycoprotein biosynthetic process	3.23E-04	GO:0043010	camera-type eye development	1.26E-02
GO:0050839	cell adhesion molecule binding	4.03E-04	GO:0051259	protein oligomerization	1.48E-02
GO:0009100	glycoprotein metabolic process	4.39E-04	GO:0010008	endosome membrane	1.49E-02
GO:0006511	ubiquitin-dependent protein catabolic process	6.23E-04	GO:0042578	modified amino acid binding	1.54E-02
GO:0019941	modification-dependent protein catabolic process	6.40E-04	GO:0006898	phosphoric ester hydrolase activity	1.61E-02
GO:0004842	ubiquitin-protein transferase activity	6.46E-04	GO:0072341	receptor-mediated endocytosis	1.95E-02
GO:0019787	ubiquitin-like protein transferase activity	7.05E-04	GO:0044440	endosomal part	2.87E-02
GO:0031406	carboxylic acid binding	1.03E-03	GO:0002250	adaptive immune response	3.41E-02
GO:0006935	chemotaxis	1.15E-03	GO:0098609	cell-cell adhesion	3.99E-02
GO:0050711	negative regulation of interleukin-1 secretion	1.16E-03	GO:0034138	negative regulation of interleukin-1 secretion	4.12E-02
GO:0006281	DNA repair	1.23E-03	GO:0031901	toll-like receptor 3 signaling pathway	5.08E-02
GO:0043177	organic acid binding	1.29E-03	GO:0050711	early endosome membrane	5.35E-02
GO:0061564	axon development	1.36E-03	GO:0097193	negative regulation of protein secretion	6.24E-02
GO:0042330	taxis	1.40E-03	GO:0050709	negative regulation of protein transport	6.51E-02
GO:0045088	regulation of innate immune response	1.67E-03	GO:0051224	intrinsic apoptotic signaling pathway	6.65E-02
GO:0051186	cofactor metabolic process	1.77E-03	GO:0001673	male germ cell nucleus	7.21E-02
GO:0035239	tube morphogenesis	2.06E-03	GO:0051962	positive regulation of nervous system development	8.02E-02

Supplementary Table 8: Association of candidate genetic pathways with HD phenotype in both dichotomous and continuous groups

Significant gene pathways from GeM-HD GWAS[5] (*q*<0.001) were selected as candidate pathways (N=233), and gene set membership was taken from the gene ontology (GO) database[6,7]. p values from corrected whole-exome SKAT-O analyses (see also Table S7) were combined across GO pathways and tested for association with HD phenotype using Fisher’s combined probability test. Missing genes or genes with missing p values were excluded. Significance threshold = 1.1E-04 (Bonferroni correction for 233 pathways and 2 groups). Significant p-value in bold.

Name	Use/Target	Sequence
FAN1_Frag2F	Sanger (R377W; L395P)	AGGCAAAATCTCATAGTTCTGCA
FAN1_Frag2R	Sanger (R377W; L395P)	CATCATGCCCCAATCAGAGC
FAN1_Seq2F	Sanger (R377W; L395P)	CAATGATATCCCTCACAGC
FAN1_Seq2R	Sanger (R377W; L395P)	TGAAAACAAACACGTGCG
FAN1_Frag3F	Sanger (D498N; R507H; R507C)	ACTCCTTTCTGCTCCTGAACT
FAN1_Frag3R	Sanger (D498N; R507H; R507C)	CCAGCCTTCTCAATCTAACTACA
FAN1_Seq3F	Sanger (D498N; R507H; R507C)	TCCTTTCTGCTCCTGAAC
FAN1_Seq3R	Sanger (D498N; R507H; R507C)	CCAGCCTTCTCAATCTAAC
FAN1_Frag4F	Sanger (P654L; R658W)	TGGTAGCTGGCTGTGAGAAT
FAN1_Frag4R	Sanger (P654L; R658W)	TCACATGTTTAACGCCATCACA
FAN1_Seq4F	Sanger (P654L; R658W)	TAGCTGGCTGTGAGAATG
FAN1_Seq4R	Sanger (P654L; R658W)	TGTTTAACGCCATCACATC
FAN1_Frag6F	Sanger (K794R)	ACTTTGTGGTAAGGGAGGTCA
FAN1_Frag6R	Sanger (K794R)	CTGGGTGCCACAAGAGAAAG
FAN1_Seq6F	Sanger (K794R)	CTTTTGCTGACCTGAGGC
FAN1_Seq6R	Sanger (K794R)	CCACAAGAGAAAGCCTGC
FAN1_Frag7F	Sanger (V963Wins964L; R969L)	CCATTCTCTGTCACGAGGGA
FAN1_Frag7R	Sanger (V963Wins964L; R969L)	CGGCCCAAAAGCTCTCAAG
FAN1_Seq7F	Sanger (V963Wins964L; R969L)	CGAGGGAAGTGGCTAAC
FAN1_Seq7R	Sanger (V963Wins964L; R969L)	CCTACTTGTGGCCTCTG
FAN1_Frag8F	Sanger (D702E; Q717R)	CAGTGAGAGAGCAGAAGAGC
FAN1_Frag8R	Sanger (D702E; Q717R)	TGGGTGACAGAGCGAGACT
FAN1_Seq8F	Sanger (D702E; Q717R)	AGTGAGAGAGCAGAAGAG
FAN1_Seq8R	Sanger (D702E; Q717R)	ACCAAATATCCCCAATTCC
FAN1_Frag10F	Sanger (R982C; C1004G)	CAGTGAGAGAGCAGAAGAGC
FAN1_Frag10R	Sanger (R982C; C1004G)	ACTGTGTGGAATCAATGAGTGT
FAN1_Seq10F	Sanger (R982C; C1004G)	AGTGAGAGAGCAGAAGAG
FAN1_Seq10R	Sanger (R982C; C1004G)	TGTGTGGAATCAATGAGTG
FAN1_Frag12F	Sanger (M50R; V77I)	TCAGAGTTCGCTTTTCCCCT
FAN1_Frag12R	Sanger (M50R; V77I)	CACACTACGATTTCTCAGCTCA
FAN1_Seq12F	Sanger (M50R; V77I)	ACTCATGATGTCAGAAGGG
FAN1_Seq12R	Sanger (M50R; V77I)	TTGCTGAATCACTTTGGC
FAN1_Frag13F	Sanger (T187fs)	GGGAAGTAAAGCAGAAGATCAGT
FAN1_Frag13R	Sanger (T187fs)	TTCTCACATTCCCGGGTAGC
FAN1_Seq13F	Sanger (T187fs)	GCTGAGAAATCGTAGTGTG
FAN1_Seq13R	Sanger (T187fs)	GTTCAGGAATGCACTCTTC
FAM-huHTT-exon 1 F	Capillary electrophoresis *HTT* CAG	FAM-ATGAAGGCCTTCGAGTCCCTCAAGTCCTTC
huHTT-exon 1 R	Capillary electrophoresis *HTT* CAG	GGCGGCTGAGGAAGCTGAGGA
TOM112	This paper, based on [8]	TTTTTGTCTGAATCTGGTGCTGGGATCCAACATGTTCTAAC
TOM117	This paper, based on [8]	GTTAGAACATGTTGGATCCCAGCACCAGATTCAGACCTGATGACCGATCGTACGTTCGCTGCTGGCTACTGC
TOM122	This paper, based on [8]	GCAGTAGCCAGCAGCGAACGTACGATCGGTCATCAG
FAN1 KO gRNA1	gRNA *FAN1* exon 2: FAN1 knock-out	CTGATTGATAAGCTTCTACGAGG
FAN1 KO gRNA2	gRNA *FAN1* exon 2: FAN1 knock-out	GCACCATTTTACTGCAAACGGGG

FAN1 D960A gRNA	gRNA *FAN1* exon 13: D960A variant	AGGGGGCCTCCCCGACCTGGTGG
FAN1 D960A repair	D960A repair template	GTGCTCAGTGGTGTGTGCAGGCACCTGGCTGCTGACTTTCGACA CTGTCGAGGaGGCCTCCCCGcCCTGGTcGTGTGGAACTCCCAGA GCCGTCACTTTAAGGTCAGTTGAGGCAGAATGGA
FAN1-KO F	*FAN1* KO check	CCTGTGTTTTATTGCTCAGAACA
FAN1-KO R	*FAN1* KO check	CATTTCATCAAGGTGCCGGT
D960A FauI/StuI F	FAN1 D960A check	TCACGAGGGAAGTGGCTAAC
D960A FauI R	FAN1 D960A check	GCCAACAGCCACTCAAGAAATG
D960A StuI R	FAN1 D960A check	CACAGAATACAGCAGGAGTGATG

Supplementary Table 9: Oligonucleotides used in this study

Primers marked by ‘Sanger’ were used for sanger sequencing of *FAN1* variants in select individuals. *FAN1* Q717R was confirmed both by exome QC and Sanger sequencing to not exist (this was originally called but later refuted in the QC pipeline).

REAGENT	SOURCE	IDENTIFIER
**Antibodies**		
Anti-FAN1 (sheep polyclonal, second bleed)	CHDI	CHDI
Anti-β-Tubulin Antibody, clone AA2	Upstate	Cat#05-661 Lot#22237
Donkey anti-Mouse IgG (H+L) Highly Cross-Adsorbed Secondary Antibody, Alexa Fluor Plus 680	Invitrogen	Cat#A32788
IRDye® 800CW Donkey anti-Goat IgG Secondary Antibody	LI-COR	Cat#926-32214 Lot#C80717-07
Anti-OCT4 antibody	AbCam	Cat#ab19857
Anti-MAP2 antibody	AbCam	Cat#ab32454
Anti-CTIP2 antibody	AbCam	Cat#ab18465
Alexa Fluor goat anti-mouse IgG 488	Invitrogen	Cat#A11001
Alexa Fluor goat anti-rabbit IgG 568	Invitrogen	Cat#A11011
**Biological Samples**		
Genomic DNA from lymphoblastoid cell lines derived from HD patients in Registry study	CHDI	EHDN projects 0791 and 0803
**Chemicals**		
Essential 8 Flex Medium Kit	Life Technologies	Cat#A2858501
Advanced DMEM/F-12	Life Technologies	Cat#12634028
Knockout DMEM/F-12	Life Technologies	Cat#12660012
Neurobasal™ Medium	Life Technologies	Cat#21103049
Corning® Matrigel® Growth Factor Reduced (GFR) Basement Membrane Matrix, LDEV-free	BD Biosciences	Cat#354230
Poly-D-Lysine hyrobromide	Sigma-Aldrich	Cat#P6407
GlutaMAX	Thermo Fisher	Cat#35050-038
Penicillin/ Streptomycin (5000U/5000 µg)	Gibco	Cat#15070063
MACS NeuroBrew-21	Miltenyi	Cat#130-093-566
MACS NeuroBrew-21 (w/o Vitamin A)	Miltenyi	Cat#130-097-263
StemMACS™ IWR-1-endo	Miltenyi	Cat#130-110-491
Human BDNF, research grade	Miltenyi	Cat#130-096-286

StemMACS™ SB431542	Miltenyi	Cat#130-105-336
LDN -193189	StemGent	Cat#04-0019
Recombinant Human/Murine/Rat Activin A (Insect derived)	PeproTech	Cat#120-14
CHIR 99021	Bio-Techne	Cat#4423
PD0332991	Bio-Techne	Cat#4786
DAPT	Bio-Techne	Cat#2634
LM22A4	Bio-Techne	Cat#4607
Forskolin	Bio-Techne	Cat#1099
Ascorbic Acid	Sigma-Aldrich	Cat#A4544
Calcium Chloride	Sigma-Aldrich	Cat#499609-1G
γ-Aminobutyric acid (GABA)	Bio-Techne	Cat#0344
Y-27632 dihydrochloride (ROCK inhibitor)	Tocris	Cat#1254
ReLeSR	Stem Cell Technologies	Cat#05873
StemPro Accutase Cell Dissociation Reagent	Life Technologies	Cat#A1110501
Gentle Cell Dissociation Reagent	Stem Cell Technologies	Cat#07174
Hoechst 33342	Life Technologies	Cat#62249
TaKaRa LA Taq® DNA Polymerase with GC Buffer	Takara	Cat#RR02AG
Taq polyermase	Sigma-Aldrich	Cat#D4545
Custom 10X mix for PCR	Thermo Scientific [9]	Cat#SM-0005
AMPure XP SPRI beads	Beckman Coulter	Cat#A63881
OneTaq Hot Start DNA Polymerase	NEB	Cat#M0481L
FauI	NEB	Cat#R0651S
StuI	NEB	Cat#R0187S
CutSmart Buffer	NEB	Cat#B7204S
Hi-Di™ Formamide	Applied Biosystems	Cat# 4440753
Duplex buffer	IDT	Cat#11-05-01-03
Alt-R S.p. Cas9 nuclease, v.3	IDT	Cat# 1081058
cOmplete™, EDTA-free Protease Inhibitor Cocktail Tablets	Merck	Cat# 11873580001
**Commercial Assays & Kits**		
QIAamp DNA Mini Kit	QIAGEN	Cat#51306
QuickExtract DNA Extraction Solution	Cambio	Cat#QE09050
P3 Primary Cell 4D-Nucleofector™ X Kit L	Lonza	Cat#V4XP-3024
QIAquick PCR Purification Kit	QIAGEN	Cat#28104
MyTaq™	Bioline	Cat#BIO21127
Quant-iT™ PicoGreen™	ThermoFisher	Cat#P7589
TruSeq Rapid Exome Kit	Illumina	Cat#20020617
High sensitivity DNA chip for Bioanalyser	Agilent	Cat#5067-4626
HiSeq 3000/4000 PE Cluster Kit	Illumina	Cat#PE-410-1001
HiSeq 3000/4000 SBS kit (150 cycles)	Illumina	Cat#FC-410-1002
MiSeq Reagent Kit v3 (600-cycle)	Illumina	Cat#MS-102-3003
GeneScan™ 600 LIZ™ dye Size Standard v2.0	Applied Biosystems	Cat# 4408399
**Databases**		
dbNSFP	[2,3]	4.0
gnomAD	[4]	2.1.1
PREDICT-HD exomes	Genetic Modifiers of Huntington's Disease	**dbGaP Study Accession:** phs000371.v2.p1

REGISTRY-HD	http://www.ehdn.org/; [10]	R3 Cut
**Cell Lines**		
Human: HD iPSC lines (Q109N1, Q109N5)	[11]	
N1-FAN1$^{-/-}$ iPSC lines	This paper	N/A
N5-FAN1$^{-/-}$ iPSC lines	This paper	N/A
N5-FAN1$^{D960A/D960A}$ iPSC lines	This paper	N/A
N5-FAN1$^{D960A/WT}$ iPSC lines	This paper	N/A
Lymphoblastoid cell lines	CHDI	EHDN projects 0791 and 0803
**Commercial Oligonucleotides**		
Alt-R® CRISPR-Cas9 tracrRNA, ATTO™ 550	IDT	Cat#1075928
**Software and Algorithms**		
Burrows-Wheeler Aligner (BWA)	[12]	0.7.5a
Fragman software	CRAN.R-project.org/package=Fragman	1.0.9
GeneMapper Software	Applied Biosystems	4.1
Genome analysis toolkit (GATK)	[13–15]	3.6-0-g89b7209
GraphPad Prism	GraphPad Software, https://www.graphpad.com/scientific-software/prism/	8.2.1
Hail	https://github.com/hail-is/	0.1-5a67787
Peddy	[1]	0.3.5
Picard (picard-tools)	https://github.com/broadinstitute/picard/	1.97
Primer3	[16,17]	Accessed June 2020
R	https://www.r-project.org/	3.6.0
SAMtools	[18,19]	1.9
Scale-HD	https://github.com/helloabunai/ScaleHD/	0.322
Tablet	[20]	1.19.05.28
UCSC In-Silico PCR	https://genome.ucsc.edu/cgi-bin/hgPcr	Accessed June 2020
Variant-effect predictor tool (VEP)	[21]	95
VerifyBamID	[22]	1.1.3

Supplementary Table 10: Reagents used in this study

Supplementary References

1. Pedersen, B. S. & Quinlan, A. R. Who's Who? Detecting and Resolving Sample Anomalies in Human DNA Sequencing Studies with Peddy. *Am. J. Hum. Genet.* **100**, 406–413 (2017).
2. Liu, X., Wu, C., Li, C. & Boerwinkle, E. dbNSFP v3.0: A One-Stop Database of Functional Predictions and Annotations for Human Nonsynonymous and Splice-Site SNVs. *Hum. Mutat.*

37, 235–41 (2016).
3. Liu, X., Jian, X. & Boerwinkle, E. dbNSFP: a lightweight database of human nonsynonymous SNPs and their functional predictions. *Hum. Mutat.* **32**, 894–9 (2011).
4. Karczewski, K. J. *et al.* The mutational constraint spectrum quantified from variation in 141,456 humans. *Nature* **581**, 434–443 (2020).
5. GeM-HD Consortium. CAG Repeat Not Polyglutamine Length Determines Timing of Huntington's Disease Onset. *Cell* **178**, 887–900 (2019).
6. Ashburner, M. *et al.* Gene ontology: tool for the unification of biology. The Gene Ontology Consortium. *Nat. Genet.* **25**, 25–9 (2000).
7. The Gene Ontology Consortium. The Gene Ontology Resource: 20 years and still GOing strong. *Nucleic Acids Res.* **47**, D330–D338 (2019).
8. Rao, T. *et al.* Importance of homo-dimerization of Fanconi-associated nuclease 1 in DNA flap cleavage. *DNA Repair (Amst).* **64**, 53–58 (2018).
9. Ciosi, M. *et al.* Library preparation and MiSeq sequencing for the genotyping-by-sequencing of the Huntington disease HTT exon one trinucleotide repeat and the quantification of somatic mosaicism. *Protoc. Exch.* (2018). doi:10.1038/protex.2018.089
10. Orth, M. *et al.* Observing Huntington's Disease: the European Huntington's Disease Network's REGISTRY. *PLoS Curr.* **2**, RRN1184 (2010).
11. HD iPSC Consortium. Induced pluripotent stem cells from patients with Huntington's disease show CAG-repeat-expansion-associated phenotypes. *Cell Stem Cell* **11**, 264–78 (2012).
12. Li, H. & Durbin, R. Fast and accurate short read alignment with Burrows-Wheeler transform. *Bioinformatics* **25**, 1754–60 (2009).
13. McKenna, A. *et al.* The Genome Analysis Toolkit: a MapReduce framework for analyzing next-generation DNA sequencing data. *Genome Res.* **20**, 1297–303 (2010).
14. DePristo, M. A. *et al.* A framework for variation discovery and genotyping using next-generation DNA sequencing data. *Nat. Genet.* **43**, 491–8 (2011).
15. Van der Auwera, G. A. *et al.* From FastQ data to high confidence variant calls: the Genome Analysis Toolkit best practices pipeline. *Curr. Protoc. Bioinforma.* **43**, 11.10.1-33 (2013).
16. Untergasser, A. *et al.* Primer3--new capabilities and interfaces. *Nucleic Acids Res.* **40**, e115 (2012).
17. Koressaar, T. & Remm, M. Enhancements and modifications of primer design program Primer3. *Bioinformatics* **23**, 1289–91 (2007).
18. Li, H. *et al.* The Sequence Alignment/Map format and SAMtools. *Bioinformatics* **25**, 2078–9 (2009).
19. Li, H. A statistical framework for SNP calling, mutation discovery, association mapping and population genetical parameter estimation from sequencing data. *Bioinformatics* **27**, 2987–93 (2011).
20. Milne, I. *et al.* Using Tablet for visual exploration of second-generation sequencing data. *Brief. Bioinform.* **14**, 193–202 (2013).
21. McLaren, W. *et al.* The Ensembl Variant Effect Predictor. *Genome Biol.* **17**, 122 (2016).
22. Jun, G. *et al.* Detecting and estimating contamination of human DNA samples in sequencing and array-based genotype data. *Am. J. Hum. Genet.* **91**, 839–48 (2012).

Source Data for Supplementary Figures

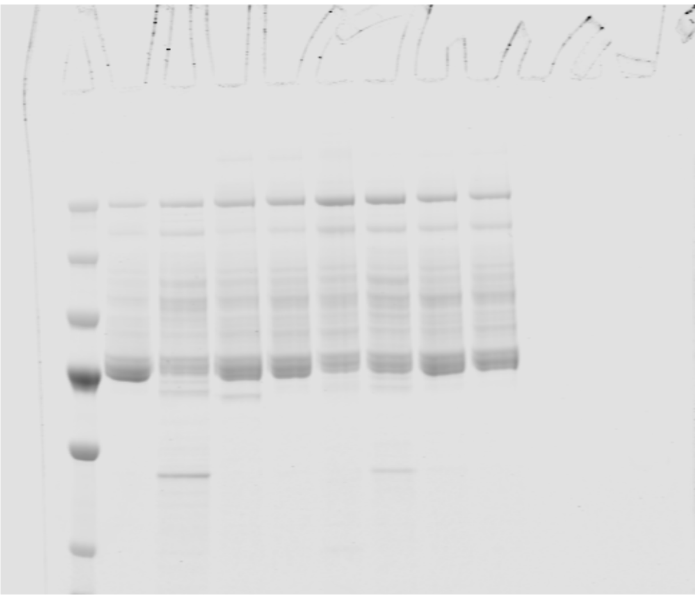

Fig. 3b. 4-12% Bis-Tris SDS-PAGE stained with Coomassie blue

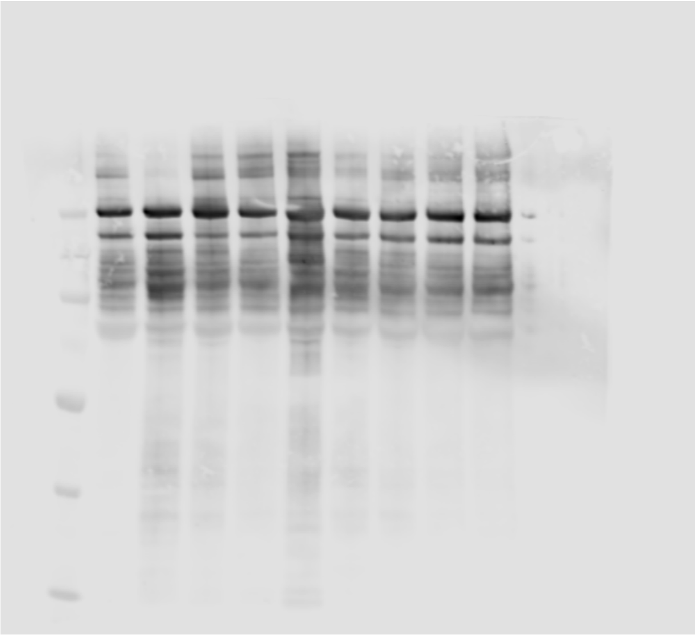

Fig. 3c. 4-12% Bis-Tris SDS-PAGE. Immunoblotted with anti-FAN1 sheep polyclonal antibody

Members of Consortia

REGISTRY Investigators of the European Huntington's disease network

REGISTRY Steering committee: Anne-Catherine Bachoud-Lévi, Anna Rita Bentivoglio, Ida Biunno, Raphael M Bonelli, Jean-Marc Burgunder, Stephen B Dunnett, Joaquim J Ferreira, Olivia J. Handley, Arvid Heiberg, Torsten Illmann, G Bernhard Landwehrmeyer, Jamie Levey, Maria A. Ramos-Arroyo, Jørgen E Nielsen, Susana Pro Koivisto, Markku Päivärinta, Raymund A.C. Roos, Ana Rojo Sebastián, Sarah J Tabrizi, Wim

Vandenberghe, Christine Verellen-Dumoulin, Tereza Uhrova, Jan Wahlström, Jacek Zaremba

Language coordinators: Verena Baake, Katrin Barth, Monica Bascuñana Garde, Sabrina Betz, Reineke Bos, Jenny Callaghan, Adrien Come, Leonor Correia Guedes, Daniel Ecker, Ana Maria Finisterra, Ruth Fullam, Mette Gilling, Lena Gustafsson, Olivia J. Handley, Carina Hvalstedt, Christine Held, Kerstin Koppers, Claudia Lamanna, Matilde Laurà, Asunción Martínez Descals, Saül Martinez-Horta, Tiago Mestre, Sara Minster, Daniela Monza, Lisanne Mütze, Martin Oehmen, Michael Orth, Hélène Padieu, Laurent Paterski, Nadia Peppa, Susana Pro Koivisto, Martina Di Renzo, Amandine Rialland, Niini Røren, Pavla Šašinková, Erika Timewell, Jenny Townhill, Patricia Trigo Cubillo, Wildson Vieira da Silva, Marleen R van Walsem, Carina Whalstedt, Marie-Noelle Witjes-Ané , Grzegorz Witkowski, Abigail Wright, Daniel Zielonka, Eugeniusz Zielonka, Paola Zinzi

AUSTRIA

Graz (Medizinische Universitäts Graz, Psychiatrie): Raphael M Bonelli, Sabine Lilek, Karen Hecht, Brigitte Herranhof, Anna Holl (formerly Hödl), Hans-Peter Kapfhammer, Michael Koppitz, Markus Magnet, Nicole Müller, Daniela Otti, Annamaria Painold, Karin Reisinger, Monika Scheibl, Helmut Schöggl, Jasmin Ullah

Innsbruck (Universitätsklinik Innsbruck, Neurologie): Eva-Maria Braunwarth, Florian Brugger, Lisa Buratti, Eva-Maria Hametner, Caroline Hepperger, Christiane Holas, Anna Hotter, Anna Hussl, Christoph Müller, Werner Poewe, Klaus Seppi, Fabienne Sprenger, Gregor Wenning

BELGIUM

Bierbeek: Andrea Boogaerts, Godelinde Calmeyn, Isabelle Delvaux, Dirk Liessens, Nele Somers

Charleroi (Institut de Pathologie et de Génétique (IPG)): Michel Dupuit, Cécile Minet, Dominique van Paemel, Pascale Ribaï, Christine Verellen-Dumoulin

Leuven: (Universitair Ziekenhuis Gasthuisberg,): Andrea Boogaerts, Wim Vandenberghe, Dimphna van Reijen

CZECH REPUBLIC

Prague (Extrapyramidové centrum, Neurologická klinika, 1. LF UK a VFN): Jiří Klempíř, Veronika Majerová, Jan Roth, Irena Stárková

DENMARK

Copenhagen (Neurogenetics Clinic, Danish Dementia Research Centre, Rigshospitalet, University of Copenhagen): Lena E. Hjermind, Oda Jacobsen, Jørgen E Nielsen, Ida Unmack Larsen, Tua Vinther-Jensen

FINLAND

Turku-Suvituuli (Rehabilitation Centre Suvituuli): Heli Hiivola, Hannele Hyppönen, Kirsti Martikainen, Katri

Tuuha

FRANCE

Angers (Centre de référence des maladies neurogénétique- CHU d'Angers): Philippe Allain, Dominique Bonneau, Marie Bost, Bénédicte Gohier, Marie-Anne Guérid, Audrey Olivier, Adriana Prundean, Clarisse Scherer-Gagou, Christophe Verny

Bordeaux (Hôpital CHU Pellegrin): Blandine Babiloni, Sabrina Debruxelles, Charlotte Duché, Cyril Goizet, Laetitia Jameau, Danielle Lafoucrière, Umberto Spampinato

Lille-Amiens:

Lille (CHRU Roger Salengro) : Rekha Barthélémy, Christelle De Bruycker, Maryline Cabaret, Anne-Sophie Carette, Eric Decorte Luc Defebvre, Marie Delliaux, Arnaud Delval, Alain Destee, Kathy Dujardin, Marie-Hélène Lemaire, Sylvie Manouvrier, Mireille Peter, Lucie Plomhouse, Bernard Sablonnière, Clémence Simonin, Stéphanie Thibault-Tanchou, Isabelle Vuillaume

Amiens (CHU Nord) : Marcellin Bellonet, Hassan Berrissoul, Stéphanie Blin, Françoise Courtin, Cécile Duru, Véronique Fasquel, Olivier Godefroy, Pierre Krystkowiak, Béatrice Mantaux, Martine Roussel, Sandrine Wannepain

Marseille (Hôpital La Timone) : Jean-Philippe Azulay, Marie Delfini, Alexandre Eusebio, Frédérique Fluchere, Laura Mundler

Strasbourg (Hôpital Civil) : Mathieu Anheim, Celine Julié, Ouhaid Lagha Boukbiza, Nadine Longato, Gabrielle Rudolf, Christine Tranchant, Marie-Agathe Zimmermann

GERMANY

Aachen (Universitätsklinikum Aachen, Neurologische Klinik): Christoph Michael Kosinski, Eva Milkereit, Daniela Probst, Kathrin Reetz, Christian Sass, Johannes Schiefer, Christiane Schlangen, Cornelius J. Werner

Berlin (Klinik und Poliklinik für Neurologie - Charité - Universitätsmedizin Berlin): Harald Gelderblom, Josef Priller, Harald Prüß, Eike Jakob Spruth

Bochum (Huntington-Zentrum (NRW) Bochum im St. Josef-Hospital): Gisa Ellrichmann, Lennard Herrmann, Rainer Hoffmann, Barbara Kaminski, Peter Kotz, Christian Prehn, Carsten Saft

Dinslaken (Reha Zentrum in Dinslaken im Gesundheitszentrums Lang): Herwig Lange, Robert Maiwald

Dresden (Universitätsklinikum Carl Gustav Carus an der Technischen Universität Dresden, Klinik und Poliklinik für Neurologie): Matthias Löhle, Antonia Maass, Simone Schmidt, Cecile Bosredon, Alexander Storch, Annett Wolz, Martin Wolz

Freiburg (Universitätsklinik Freiburg, Neurologie): Philipp Capetian, Johann Lambeck, Birgit Zucker

Hamburg (Universitätsklinikum Hamburg-Eppendorf, Klinik und Poliklinik für Neurologie): Kai Boelmans, Christos Ganos, Walburgis Heinicke, Ute Hidding, Jan Lewerenz, Alexander Münchau, Michael Orth, Jenny Schmalfeld, Lars Stubbe, Simone Zittel

Hannover (Neurologische Klinik mit Klinischer Neurophysiologie, Medizinische Hochschule Hannover): Gabriele Diercks, Dirk Dressler, Heike Gorzolla, Christoph Schrader, Pawel Tacik

Itzehoe (Schwerpunktpraxis Huntington, Neurologie und Psychiatrie): Michael Ribbat

Marburg KPP (Klinik für Psychiatrie und Psychotherapie Marburg-Süd): Bernhard Longinus

Marburg Uni (Universität Marburg, Neurologie): Katrin Bürk, Jens Carsten Möller, Ida Rissling

München (Huntington-Ambulanz im Neuro-Kopfzentrum - Klinikum rechts der Isar der Neurologischen Klinik und Poliklinik der Technischen Universität München): Mark Mühlau, Alexander Peinemann, Michael Städtler, Adolf Weindl, Juliane Winkelmann, Cornelia Ziegler

Münster (Universitätsklinikum Münster, Klinik und Poliklinik für Neurologie): Natalie Bechtel, Heike Beckmann, Stefan Bohlen, Eva Hölzner, Herwig Lange, Ralf Reilmann, Stefanie Rohm, Silke Rumpf , Sigrun Schepers, Natalia Weber

Taufkirchen (Isar-Amper-Klinikum - Klinik Taufkirchen (Vils)): Matthias Dose, Gabriele Leythäuser, Ralf Marquard, Tina Raab, Alexandra Wiedemann

Ulm (Universitätsklinikum Ulm, Neurologie): Katrin Barth, Andrea Buck, Julia Connemann, Daniel Ecker, Carolin Geitner, Christine Held, Andrea Kesse, Bernhard Landwehrmeyer, Christina Lang, Jan Lewerenz, Franziska Lezius, Solveig Nepper, Anke Niess, Michael Orth, Ariane Schneider, Daniela Schwenk, Sigurd Süßmuth, Sonja Trautmann, Patrick Weydt

ITALY

Bari Clinica Neurologica - Neurophysiopatology of Pain Unit UNIVERSITA' DI BARI): Claudia Cormio, Vittorio Sciruicchio, Claudia Serpino, Marina de Tommaso

Bologna (DIBINEM - Alma Mater Studiorum - Università di Bologna; IRCCS Istituto delle Scienze Neurologiche di Bologna): Sabina Capellari, Pietro Cortelli, Roberto Galassi, Rizzo Giovanni, Roberto Poda, Cesa Scaglione

Florence (Dipartimento di Scienze Neurologiche e Psichiatriche Universita' degli Studi di Firenze-Azienda Ospedaliera Universitaria Careggi): Elisabetta Bertini, Elena Ghelli, Andrea Ginestroni, Francesca Massaro, Claudia Mechi, Marco Paganini, Silvia Piacentini, Silvia Pradella, Anna Maria Romoli, Sandro Sorbi

Genoa (Dipartimento di Neuroscienze, Riabilitazione, Oftalmologia, Genetica e Scienze Materno-Infantili, Università di Genova): Giovanni Abbruzzese, Monica Bandettini di Poggio, Giovanna Ferrandes,

Paola Mandich, Roberta Marchese

Milan (Fondazione IRCCS Istituto Neurologico Carlo Besta):

Alberto Albanese, Daniela Di Bella, Anna Castaldo, Stefano Di Donato, Cinzia Gellera, Silvia Genitrini, Caterina Mariotti, Daniela Monza, Lorenzo Nanetti, Dominga Paridi, Paola Soliveri, Chiara Tomasello

Naples (Dipartimento di Neuroscienze, Scienze Riproduttive e Odontostomatologiche, Università Federico II): Giuseppe De Michele, Luigi Di Maio, Marco Massarelli, Silvio Peluso, Alessandro Roca, Cinzia Valeria Russo, Elena Salvatore, Pierpaolo Sorrentino

Pozzilli (IS) (Centro di Neurogenetica e Malattie Rare - IRCCS Neuromed): Enrico Amico, Mariagrazia Favellato, Annamaria Griguoli, Irene Mazzante, Martina Petrollini, Ferdinando Squitieri and **Rome (Lega Italiana Ricerca Huntington e malattie correlate - onlus / www.LIRH.it):** Barbara D'Alessio, Chiara Esposito

Rome (Istituto di Farmacologia Traslazionale & Istituto di Scienze e Tecnologie della Cognizione /CNR, Istituto di Neurologia Università Cattolica del Sacro Cuore): Anna Rita Bentivoglio, Marina Frontali, Arianna Guidubaldi, Tamara Ialongo, Gioia Jacopini, Carla Piano, Silvia Romano, Francesco Soleti, Maria Spadaro, Paola Zinzi

NETHERLANDS

Enschede (Medisch Spectrum Twente): Monique S.E. van Hout, Marloes E. Verhoeven, Jeroen P.P. van Vugt, A. Marit de Weert

Groningen (Polikliniek Neurologie): J.J.W. Bolwijn, M. Dekker, B. Kremer, K.L. Leenders, J.C.H. van Oostrom

Leiden (Leiden University Medical Centre (LUMC)): Simon J. A. van den Bogaard, Reineke Bos, Eve M. Dumas, Ellen P. ‘t Hart, Raymund A.C. Roos

Nijmegen (Universitair Medisch Centrum St. Radboud, Neurology): Berry Kremer, C.C.P. Verstappen

NORWAY

Oslo University Hospital (Rikshospitalet, Dept. of Medical Genetics and Dept. of Neurology): Olaf Aaserud, Jan Frich C., Arvid Heiberg, Marleen R. van Walsem, Ragnhild Wehus

Oslo University Hospital (Ulleval, **Dept. of Medical Genetics and Dept.of Neurorehabilitation)**: Kathrine Bjørgo, Madeleine Fannemel, Per F. Gørvell, Eirin Lorentzen, Susana Pro Koivisto, Lars Retterstøl, Bodil Stokke

Trondheim (St. Olavs Hospital): Inga Bjørnevoll, Sigrid Botne Sando

POLAND

Gdansk (St. Adalbert Hospital, Gdansk, Medical University of Gdansk, Neurological and Psychiatric

Nursing Dpt.): Artur Dziadkiewicz, Malgorzata Nowak, Piotr Robowski, Emilia Sitek, Jaroslaw Slawek, Witold Soltan, Michal Szinwelski

Katowice (Medical University of Silesia, Katowice): Magdalena Blaszcyk, Magdalena Boczarska-Jedynak, Ewelina Ciach-Wysocka, Agnieszka Gorzkowska, Barbara Jasinska-Myga, Gabriela Kłodowska–Duda, Gregorz Opala, Daniel Stompel

Krakow (Krakowska Akademia Neurologii): Krzysztof Banaszkiewicz, Dorota Boćwińska, Kamila Bojakowska-Jaremek, Małgorzata Dec, Malgorzata Krawczyk, Monika Rudzińska, Elżbieta Szczygieł, Andrzej Szczudlik, Anna Wasielewska, Magdalena Wójcik

Poznan (Poznan University of Medical Sciences, Poland): Anna Bryl, Anna Ciesielska, Aneta Klimberg, Jerzy Marcinkowski, Husam Samara, Justyna Sempołowicz, Daniel Zielonka

Warsaw-MU (Medical University of Warsaw, Neurology): Anna Gogol (formerly Kalbarczyk), Piotr Janik, Hubert Kwiecinski, Zygmunt Jamrozik

Warsaw-IPiN (Institute of Psychiatry and Neurology Dep. of Genetics, First Dep. of Neurology): Jakub Antczak, Katarzyna Jachinska, Wioletta Krysa, Maryla Rakowicz, Przemyslaw Richter, Rafal Rola, Danuta Ryglewicz, Halina Sienkiewicz-Jarosz, Iwona Stępniak, Anna Sułek, Grzegorz Witkowski, Jacek Zaremba, Elzbieta Zdzienicka, Karolina Zieora-Jakutowicz

PORTUGAL

Coimbra (Hospital Universitário de Coimbra): Cristina Januário, Filipa Júlio

Lisbon (Clinical Pharmacology Unit, Instituto de Medicina Molecular, Faculty of Medicine, University of Lisbon): Joaquim J Ferreira, Miguel Coelho, Leonor Correia Guedes, Tiago Mendes, Tiago Mestre, Anabela Valadas

Porto (Hospital de São João, (Faculdade de Medicina da Universidade do Porto)): Carlos Andrade, Miguel Gago, Carolina Garrett, Maria Rosália Guerra.

SPAIN

Badajoz (Hospital Infanta Cristina): Carmen Durán Herrera, Patrocinio Moreno Garcia

Barcelona-Hospital Mútua de Terrassa: Miquel Aguilar Barbera, Dolors Badenes Guia, Laura Casas Hernanz , Judit López Catena, Pilar Quiléz Ferrer, Ana Rojo Sebastián, Gemma Tome Carruesco

Barcelona-Bellvitge (Hospital Universitari de Bellvitge): Jordi Bas, Núria Busquets, Matilde Calopa

Barcelona-Merced (Hospital Mare de Deu de La Merced): Misericordia Floriach Robert, Celia Mareca Viladrich, Jesús Miguel Ruiz Idiago, Antonio Villa Riballo

Burgos (Servicio de Neurología Hospital General Yagüe): Esther Cubo, Cecilia Gil Polo, Natividad Mariscal

Perez, Jessica Rivadeneyra

Granada (Hospital Universitario San Cecilio, Neurología): Francisco Barrero, Blas Morales

Madrid-Clinico (Hospital Clínico Universitario San Carlos): María Fenollar, Rocío García-Ramos García, Paloma Ortega, Clara Villanueva

Madrid RYC (Hospital Ramón y Cajal, Neurología): Javier Alegre, Mónica Bascuñana, Juan Garcia Caldentey, Marta Fatás Ventura, Guillermo García Ribas, Justo García de Yébenes, José Luis López-Sendón Moreno, Patricia Trigo Cubillo

Madrid FJD (Madrid-Fundación Jiménez Díaz): Javier Alegre, Fernando Alonso Frech, Justo García de Yébenes, Pedro J García Ruíz, Asunción Martínez-Descals, Rosa Guerrero, María José Saiz Artiga, Vicenta Sánchez

Murcia (Hospital Universitario Virgen de la Arrixaca): María Fuensanta Noguera Perea, Lorenza Fortuna, Salvadora Manzanares, Gema Reinante, María Martirio Antequera Torres, Laura Vivancos Moreau

Oviedo (Hospital Central de Asturias): Sonia González González, Luis Menéndez Guisasola, Carlos Salvador, Esther Suaréz San Martín

Palma de Mallorca (Hospital Universitario Son Espases): Inés Legarda Ramirez, Aranzazú Gorospe, Mónica Rodriguez Lopera, Penelope Navas Arques, María José Torres Rodríguez, Barbara Vives Pastor

Pamplona (Complejo Hospitalario de Navarra): Itziar Gaston, Maria Dolores Martinez-Jaurrieta, Maria A. Ramos-Arroyo

Sevilla ("Hospital Virgen Macarena"): Jose Manuel Garcia Moreno, Carolina Mendez Lucena, Fatima Damas Hermoso, Eva Pacheco Cortegana, José Chacón Peña, Luis Redondo

Sevilla (Hospital Universitario Virgen del Rocío): Fátima Carrillo, María Teresa Cáceres, Pablo Mir, María José Lama Suarez, Laura Vargas-González

Valencia (Hospital la Fe): Maria E. Bosca, Francisco Castera Brugada, Juan Andres Burguera, Anabel Campos Garcia, Carmen Peiró Vilaplana

SWEDEN

Göteborg (Sahlgrenska University Hospital): Peter Berglund, Radu Constantinescu, Gunnel Fredlund, Ulrika Høsterey-Ugander, Petra Linnsand, Liselotte Neleborn-Lingefjärd, Jan Wahlström, Magnus Wentzel

Umeå (Umeå University Hospital): Ghada Loutfi, Carina Olofsson, Eva-Lena Stattin, Laila Westman, Birgitta Wikström

SWITZERLAND

Bern: Jean-Marc Burgunder, Yanik Stebler **(Swiss HD Zentrum)**, Alain Kaelin, Irene Romero, Michael

Schüpbach, Sabine Weber Zaugg **(Zentrum für Bewegungsstörungen, Neurologische Klinik und Poliklinik, Universität Bern)**

Zürich (Department of Neurology, University Hospital Zürich): Maria Hauer, Roman Gonzenbach, Hans H. Jung, Violeta Mihaylova, Jens Petersen

UNITED KINGDOM

Aberdeen (NHS Grampian Clinical Genetics Centre & University of Aberdeen): Roisin Jack, Kirsty Matheson, Zosia Miedzybrodzka, Daniela Rae, Sheila A Simpson, Fiona Summers, Alexandra Ure, Vivien Vaughan

Birmingham (The Barberry Centre, Dept of Psychiatry): Shahbana Akhtar, Jenny Crooks, Adrienne Curtis, Jenny de Souza (Keylock), John Piedad, Hugh Rickards, Jan Wright

Bristol (North Bristol NHS Trust, Southmead hospital): Elizabeth Coulthard, Louise Gethin, Beverley Hayward, Kasia Sieradzan, Abigail Wright

Cambridge (Cambridge Centre for Brain Repair, Forvie Site): Matthew Armstrong, Roger A. Barker, Deidre O'Keefe, Anna Di Pietro, Kate Fisher, Anna Goodman, Susan Hill, Ann Kershaw, Sarah Mason, Nicole Paterson, Lucy Raymond, Rachel Swain, Natalie Valle Guzman

Cardiff (Schools of Medicine and Biosciences, Cardiff University): Monica Busse, Cynthia Butcher, Jenny Callaghan, Stephen Dunnett, Catherine Clenaghan, Ruth Fullam, Olivia Handley, Sarah Hunt, Lesley Jones, Una Jones, Hanan Khalil, Sara Minster, Michael Owen, Kathleen Price, Anne Rosser, Jenny Townhill

Edinburgh (Molecular Medicine Centre, Western General Hospital, Department of Clinical Genetics): Maureen Edwards, Carrie Ho (Scottish Huntington´s Association), Teresa Hughes (Scottish Huntington´s Association), Marie McGill, Pauline Pearson, Mary Porteous, Paul Smith (Scottish Huntington´s Association)

Fife (Scottish Huntington's Association Whyteman's Brae Hospital): Peter Brockie, Jillian Foster, Nicola Johns, Sue McKenzie, Jean Rothery, Gareth Thomas, Shona Yates

Gloucester (Department of Neurology Gloucestershire Royal Hospital): Liz Burrows, Carol Chu, Amy Fletcher, Deena Gallantrae, Stephanie Hamer, Alison Harding, Stefan Klöppel, Alison Kraus, Fiona Laver, Monica Lewis, Mandy Longthorpe, Ivana Markova, Ashok Raman, Nicola Robertson, Mark Silva, Aileen Thomson, Sue Wild, Pam Yardumian

Hull (Castle Hill Hospital): Carol Chu, Carole Evans, Deena Gallentrae, Stephanie Hamer, Alison Kraus, Ivana Markova, Ashok Raman

Leeds (Chapel Allerton Hospital, Department of Clinical Genetics): Leeds (Chapel Allerton Hospital, Clinical Genetics): Carol Chu, Stephanie Hamer, Emma Hobson, Stuart Jamieson, Alison Kraus, Ivana

Markova, Ashok Raman, Hannah Musgrave, Liz Rowett, Jean Toscano, Sue Wild, Pam Yardumian

Leicester (Leicestershire Partnership Trust, Mill Lodge): Colin Bourne, Jackie Clapton, Carole Clayton, Heather Dipple, Dawn Freire-Patino, Janet Grant, Diana Gross, Caroline Hallam, Julia Middleton, Ann Murch, Catherine Thompson

Liverpool (Walton Centre for Neurology and Neurosurgery): Sundus Alusi, Rhys Davies, Kevin Foy, Emily Gerrans, Louise Pate

London (Guy's Hospital): Thomasin Andrews, Andrew Dougherty, Charlotte Golding, Fred Kavalier, Hana Laing, Alison Lashwood, Dene Robertson, Deborah Ruddy, Alastair Santhouse, Anna Whaite

London (The National Hospital for Neurology and Neurosurgery): Thomasin Andrews, Stefania Bruno, Karen Doherty, Charlotte Golding, Salman Haider, Davina Hensman, Nayana Lahiri, Monica Lewis, Marianne Novak, Aakta Patel, Nicola Robertson, Elisabeth Rosser, Sarah Tabrizi, Rachel Taylor, Thomas Warner, Edward Wild

Manchester (Genetic Medicine, University of Manchester, Manchester Academic Health Sciences Centre and Central Manchester University Hospitals NHS Foundation Trust): Natalie Arran, Judith Bek, Jenny Callaghan, David Craufurd, Ruth Fullam, Marianne Hare, Liz Howard, Susan Huson, Liz Johnson, Mary Jones, Helen Murphy, Emma Oughton, Lucy Partington-Jones, Dawn Rogers, Andrea Sollom, Julie Snowden, Cheryl Stopford, Jennifer Thompson, Iris Trender-Gerhard, Nichola Verstraelen (formerly Ritchie), Leann Westmoreland

Oxford (Oxford University Hospitals NHS Trust, Dept. of Neurosciences, University of Oxford): Richard Armstrong, Kathryn Dixon, Andrea H Nemeth, Gill Siuda, Ruth Valentine

Plymouth (Plymouth Huntington Disease Service, Mount Gould Hospital): David Harrison, Max Hughes, Andrew Parkinson, Beverley Soltysiak

Sheffield (The Royal Hallamshire Hospital– Sheffield Children's Hospital): Oliver Bandmann, Alyson Bradbury, Paul Gill, Helen Fairtlough, Kay Fillingham, Isabella Foustanos, Mbombe Kazoka, Kirsty O'Donovan, Nadia Peppa, Cat Taylor, Katherine Tidswell, Oliver Quarrell

EHDN's associate site in Singapore: National Neuroscience Institute Singapore: Jean-Marc Burgunder, Puay Ngoh Lau, Emmanul Pica, Louis Tan

PREDICT-HD Investigators of the Huntington Study Group

Leadership and Infrastructure

Principal Investigator

Jane S. Paulsen	University of Iowa

PREDICT Steering Committee Members

2008-2014 3rd NIH grant

H. Jeremy Bockholt	University of Iowa
Thomas Brashers-Krug	University of Iowa
Phil Danzer	University of Iowa
Megan Smith	University of Iowa
Jeffrey D. Long	University of Iowa
Hans J. Johnson	University of Iowa
Amanda Miller	University of Iowa
Kelsey Montross	University of Iowa
Holly Westerveld	Brown University

2004-2007 2nd NIH grant and CHDI

Elizabeth Aylward	University of Washington, Seattle
Kevin Biglan	University of Rochester
Robi Blumenstein	HighQ/CHDI
Elise Kayson	University of Rochester
Hans Johnson	University of Iowa
Bernhard Landwehrmeyer	University of Ulm, Germany
Blair Leavitt	UBC, Canada
Marcy MacDonald	MGH, Boston
Kevin Duff	University of Iowa
James Mills	University of Iowa
Christopher Ross	Johns Hopkins University
Ethan Singner	HighQ
Margaret Sutherland	NINDS
Dan Van Kammen	HighQ/CHDI

1999-2003 1st NIH grant

Elizabeth Aylward	University of Washington, Seattle
Mark Guttman	University of Toronto, Canada
Michael Hayden	University of British Columbia, Canada
Elise Kayson	University of Rochester
Karl Kieburtz	University of Rochester
Douglas Langbehn	University of Iowa
Martha Nance	Hennepin Hospital, Minneapolis
David Oakes	University of Rochester
Christopher Ross	Johns Hopkins University
Aileen Shinaman	University of Rochester
Ira Shoulson	University of Rochester
Julie Stout	Indiana University, Bloomington

2014-2017 (Leadership for Ancillary Grants)

H. Jeremy Bockholt	University of Iowa
Hans Johnson	University of Iowa
Daniel Sewell	University of Iowa
Ying Zhang	Indiana University

Roland Zschiegner	University of Iowa
Kelsey Montross	University of Iowa
Deborah Harrington	UCSD
Jessica Turner	Georgia State University

Core Sections

Biomarker Working Group

Jane Paulsen	University of Iowa
Stefano DiDonato	Fondazione IRCCS Institute of Neurology
Ken Evans	Ontario Cancer Biomarker Network
Daniel Chelsky	Caprion
Rick Meyers	Boston University
Dean Jones	Emory University
Clemens Scherzer	MGH
Andrew Juhl	University of Iowa
Beth Borowsky	CHDI
Blair Leavitt	Centre for Molecular Medicine/Therapeutics, UBC
Wayne Mattson	E N Rodgers Mem Hospital & Bedford VAMC
Asa Petersen	Lund University
Sarah Tabrizi	National Hospital for Neurology Neurosurgery UK
David Weir	UBC

Brain Donation Working Group

Jane Paulsen	University of Iowa
Anne Leserman	University of Iowa
Carol Moscowitz	Columbia University
Jean Paul G. Vonsattel	Columbia University
Stacie Vik	University of Iowa

Cognitive Coordination Centers:

Deborah Harrington	UCSD
Tamara Hershey	WUSL
Leigh Beglinger, Kevin Duff, Megan Smith	University of Iowa
Holly Westervelt	Brown University
Geoff Tremont	Brown University
Mary Gover	Brown University
Melanie Faust	Rhode Island Hospital
Greg Elias	Rhode Island Hospital
Jennifer Davis	Brown University
Susan Bonner	Rhode Island Hospital
Rachel Bernier	Rhode Island Hospital
Julie C. Stout	Indiana Univeristy
Noelle Carlozzi	Indiana Univeristy
Shannon A. Johnson	Indiana Univeristy
Scott A. Wylie	Indiana Univeristy
J. Colin Campbell	Indiana Univeristy
Eric J. Peters	Indiana Univeristy

Petra Theiner-Schumacher	Indiana Univeristy
Heather Coates	Indiana Univeristy
Sarah Queller	Indiana Univeristy
David Caughlin	Indiana Univeristy
Terren Green	Indiana Univeristy
Shelley Swain	Indiana Univeristy
Bethany Ward-Bluhm	Indiana Univeristy
Shaun Siegler	Indiana Univeristy

Biomedical Informatics

H. Jeremy Bockholt	University of Iowa
Roland Zschiegner	University of Iowa
Paul Allen	University of Iowa
Sudharshan Reddy Bommu	University of Iowa
Erin Carney	University of Iowa
Robert Connell	University of Iowa
Dan Kitzman	University of Iowa
Bill McKirgan	University of Iowa
Karen Pease	University of Iowa
Benjamin Rogers	University of Iowa
Adam Scott	University of Iowa
Mark Scully	University of Iowa
Jim Smth	University of Iowa
Austin Suiter	University of Iowa
Kent Williams	University of Iowa
Shuhua Wu	University of Iowa
Ryan Wyse	University of Iowa

Ethics

Cheryl Erwin	University of Iowa, U TX, Houston
Patricia Backlar	Portland State university
Debbie Bury	Chicago-Kent College of Law
Jim Calhoun	President, HDSA Georgia
Vicki Hunt	Wake Forest University
H. Jeremy Bockholt	University of Iowa
Thomas Brashers-Krug	University of Iowa
Janet K. Williams	University of Iowa
Martha Nance	Hennepin Hospital, Minneapolis
Jason Evans	University of Iowa
Kimberly Quaid	Indiana University, Indianapolis
Steven Hersch	Emory University
Lisa Hughes	University of Texas, Houston
Carl Leventhal	Indiana University
Bernard Lo	University of CA
Erik Parens	The Hastings Center
Harold Shapiro	Princeton University
Aileen Shinaman	University of Rochester
Alice Wexler	UCLA
Nancy Wexler	Columbia University
Jane Paulsen	University of Iowa

Iowa Study Coordination Center:

Elizabeth Penziner	University of Iowa
Christine Werling	University of Iowa
Karla Anderson	University of Iowa
Craig Stout	University of Iowa
Kristine Bjork	University of Iowa
LeeAnn Davis	University of Iowa
Ann Dudler	University of Iowa
Jamy Schumacher	University of Iowa
Steve Blanchard	University of Iowa
Phil Danzer	University of Iowa
Kelsey Montross	University of Iowa
Brenda Humble	University of Iowa
Steve Blanchard	University of Iowa
Bryan Ludwig	University of Iowa
Anne Leserman	University of Iowa
Lynda Sherman	University of Iowa
Carissa Nehl, BS	University of Iowa
Jane Paulsen	University of Iowa
Daniel Fernandez-Baca	University of Iowa
Gloria Ellis	University of Iowa
Stacie Vik	University of Iowa

MGH DNA Lab:

Marcy MacDonald	MGH, Harvard University, Boston
James Gusella	MGH, Harvard University, Boston

HSG Clinical Trials Coordination Center:

Keith Bourgeois	University of Rochester
Catherine Covert	University of Rochester
Susan Daigneault	University of Rochester
Karl Kieburtz	University of Rochester
Elise Kayson	Elise Kayson, MS, RNC
Elaine Julian-Baros	University of Rochester
Beverly Olsen	University of Rochester
Constance Orme	University of Rochester
Tori Ross	University of Rochester
Karen Rothenburgh	University of Rochester
Joseph Weber	University of Rochester
Hongwei Zhao	University of Rochester

IMAGING Working Group:

Elizabeth Aylward	University of Washington, Seattle
Vince Magnotta	University of Iowa
Hans Johnson	University of Iowa
Christopher Ross	Johns Hopkins University
Stephen Rao	Cleveland Clinic, Ohio
Jeremy Bockholt	University of Iowa
Peg Nopoulos	University of Iowa
Jatin Vaidya	University of Iowa
Deborah Harrington	UCSD

Andy Feigen	North Shore University
David Eidelberg	North Shore University
Vince Calhoun	MIND Research Network, UNM, GSU
Jessica Turner	Georgia State University
Michael Miller	Johns Hopkins University
CF Westin	Harvard Medical School
Guido Gerig	Utah Center for Neuroimaging / NYU

Cognitive Working Group

Peter Como	University of Rochester
Julie Stout	Indiana University
Leigh Beglinger	University of Iowa
Susan Bonner	Rhode Island Hospital
Noelle Carlozzi	Indiana University/ University of Michigan
Gabriel Castillo	UCSD
Jennifer Davis	Brown University
Michael Diaz	WUSL
Ian Dobbins	WUSL
Kevin Duff	University of Iowa/University of Utah
Greg Elias	Rhode Island Hospital
Melanie Faust	Rode Island Hospital
Erin Foster	WUSL
Carissa Gehl	VAMC Iowa City
Nellie Georgiou-Karistianis	University of Melbourne
Deborah Harrington	UCSD
Tamara Hershey	WUSL
Herwig Lange	Air-Rahazentrum
Kirsty Metheson	University of Aberdeen
Deborah Moore	WUSL
Jessica Morison	UCSD
KC Rowe	Univrsity of Iowa
David Moser	University of Iowa
Karen Siedlecki	Fordham University
Kate Papp	Brown University
Peter Snyder	Brown University
Jason Reed	UCSD
Megan Smith	University of Iowa
Danielle Theriault	University of Iowa
Geoff Tremont	Brown University
Natalie Valle Guzman	John vanGeest Center for Brain Repair
Carol Manning	University of Virginia
Randi Jones	Emory Universtiy
Holly Westerveld	Brown University

Recruitment Retention Committee:

Michelle Fox	University of California, Los Angeles
Elise Kayson	University of Rochester
Elaine Julian-Baros	University of Rochester
Martha Nance	Park Nicollet Clinic
Jane Paulsen	University of Iowa
Elizabeth Penziner	University of Iowa
Kimberly Quaid	Indiana University

Amanda Barnes	Johns Hopkins University
Greg Suter	Hereditary Neurological Disease Centre
Andrea Zanko	University of California San Francisco
Randi Jones	Emory University
Melinda Kavanaugh	Washington University
Hillary Lipe	University of Washington
Terry Tempkin	University of California Davis
Stacy Vik, BA	University of Iowa
Rachel Zombor	Graylands, Selby-Lemnos, Perth
Sean Thompson	University of Iowa
Katrin Barth	University of Ulm
Jenny Naji	Cardiff University
Jane Griffith	Westmead Hospital
Norman Reynolds	Medical College of Wisconsin

Statistics Working Group:

Jeffrey Long	University of Iowa
Ji-In Kim	University of Iowa
Wenjing Lu	University of Iowa
James Mills	University of Iowa
Blair Harrison	University of Iowa
Spencer Lourens	University of Iowa
Dawei Liu	University of Iowa
Ying Zhang	University of Iowa
Kai Wang	University of Iowa
Douglas Langbehn	University of Iowa
Hongwei Zhao	Texas A&M University
David Oakes	Unifersity of Rochester

**PREDICT-HD Investigators, Coordinators, Motor Raters, Cognitive Raters,**
**University of Iowa:**
Robert Rodnitzky, Henry Paulson, Ergun Uc, Justin Smock, Jessica Wood, Thomas Brashers- Krug, Jess Fiedorowicz, Eric Epping, Leigh Beglinger, Kevin Duff, Megan Smith, Jane Paulsen, Carissa Nehl Gehl, Karin Hoth, Clare Hey, Elizabeth Penziner, Beth Turner, Lynn Vining, Ania Mikos, Becky Reese, Rachel Conybeare, Sara Vander Heiden, Stacie Vik, Isabella De Soriano, Jessica Schumacher, Erica Wagner, Kimberly Bastic, Michelle Benjamin,Nancy Hale, Katie Hall, Mycah Kimble, Harisa kuburas, Jeremy Hinkel, Andrew Juhl, Stephen Cross, Angel Dominguez, Mackenzie Elbert, Terry Hayes, Jolene Luther, Amanda Miller, Pat Ryan, Emily Shaw, Kelli Thumma, Owen Wade, Courtney Hobart
**University of Melbourne, Australia:**
Edmond Chiu, Phyllis Chua, Joy Preston, Anita Goh, Olga Yastrubetskaya, Samantha Loi, Stephanie Antonopoulos Andrew Gibbs, Phillip Dingjan, Kristy Draper, Chathushka Fonseka,Nellie Georgiou-Karistianis, Christel Lemmon, Liz Ronsisvalle, David Ames, John Lloyd, Angela Komiti, Andrew Gibbs
**University of British Columbia, Vancouver:**

Lynn Raymond, Joji Decolongon, Mannie Fan, Allison Coleman, Elisabeth Almqvist, Kimberley Carter, Rachelle Dar Santos, Jordana Hutchinson, David Weir, Blair Leavitt
**Johns Hopkins University, Baltimore, MD:**
Christopher Ross, Adam Rosenblatt, Lisa Gourley, Arnold Bakker, Robin Miller, Barnett Shpritz, Mark Varvaris, Abhijit Agarwal, Gregory Churchill, Maryjane Ong, Meeia Sherr, Kristine Wajda,Claire Welsh, Nadine Yoritomo, Jason Brandt, Carolin Eschenbach
**Hereditary Neurological Disease Centre, Wichita, KS:**
William M. Mallonee, Greg Suter, Judy Addison, David Palmer
**University of Washington & VA Puget Sound Health Care System, Seattle, WA:**
Ali Samii, Hillary Lipe, Rebecca Logsdon, Kurt Weaver, Thomas Bird, Rosalynn DeLeon, EmilyFreney, Alma Macaraeg
**Emory University School of Medicine, Atlanta, Georgia**
Randi Jones, Joan Harrison, Stewart Factor, Cora Bush, Janet Cellar, Carol Ingram, Cathy Wood-Siverio, Timothy Greenamyre, Claudia Testa
**John van Geest Centre for Brain Repair, Cambridge, UK**
Roger Barker, Sarah Mason, Emma Smith, Natalie Valle Guzman, Gemma Cummins, Anna Di Pietro, Rachel Swain, Anna Goodman
**Westmead Hospital, Wentworth Ville, Sydney, Australia:**
Elizabeth McCusker, Bernadette Bibb, Clement Loy, Catherine Hayes, Kylie Richardson, Jane Griffith, David Gunn, Jillian McMillan
**University of Ulm, Germany:**
Bernhard Landwehrmeyer, Michael Orth, Katrin Barth, Carolin Eschenbach, Christine Held, Daniela Schwenk, Daniel Ecker, Anke Niess, Sonja Trautmann, Sigurd Suessmuth, Patrick Weydt
**Indiana University School of Medicine, Indianapolis:**
Kimberly Quaid, Melissa Wesson, Kathy Fleming, Jamalynne Stuck, Xabier Beristain, Joanne Wojcieszek
**Centre for Addiction and Mental Health University of Toroto, Ontario, Canada**
Mark Guttman, Rosa Ip, Alanna Scheinberg, Deanna Shaddick, Janice Stober, Rustom Sethna, Catherine Brown, Sheryl Elliott, Zelda Fonariov, Christine Giambattista, Albie Law, Irita Karmalkar, Sandra Russell, Joseph Sebastian, Adam Singer
**University of California, Los Angeles Medical Center, CA**
Susan Perlman, Laurie Carr, Russell Carroll, Brian Clemente, Arik Johnson, George Jackson, Xabier Beristain
**University of California San Francisco:**
Michael Geschwind, Joel Kramer, Sharon Sha, Margaret Wetzel, Jonathan Gooblar, Mira Guzijan, Katherine Rose, Gabriela Satris, Joseph Winer, Christina Wyss-Coray
**National Hospital for Neurology and Neurosurgery, London, UK:**
Tom Warner, Stefan Kloppel, Charlotte Golding, Thomasin Andrews, Eirini Kalliolia, Elisabeth Rosser, Sarah Tabrizi, Maggie Burrows
**Cardiff University, Cardiff, Wales, UK:**

Anne Rosser, Olivia Jane Handley, Jenny Naji, Catherine Johnston, Sarah Hunt, Kathy Price

University of Rochester, Rochester, New York, USA:

Peter Como, Frederick Marshall, Charlyne Hickey, Amy Chesire, Carol Zimmerman, Christina Burton, Mary Wodarski, Timothy Couniham

Neuroscience Unit, Graylands, Selby-Lemnos & Spec. Care Health Services, Perth, Australia

Peter Panegyres, Rachel Zombor, Brenton Maxwell, Maria Tedesco, Elizabeth Vuletich, Mark Woodman, Joseph Lee

Washington University – St. Louis, MO, USA

Joel Perlmutter, Stacey Barton, Melinda Kavanaugh, Amy Schmidt, Shineeka Smith, Lori McGee-Minnich

Clinical Genetics Centre, Aberdeen, Scotland, UK

Sheila Simpsom, Zosia Miedzybrodzka, Jackie Hamilton, Mariella D'Alessandro, Lorna Downie, Gwen Keenan, Kirsty Matheson, Daniella Rae, Alexandra Ure, Vivien Vaughan

University of Manchester, Manchester, UK:

David Craufurd, Jenny Callaghan, Elizabeth Howard, Judith Bek, Ruth Fullam, Rhona Macleod, Andrea Sollom

Columbia University Medical Center, New York, NY, USA:

Pietro Mazzoni, Karen Marder, Paula Wasserman, Jennifer Williamson, Paula Leber

Colorado Neurological Institute, Englewood, Colorado, USA:

Rajeev Kumar, Lauren Seeberger,Carolyn Greenwald, Melissa Holtgrewe, Breanna Nickels, Jay Schneiders, Christopher O'Brien, Colleen Dingmann, Diane Erickson, Carolyn Greenwald, Deborah Judd, Terri Lee Kasunic, Kristi Malleck, Lisa Mellick, Dawn Miracle, Sherrie Montellano, Christina Reeves, ,Alan Diamond

University of California – Davis, Sacramento, California, USA

Vicki Wheelock, Terry Tempkin, Kathleen Baynes, Sarah Farias, Lisa Kjer, Nicole Mans, Joseph Marsano, Amanda Martin

University of Alberta, Edmonton, Alberta, Canada:

Wayne Martin, Sheri Foster, Marguerite Weiler, Oksana Suchowersky, Satwinder Sran, Pamela King

Cleveland Clinic Foundation, Cleveland, Ohio, USA:

Anwar Ahmed, Jagan Pillai, Stephen Rao, Christine Reece, Justina Baryak, Alexandra Bea, Emily Newman, Alex Bura, Michael Lengen, Lyla Mourany, Juliet Schulz, Janice Zimbelman

Baylor College of Medicine, Waco, TX, USA

Tetsuo Ashizawa, Joseph Jankovic, Christine Hunter, Norma Cooke, Carrie Martin, Karinna Pacheco, George Ringholtz, Cynthia Studenko, William Ondo, Nicte Mejia, Kevin Dat Nguyen-Vuong, Lynn Ratkos

University of Calgary, Calgary, AB, Canada:

Oksana Suchowersky, Mary Lou Klimek, Dolen Kirstein, Sharon Lockey, Sarah Furtado,Anne Louise Lafontaine, Dwight Stewart

**Hospital Ramon y Cajal, Madrid, Spain**
Justo Garcia de Yebenes, Asuncion Martinez-Descales, Marta Fatas, Christine Schwartz,Javier Alegre, Monica Bascunana Garde
**Hennepin County Medical Center, Minneapolis, MN, USA**
Martha Nance, Dawn Radtke, Deanna Norberg, David Tupper
