# Supplementary material for: Exome sequencing of individuals with Huntington’s disease implicates FAN1 nuclease activity in slowing CAG expansion and disease onset
Source: Nat Neurosci. 2022 Apr 4;25(4):446–57. doi: 10.1038/s41593-022-01033-5 (PMC8986535; doi:10.1038/s41593-022-01033-5)
Supplement: Supplementary file 2 — Reporting Summary [file 41593_2022_1033_MOESM2_ESM.pdf]

nature research

Corresponding author(s): Lesley Jones and Thomas H. Massey

Last updated by author(s): Jan 28, 2022

Reporting Summary

Nature Research wishes to improve the reproducibility of the work that we publish. This form provides structure for consistency and transparency in reporting. For further information on Nature Research policies, see our Editorial Policies and the Editorial Policy Checklist.

Statistics

For all statistical analyses, confirm that the following items are present in the figure legend, table legend, main text, or Methods section.

n/a	Confirmed	
☐	☒	The exact sample size (*n*) for each experimental group/condition, given as a discrete number and unit of measurement
☐	☒	A statement on whether measurements were taken from distinct samples or whether the same sample was measured repeatedly
☐	☒	The statistical test(s) used AND whether they are one- or two-sided<br>*Only common tests should be described solely by name; describe more complex techniques in the Methods section.*
☐	☒	A description of all covariates tested
☐	☒	A description of any assumptions or corrections, such as tests of normality and adjustment for multiple comparisons
☐	☒	A full description of the statistical parameters including central tendency (e.g. means) or other basic estimates (e.g. regression coefficient) AND variation (e.g. standard deviation) or associated estimates of uncertainty (e.g. confidence intervals)
☐	☒	For null hypothesis testing, the test statistic (e.g. *F*, *t*, *r*) with confidence intervals, effect sizes, degrees of freedom and *P* value noted<br>*Give P values as exact values whenever suitable.*
☒	☐	For Bayesian analysis, information on the choice of priors and Markov chain Monte Carlo settings
☒	☐	For hierarchical and complex designs, identification of the appropriate level for tests and full reporting of outcomes
☐	☒	Estimates of effect sizes (e.g. Cohen's *d*, Pearson's *r*), indicating how they were calculated

Our web collection on statistics for biologists contains articles on many of the points above.

Software and code

Policy information about availability of computer code

Data collection: No code was used; all data was generated in the lab.

Data analysis: Whole exome sequences were aligned and variants called using a Genome Analysis Toolkit (GATK)-based pipeline (v3.6-0g89b7209; https://gatk.broadinstitute.org/hc/en-us), and variant annotation and quality control used Hail v0.1-5a67787 (https://hail.is/). SKAT-O analyses were performed in R using the SKAT package (v2.0.1; https://github.com/leeshawn/SKAT/), and MiSeq data used ScaleHD (0.322; https://scalehd.readthedocs.io/en/latest/). Analyses of genescan data used Autogenescan (v1.5.1; https://github.com/BranduffMcli/AutoGenescan), which uses the Fragman package (v1.0.9; Giovanny Covarrubias-Pazaran et al. 2016, BMC Genetics 17: 62), and GeneMapper v4.1 (Applied Biosystems). Details and versions of all software packages used are provided in Supplementary Table 10.

For manuscripts utilizing custom algorithms or software that are central to the research but not yet described in published literature, software must be made available to editors and reviewers. We strongly encourage code deposition in a community repository (e.g. GitHub). See the Nature Research guidelines for submitting code & software for further information.

Data

Policy information about availability of data

All manuscripts must include a data availability statement. This statement should provide the following information, where applicable:

- Accession codes, unique identifiers, or web links for publicly available datasets
- A list of figures that have associated raw data
- A description of any restrictions on data availability

Phenotypic data, variant call files (VCFs) and MiSeq data are available from the European Genome-Phenome Archive through a data access committee (EGA; ega-archive.org, accession number XXX). BAM files of exome sequencing data are available from EGA (REGISTRY-HD) or dbGaP (PREDICT-HD; ncbi.nlm.nih.gov/gap, accession number phs000371.v2.p1). Access to the EGA datasets is contingent on assurances that no attempt will be made to identify individual research

participants from genetic data. Biological materials derived in this work (edited iPSC lines, FAN1 expression plasmids) are available on request from the corresponding authors.

Field-specific reporting

Please select the one below that is the best fit for your research. If you are not sure, read the appropriate sections before making your selection.

☒ Life sciences ☐ Behavioural & social sciences ☐ Ecological, evolutionary & environmental sciences

For a reference copy of the document with all sections, see nature.com/documents/nr-reporting-summary-flat.pdf

Life sciences study design

All studies must disclose on these points even when the disclosure is negative.

Sample size	GeM-HD GWAS identified a number of genome-wide significant genetic modifiers of HD onset (GeM-HD Consortium, 2015). Using the effect size of the lead SNP a formal power calculation showed that exome-sequencing 40 individuals from each extreme of the residual age-at-onset distribution for a HD population of 7000 would have 88% power to detect a modifier variant with an equivalent effect size (5% significance level). The study was extended to include >700 exomes in the end, further increasing power.
Data exclusions	In exome sequencing quality control: exomes >3 standard deviations smaller than the mean of any of the three metrics were excluded for Registry-HD exomes. VerifyBamID76 was used to detect contamination, and samples with a Freemix > 0.075 were excluded, as per the ExAC study. Where there were duplicate samples, the exome with the highest coverage was retained. Sex imputation used Peddy; samples with conflicting imputed and recorded sex were excluded. One individual with originally unknown sex was kept. Ancestry was estimated using Peddy by principal component analysis (PCA) against genomes from the 1000 genomes project phase 379. Samples were excluded if they were either 1) predicted to have non-European ancestries by Peddy or 2) outside the primary cluster of European samples in Supplementary Fig. 1.
Replication	Exome sequencing was performed in two independent clinical samples (Registry-HD and Predict-HD) with consistent results. The two samples were not individually large enough to allow the detection of rare variants in one as a discovery dataset and the other as a replication dataset. Biochemical and cellular assays were repeated at least three times each on different days with fresh reagents, with two or three technical replicates of each individual data point. The results of all experimental replications of biochemical and cellular assays were consistent, and all data are presented in the paper.
Randomization	Randomisation was not relevant to this study. Individuals with HD were assigned to early/more severe and late/less severe phenotype groups on the basis of their clinical phenotypes. In Registry-HD individuals were stratified by residual age at motor onset and those at the extremes of the distribution selected for sequencing. In Predict-HD individuals were stratified by motor or cognitive scores, or predicted time to onset, and extremes selected for sequencing.
Blinding	Researchers were not blinded to clinical phenotype when performing exome/variant association analyses. For nuclease assays, the user was not blinded to FAN1 variant, but they were blinded to the phenotypes associated with patients harbouring that variant. For iPSC assays the user was not blinded to the cell line being used but CAG lengths were called by user-independent software to reduce the risk of peak calling bias.

Reporting for specific materials, systems and methods

We require information from authors about some types of materials, experimental systems and methods used in many studies. Here, indicate whether each material, system or method listed is relevant to your study. If you are not sure if a list item applies to your research, read the appropriate section before selecting a response.

Materials & experimental systems

n/a	Involved in the study
☐	☒ Antibodies
☐	☒ Eukaryotic cell lines
☒	☐ Palaeontology and archaeology
☒	☐ Animals and other organisms
☐	☒ Human research participants
☒	☐ Clinical data
☒	☐ Dual use research of concern

Methods

n/a	Involved in the study
☒	☐ ChIP-seq
☒	☐ Flow cytometry
☒	☐ MRI-based neuroimaging

Antibodies

Antibodies used	Anti-FAN1 (sheep polyclonal, 1:1000, CHDI); Anti-β-Tubulin Antibody, clone AA2 (mouse monoclonal, 1:10000, Upstate 05-661); Donkey anti-Mouse IgG (H+L) Highly Cross-Adsorbed Secondary Antibody, Alexa Fluor Plus 680 (donkey polyclonal, 1:10000, Invitrogen A32788); IRDye® 800CW Donkey anti-Goat IgG Secondary Antibody (donkey polyclonal, 1:15000, Licor 926-32214); Anti-OCT4 antibody (rabbit polyclonal, 1:100, AbCam ab19857); Anti-MAP2 antibody (rabbit polyclonal, 1:500, AbCam ab32454); Anti-

CTIP2 (Rat monoclonal 25B6, 1:200, AbCam ab18465); Alexa Fluor goat anti-mouse IgG 488 (goat polyclonal, 1:400, Invitrogen A11001); Alexa Fluor goat anti-rabbit IgG 568 (goat polyclonal, 1:800, Invitrogen A11011).

Validation

FAN1 antibody: see Goold et al., 2019 and CHDI website; Anti-β-Tubulin Antibody is validated for use in Western blotting, backed up by multiple publications (https://www.merckmillipore.com/GB/en/product/Anti-Tubulin-Antibody-clone-AA2,MM_NF-05-661#anchor_REF); Donkey anti-Mouse IgG (H+L) Highly Cross-Adsorbed Secondary Antibody, Alexa Fluor Plus 680 is validated for use as secondary antibody against all mouse immunoglobulins and cross-adsorbed against other species when purified. Cited in multiple publications (https://www.thermofisher.com/antibody/product/Donkey-anti-Mouse-IgG-H-L-Highly-Cross-Adsorbed-Secondary-Antibody-Polyclonal/A32788); IRDye® 800CW Donkey anti-Goat IgG Secondary Antibody specifically tested and qualified for use in Western blotting (https://www.licor.com/bio/reagents/irdye-800cw-donkey-anti-goat-igg-secondary-antibody); Anti-OCT4 antibody is validated for use in ICC and cited in over 450 papers (https://www.abcam.com/oct4-antibody-ab19857.html); Anti-MAP2 antibody is validated for use in ICC and cited in over 200 papers (https://www.abcam.com/map2-antibody-neuronal-marker-ab32454.html); Anti-CTIP2 is validated for use in ICC and cited in over 550 papers (https://www.abcam.com/ctip2-antibody-25b6-ab18465.html); Alexa Fluor goat anti-mouse IgG 488 is cross-adsorbed and validated for use in ICC, being cited over 5000 times (https://www.thermofisher.com/antibody/product/Goat-anti-Mouse-IgG-H-L-Cross-Adsorbed-Secondary-Antibody-Polyclonal/A-11001); Alexa Fluor goat anti-rabbit IgG 568 is cross-adsorbed and validated for use in ICC and cited over 1700 times (https://www.thermofisher.com/antibody/product/Goat-anti-Rabbit-IgG-H-L-Cross-Adsorbed-Secondary-Antibody-Polyclonal/A-11011).

Eukaryotic cell lines

Policy information about cell lines

Cell line source(s)

Human lymphoblastoid cell lines derived directly from HD patient samples were obtained from CHDI (lines generated and banked by BioRep, Italy). Human induced pluripotent stem cell (iPSC) lines, 109 CAGs, as reported in HD iPSC Consortium, 2012, Cell Stem Cell 11: 264-278 and Mattis et al., 2015, Human Molecular Genetics 24: 3257-3271. Lines available through CHDI.

Authentication

Lymphoblastoid cell lines derived directly from HD patient samples were received from BioRep, Italy and grown in the lab. HTT CAG repeat lengths were assayed regularly by PCR fragment analysis and by MiSeq and cross-referenced against expected repeat lengths from banked DNA samples from the same individuals. Q109 iPSCs were regularly sized for HTT CAG repeat length, had regular virtual karyotyping by CNV analysis, and were exome sequenced.

Mycoplasma contamination

Cell lines were regularly checked for mycoplasma contamination and were always negative

Commonly misidentified lines
(See ICLAC register)

No commonly misidentified cell lines were used in this project

Human research participants

Policy information about studies involving human research participants

Population characteristics

Both patient cohorts (REGISTRY-HD and PREDICT-HD) were studies of Huntington's disease patients. The main measure used both for analysis and selection of REGISTRY-HD patients was age at motor onset of disease. Using CAG length and the Langbehn model (Langbehn et al, 2004, Clinical Genetics 65:267-277) a residual age at onset was calculated. For PREDICT-HD, those who had phenoconverted to motor onset similarly had a residual age at onset calculated. In addition, predicted ages at onset were calculated as described in the manuscript. Extreme worst or best scorers for single digit modalities test (SDMT) or total motor score (TMS) were also determined as described. The predicted ages at onset and extreme SDMT/TMS scorers were used as binary measures as extreme worst/best measures alongside early/late onset individuals from Registry. Overall, 465 participants from REGISTRY-HD were included (53.4% female, mean age at onset = 48.4 years) and 218 participants from PREDICT-HD (61.9% female, mean age at onset = 47.1 years). See Supplementary Table 1 for details.

Recruitment

Patients were not recruited directly to this study. The REGISTRY-HD and PREDICT-HD populations were recruited over a number of years and followed longitudinally over time. Participants were research-motivated individuals carrying the HD mutation who provided detailed phenotypic information as well as DNA samples. They gave informed consent at the time of recruitment into the two studies for their data to be analysed by researchers at a later date. This study stratified the two populations as described (Fig. 1) and selected those with extremes of phenotype for exome sequencing. Selections were made on the basis of phenotypic analysis and sample availability and as such there were no inherent selection biases identified.

Ethics oversight

Ethical approval for REGISTRY-HD was obtained in each participating country. Investigation of deidentified PREDICT-HD subjects was approved by the Institutional Review Board of Partners HealthCare (now Mass General Brigham). Participants from both studies gave written informed consent. All experiments described herein were conducted in accordance with the declaration of Helsinki. Local ethical approval was through Cardiff University School of Medicine SMREC 19/55.

Note that full information on the approval of the study protocol must also be provided in the manuscript.
