# Supplementary figures and images for: Exome sequencing of individuals with Huntington’s disease implicates FAN1 nuclease activity in slowing CAG expansion and disease onset
Source: Nat Neurosci. 2022 Apr 4;25(4):446–57. doi: 10.1038/s41593-022-01033-5 (PMC8986535; doi:10.1038/s41593-022-01033-5)

Source data Fig. 4b

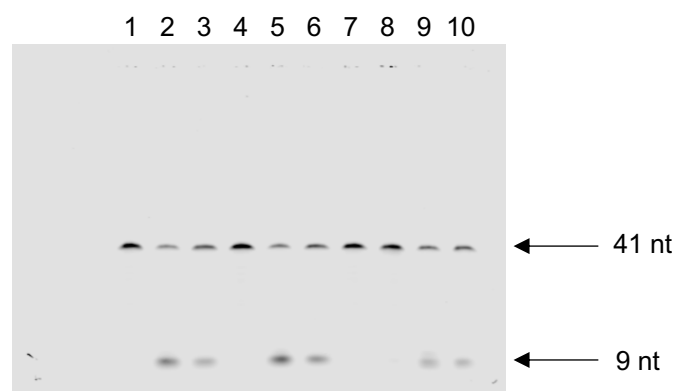

Note that lane 8 was omitted in Fig. 4b

Supplement: Source Data Fig. 4 — Unprocessed gel [file 41593_2022_1033_MOESM3_ESM.pdf]

Source data Fig. 5a

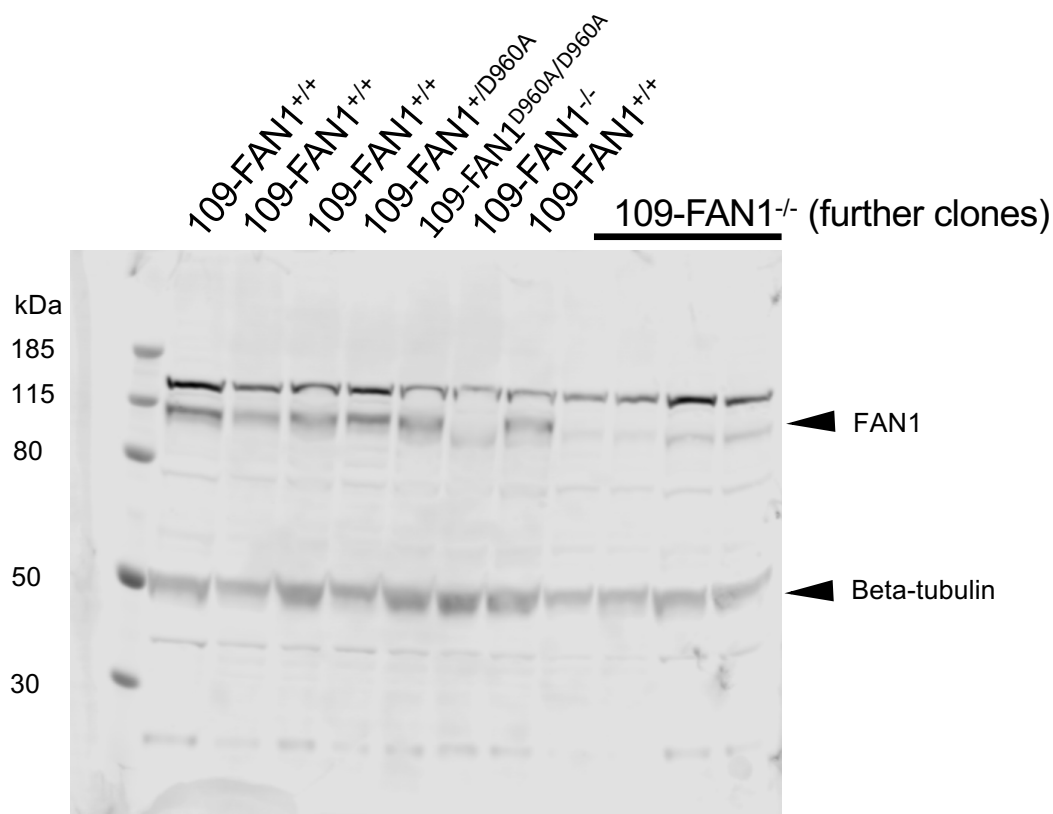

Supplement: Source Data Fig. 5 — Unprocessed immunoblot [file 41593_2022_1033_MOESM4_ESM.pdf]
