# Supplementary material for: Exome sequencing of individuals with Huntington’s disease implicates FAN1 nuclease activity in slowing CAG expansion and disease onset
Source: Nat Neurosci. 2022 Apr 4;25(4):446–57. doi: 10.1038/s41593-022-01033-5 (PMC8986535; doi:10.1038/s41593-022-01033-5)
Supplement: Source Data Extended Data Fig. 2 — Unprocessed gels [file 41593_2022_1033_MOESM5_ESM.pdf]

Source data for Extended Data Fig. 2

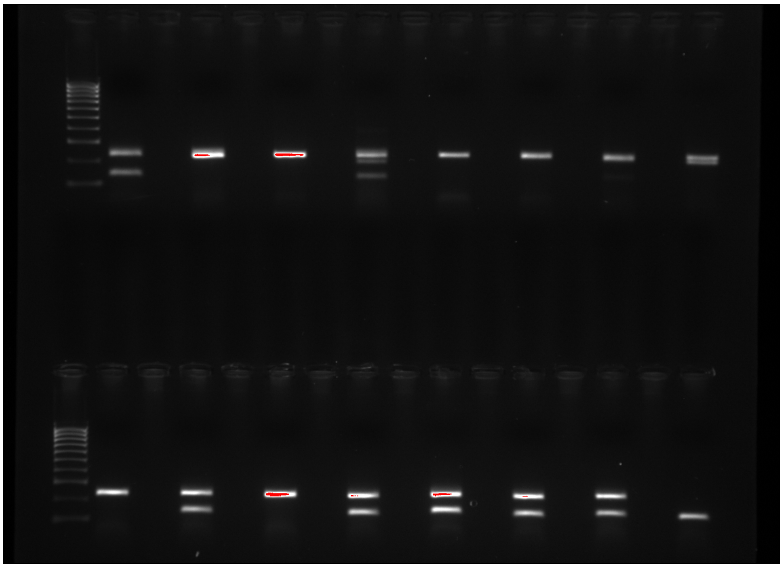

Agarose gel Extended Data Fig. 2b

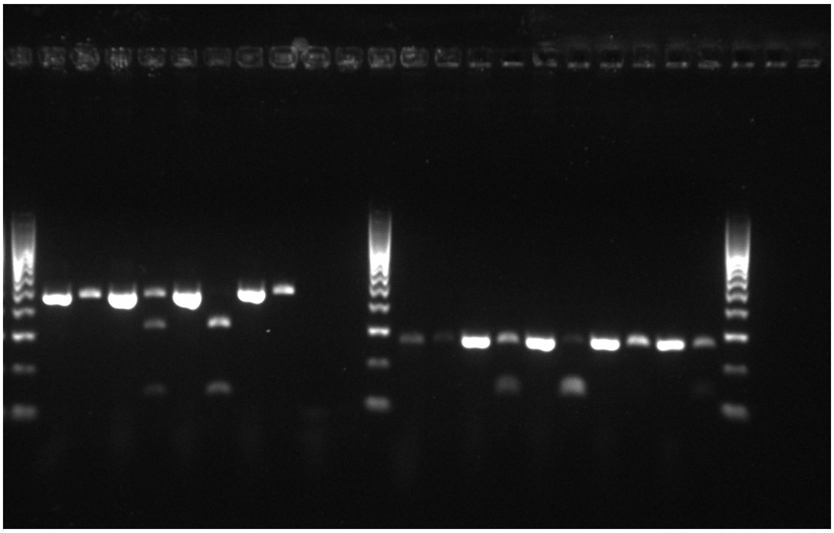

Agarose gel Extended Data Fig. 2f
